# Dimorpholinoacetylene and Its Use for the Synthesis of Tetraaminocyclobutadiene Species

**DOI:** 10.1002/chem.202202737

**Published:** 2022-11-03

**Authors:** Lukas Körner, Luong Phong Ho, Ralph Puchta, Amnon Stanger, Matthias Tamm

**Affiliations:** ^1^ Institut für Anorganische und Analytische Chemie Technische Universität Braunschweig Hagenring 30 38106 Braunschweig Germany; ^2^ Department Chemie und Pharmazie Friedrich-Alexander Universität Erlangen-Nürnberg Egerlandstr. 1 91058 Erlangen Germany; ^3^ Schulich Department of Chemistry, Technion Haifa 32000 Israel

**Keywords:** cyclic bent allenes, cyclobutadienes, diaminoacetylenes, four-membered rings, NICS

## Abstract

The new diaminoacetylene (DAA) dimorpholinoacetylene (**3**) was prepared from 1,1‐dimorpholinoethene (**1**) by bromination to form the dibromoketene aminal **2**, which upon lithiation afforded **3** through a Fritsch‐Buttenberg‐Wiechell rearrangement. Heating **3** at elevated temperatures resulted in a complete conversion into the dimer 1,1,2,4‐tetramorpholino‐1‐buten‐3‐yne (**4**), which was used for the synthesis of four‐membered cyclic bent allene (CBA) transition‐metal complexes of the type [(CBA)ML_n_] (**5**‐**7**; ML_n_=AuCl, RhCl(COD), RhCl(CO)_2_; CBA=1,3,4,4‐tetramorpholino‐1,2‐cyclobutadiene; COD=1,5‐cyclooctadiene). The reaction of **3** with tetraethylammonium bromide gave 1,2,3,4‐tetramorpholinocyclobutenylium bromide (**8**), which reacted with bromine to form 1,2,3,4‐tetra(morpholino)cyclobutenediylium bis(tribromide) (**9**). Compound **9** represents the first fully characterized compound containing a tetraaminocyclobutadiene dication and displays a nearly planar C_4_N_4_ core as shown by X‐ray diffraction analysis. Detailed quantum chemical calculations were performed to assess the aromaticity of tetraaminocyclubutadiene dications by employing the Nucleus Independent Chemical Shift (NICS) method and current density analysis.

## Graphical abstract

**Dimorpholinoacetylene** has been synthesized and structurally characterized as a new member of the class of electron‐rich diaminoacetylenes. The new compound can be used to produce cyclic bent allene (CBA) transition‐metal complexes or oxidized to give the first fully characterized cyclobutadiene dication. Detailed computational studies by NICS and current density calculations were carried out to assess the aromaticity of tetraaminocyclobutadiene dications.

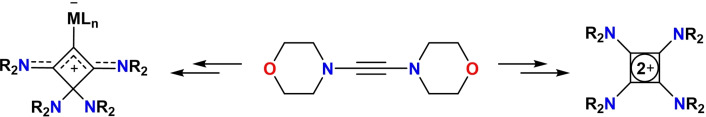

## Introduction

**Figure 1:**
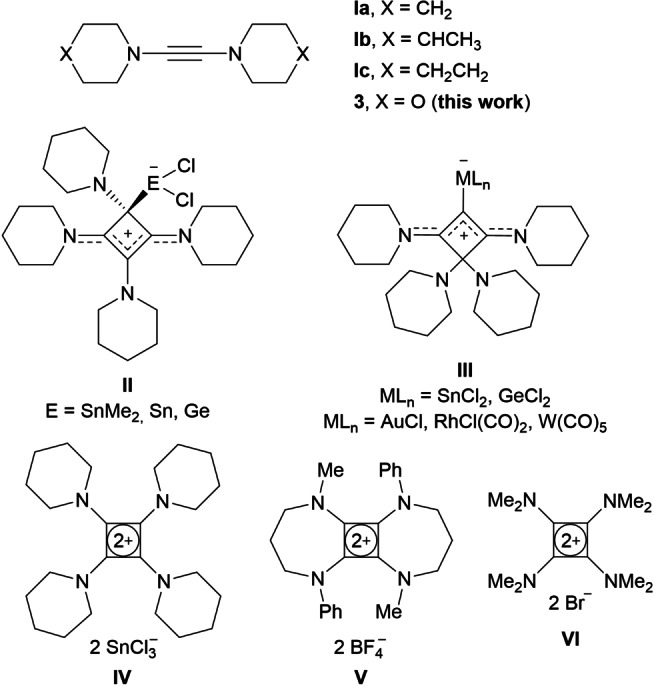
Selected diaminoacetylenes (DAAs) and tetraaminocyclobutadiene species.

1,2‐Diaminoalkynes (diaminoacetylenes, DAAs) represent an electron‐rich and highly reactive class of alkynes which, in contrast to tamed derivatives, such as 1‐amino‐ and 1‐amidoalkynes,^[1]^ has received comparatively little attention to date.^[2]^ This disregard is quite surprising, since Viehe and Reinstein reported on the synthesis of the first representative, bis(diethylamino)acetylene, as early as 1964;^[3]^ however, it can be ascribed to the rather laborious nature of the previous synthetic protocols, which generally involved the generation of halogen‐substituted alkynes and their treatment with several equivalents of alkalimetal amides.^[4]^ Better access to a series of DAAs of type **I** (Figure 1) was granted by the protocol introduced by our group in 2010,^[5]^ which involved the bromination of 1,1‐ethenediamines, followed by the treatment of the resulting 2,2‐dibromo‐1,1‐ethenediamines with *n*BuLi to form DAAs, including **Ia**–**Ic**, through a Fritsch‐Buttenberg‐Wiechell rearrangement.^[6]^ The coordination chemistry of these alkynes,^[2]^ predominantly dipiperidinoacetylene (**Ia**), towards transition metals^[7]^ and main group elements^[8]^ has been studied, and most of their reactivity can be attributed to their latent diamino‐dicarbene nature.^[9]^

Different reactivity was uncovered by the group of Holger Braunschweig,^[10]^ who reported the cyclization of **Ia** upon addition of Me_2_SnCl_2_, and similar 1,2,3,4‐tetraamino‐1,3‐cyclobutadiene adducts **II** were isolated from the reactions of **Ia** with SnCl_2_ or GeCl_2_ ⋅ dioxane, respectively.^[11]^ In this context, we also reported that **Ia** dimerizes under thermal conditions to the corresponding enyne, which afforded the isomeric SnCl_2_‐ and GeCl_2_‐stabilized 1,3,4,4‐tetraamino‐1,2‐cyclobutadiene species **III** under analogous reaction conditions. Related gold(I), rhodium(I), and tungsten(0) complexes of type **III** were also prepared (Figure 1),^[11]^ and these species represent members of the class of so‐called cyclic bent allenes (CBA),^[12]^ which have received considerable attention from a theoretical point of view and as strong donor ligands in transition‐metal chemistry and homogeneous catalysis.^[13]^ Four‐membered CBAs, however, are exceedingly rare,^[14]^ and apart from compounds **III**, the only other related system had previously been generated by Guy Betrand and coworkers from a 1,3‐diperidinocyclobutenylium salt by deprotonation.^[15]^

In the preparation of the tin compound **III** (ML_n_=SnCl_2_), we also observed that **Ia** can undergo a redox reaction with excess tin(II) chloride, and the tetrapiperidinocyclobutadiene dication with two trichlorostannate(I) counterions (compound **IV**, Figure 1) was isolated as a by‐product and characterized crystallographically.^[11]^ To the best of our knowledge, **IV** represents the only structurally authenticated salt containing a cyclobutadiene (CBD) dication, although related tetraamino derivatives such as **V** and **VI** had previously been reported by Siegfried Hünig^[16]^ and Heinz Günther Viehe.^[17]^

In the more than 120‐year history of the search for stable carbocations,^[18]^ CBD dications have received surprisingly little experimental attention, with the exception of the Nobel laureate George A. Olah, whose group described the generation and NMR spectroscopic characterization of the dicationic species C_4_Me_4_^2+^, C_4_Ph_4_^2+^, *cis*‐C_4_Ph_2_H_2_^2+^, and *cis*‐C_4_Ph_2_F_2_^2+^ in superacidic media in the temperature range between −78 and −60 °C.^[19]^ Although classical Hückel rule considerations suggest square‐planar geometries for these species and thus *D*_4h_ symmetry in case of C_4_H_4_^2+^,^[20]^ theoretical calculations predict folded *D*_2d_ geometries for the dications C_4_R_4_^2+^ (R=H, Me), which has been ascribed to the relief of destabilizing in‐plane 1,3‐interactions and subsequent stabilization of the puckered form by orbital mixing and orbital reorientation.[^21^, ^22^, ^23^, ^24^, ^25^] In case of C_4_Ph_4_^2+^, C_4_F_4_^2+^ and C_4_(CN)_4_^2+^, however, ring planar minimum geometries (dihedral angle of 0°) were theoretically predicted due to possible delocalisation of the positive charge on the substituents.^[26]^ Accordingly, planar (*C*_4h_) geometries were recently confirmed for the dications in the salts [C_4_(OH)_4_][MF_6_]_2_ ⋅ 2HF (M=As, Sb) by single‐crystal X‐ray structure analyses,^[27]^ which is in line with earlier predictions for diprotonated squaric acid C_4_(OH)_4_^2+^.^[28]^ A planar geometry was also predicted for the tetraaminocyclobutadiene dication, C_4_(NH_2_)_4_^2+^, based on semi‐empirical quantum chemical calculations,^[29]^ in contrast to the experimentally observed slightly puckered structure established for the tetrapiperidino derivative **IV**.^[11]^

With this contribution, we would like to introduce dimorpholinoacetylene (**3**, Figure 1) as a new member of the still small DAA family by reporting on its synthesis and characterization, including an X‐ray crystal structure analysis. Furthermore, the reactivity of **3** has been investigated, with its dimerization to the corresponding 1,1,2,4‐tetramorpholino‐1‐buten‐3‐yne (**4**) allowing access to 1,2‐cyclobutadiene metal complexes, while its protonation and subsequent oxidation with bromine affords the tribromide salt [C_4_{N(CH_2_CH_2_)_2_O}_4_][Br_3_]_2_ (**9**) with a tetramorpholinocyclobutadiene dication, that has a planar C_4_ ring in the solid state.

## Results and Discussion

### Synthesis and characterization of dimorpholinoacetylene and its derivatives

**Scheme 1:**
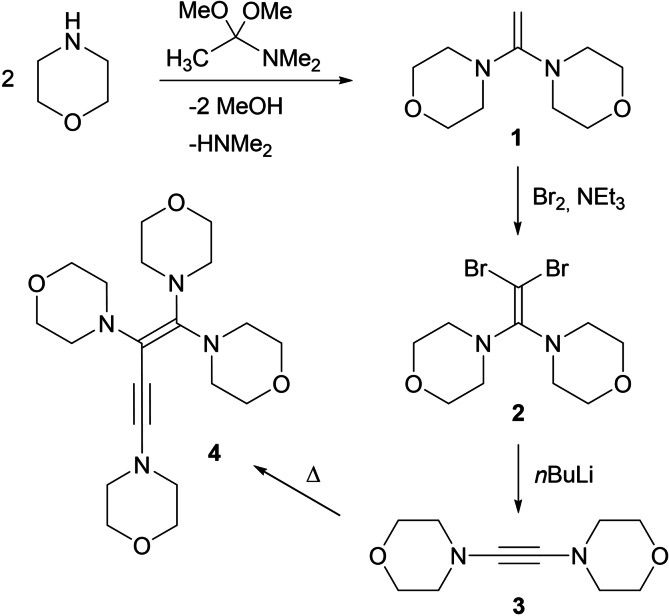
Synthesis and dimerization of dimorpholinoacetylene (**3**).

Reaction of *N*,*N*‐dimethylacetamide dimethyl acetal with an excess of morpholine at elevated temperature (110–150 °C) afforded 1,1‐dimorpholinoethene (**1**) in 80 % yield as an orange solid in analogy to previous protocols (Scheme 1).^[30]^ The ^1^H NMR spectrum (in CDCl_3_) exhibits the expected two triplets (^3^ *J*_H‐H_=4.6 Hz) at 2.86 and 3.68 ppm for the NCH_2_ and OCH_2_ hydrogen atoms together with a singlet at 3.30 ppm for the olefinic CH_2_ group. In the ^13^C{^1^H} NMR spectrum, the signals at 70.4 (C=*C*H_2_) and 162.3 ppm (*C*=CH_2_) are assigned to the olefinic carbon atoms, indicating the strong polarization of the carbon‐carbon double bond. The subsequent bromination of **1** in dichloromethane (DCM) in the presence of triethylamine proceeded cleanly and afforded the dibromoketene aminal **2** as a light‐yellow solid in 82 % after evaporation and extraction with toluene. The ^1^H NMR spectrum (in C_6_D_6_) exhibits the two expected triplets (^1^ *J*_H‐H_=4.6 Hz) for the remaining CH_2_ groups at 2.71 and 3.31 ppm, while the olefinic carbon atoms give rise to ^13^C NMR signals at 55.2 (CBr_2_) and 153.6 ppm (CN_2_). It should be noted that **2** should not be stored in solution for prolonged time, since decomposition and formation of the hydrobromide **2** ⋅ HBr (**S1**) was repeatedly observed (see the Supporting Information for an X‐ray crystal structure of **S1** ⋅ CHCl_3_).

To trigger the Fritsch‐Buttenberg‐Wiechell rearrangement and formation of DAA **3**, the dibromide **2** was initially treated with *n*‐butyllithium (*n*BuLi) in toluene solution, however, **3** was isolated in only small yield up to 20 %. The observation of significant amounts of a poorly soluble precipitate raised the suspicion that a lithium coordination polymer might have formed through the presence of the morpholine oxygen atom. Therefore, two equivalents of 1,4‐dioxane were added to the reaction mixture, and indeed, **3** could be isolated in satisfactory yield (60 %) as a colorless solid after recrystallization from THF/*n*‐hexane. The ^1^H NMR spectrum of **3** shows the two expected multiplets for the morpholine units, while the ^13^C NMR signals of the acetylenic carbon atoms are found at 73.9 ppm, in good agreement with the chemical shifts reported for **Ia** (74.8 ppm), **Ib** (75.0 ppm), and **Ic** (77.5 ppm).^[5]^

**Figure 2:**
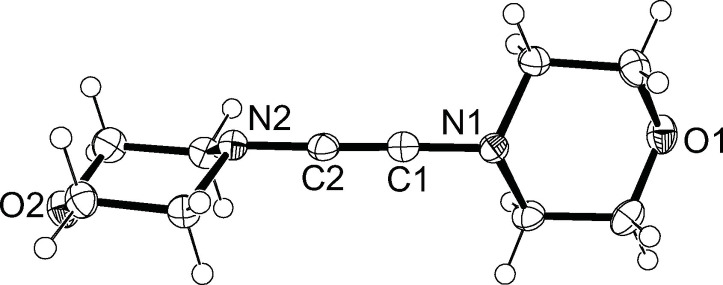
Molecular structure of **3** with thermal displacement parameters drawn at 50 % probability; selected bond lengths [Å] and angles [°]: C1−C2 1.199(1), C1−N1 1.361(1); C2−C1−N1 178.3 (1), C1−C2−N2 177.2(1).

The molecular structure of **3** could be determined by single‐crystal X‐ray diffraction analysis (Figure 2). Overall, the structural parameters are similar to those established for DAA **Ib**,^[5]^ with the molecule displaying a linear N1−C1−C2−N2 axis with N1−C1−C2 and N2−C2−C1 angles of 178.3(1)° and 177.2(1)°, respectively. The carbon‐carbon triple bond lengths of 1.199(1) and 1.206(2) Å in the ynediamines **3** and **Ib** are almost identical, and a similar range has also recently been reported for a series of ynediamides.^[31]^ Noteworthy, the two morpholino units are highly twisted and adopt nearly perpendicular orientations to each other with a dihedral angle of 82.6(2)° between the C1−N1−O1 and C2−N2−O2 planes. This conformation is consistently found in ynediamines^[5]^ and ynediamides^[31]^ and was also calculated to be more favorable for the parent DAA H_2_NC≡CNH_2_.^[32]^ The nitrogen atoms in **3** display distinctly trigonal‐pyramidal environments with angle sums of 339.5° (N1) and 338.1° (N2), which excludes significant π interaction with the C−C triple bond.

**Figure 3:**
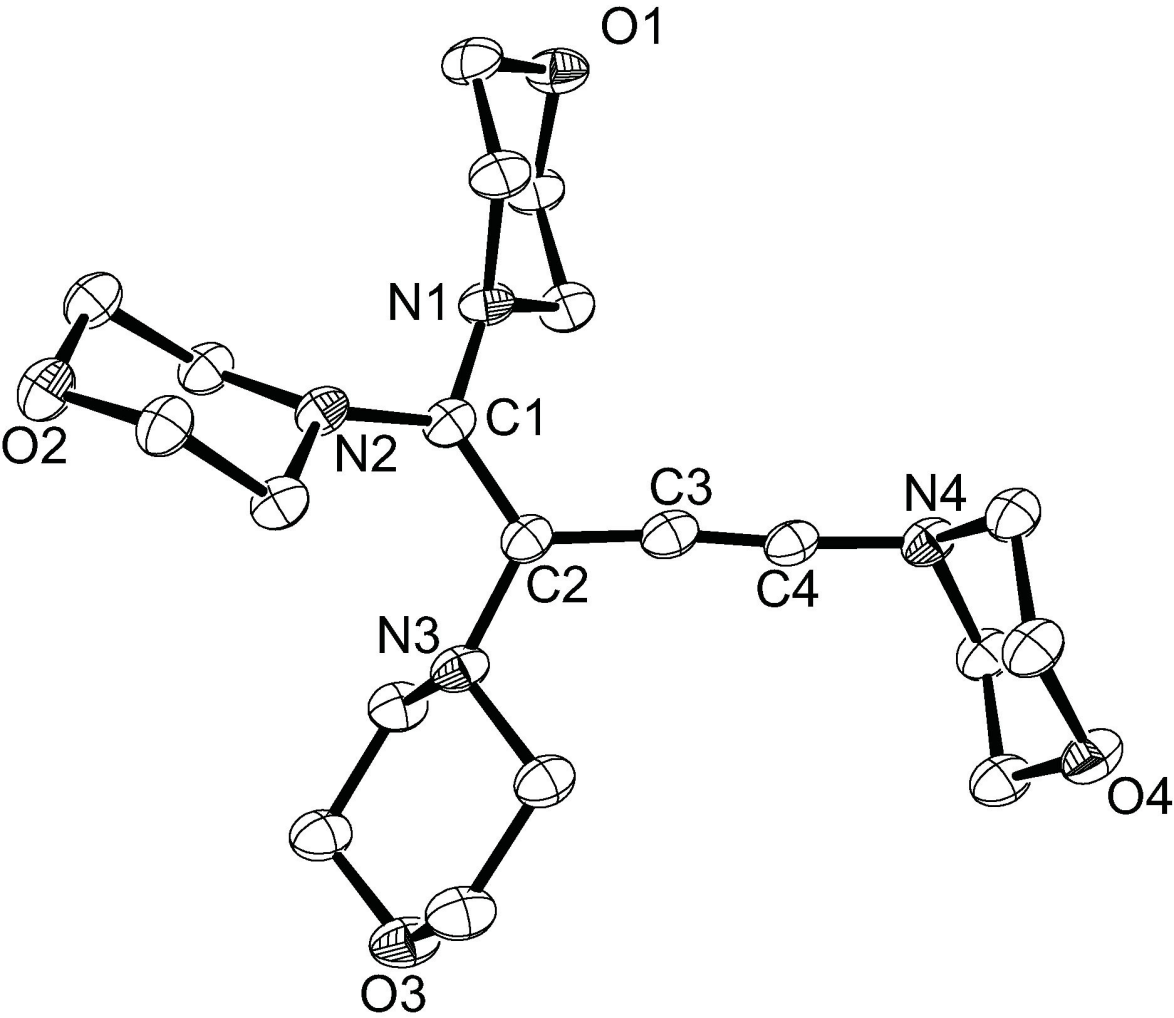
Molecular structure of **4** with thermal displacement parameters drawn at 50 % probability; hydrogen atoms and a second independent molecule in the asymmetric unit are omitted for clarity. Selected bond lengths [Å] and angles [°] in molecule 1/molecule 2: C1−C2 1.375(3)/1.362(4), C2−C3 1.421(4)/1.423(4), C3−C4 1.210(4)/1.213(4), C1−N1 1.399(4)/1.405(3), C2−N3 1.441(4)/1.446(3), C4−N4 1.355(4)/1.355(3); C3−C4−N4 175.3(3)/178.2(3), C1−C2−C3 122.1(3)/122.4(2), N1−C1−N2 112.3(2)/112.3(2).

We recently discovered that dipiperidinoacetylene (**Ia**) dimerizes when heated in pure form at elevated temperature to form the corresponding 1,1,2,4‐tetrapiperidino‐1‐buten‐3‐yne in almost quantitative yield.^[11]^ Based on DFT calculations, we were able to propose a plausible uncatalyzed mechanism for the dimerization of **Ia** that involves a 1,4‐dicarbene intermediate and a subsequent 1,3‐migration of one piperidino group.^[11]^ Since dimorpholinoacetylene (**3**) is a solid, its dimerization was accomplished by heating a concentrated toluene solution at 110 °C for 24 h, resulting in a complete conversion into 1,1,2,4‐tetramorpholino‐1‐buten‐3‐yne (**4**), which was isolated as a beige solid. Its ^1^H NMR spectrum shows eight multiplets in the range 2.5–3.8 ppm for the NCH_2_ and OCH_2_ hydrogen atoms of the four inequivalent morpholine rings, while the quaternary carbon atoms of the C=C−C≡C chain give rise to four ^13^C NMR signals at 155.4, 101.4, 58.5, and 101.1 ppm, which is in excellent agreement with the values reported for the corresponding piperidino derivative (157.8, 102.6, 59.9 and 101.8 ppm).^[11]^ Crystals suitable for X‐ray diffraction analysis were obtained from a saturated *n*‐hexane solution, and the resulting molecular structure is shown in Figure 3. The asymmetric unit contains two independent molecules, which mainly differ in the orientation of the morpholino substituent in the 4‐position, which bears the enyne substituent in the axial position only in molecule 1 (at N4 in Figure 3). The carbon‐carbon bond lengths within the C=C−C≡C chain are 1.375(3)/1.362(4) Å (C1−C2), 1.421(4)/1.423(4) Å (C2−C3), and 1.210(4)/1.213(4) Å (C3−C4), which again agrees well with the values reported for the piperidino congener.^[11]^ Likewise, both independent molecules are markedly twisted as for instance indicated by N2−C1−C2−N3 torsion angles of 25.7(4)° (molecule 1) and 24.8(4)° (molecule 2).

**Scheme 2:**
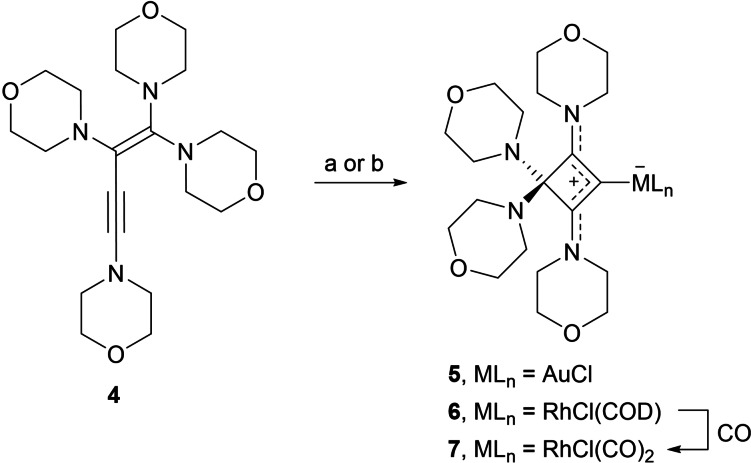
Synthesis of transition‐metal 1,2‐cyclobutadiene complexes; reagents: a) 1 equiv. [(THT)AuCl]; b) 0.5 equiv. [Rh(COD)(μ‐Cl)]_2_.

**Figure 4:**
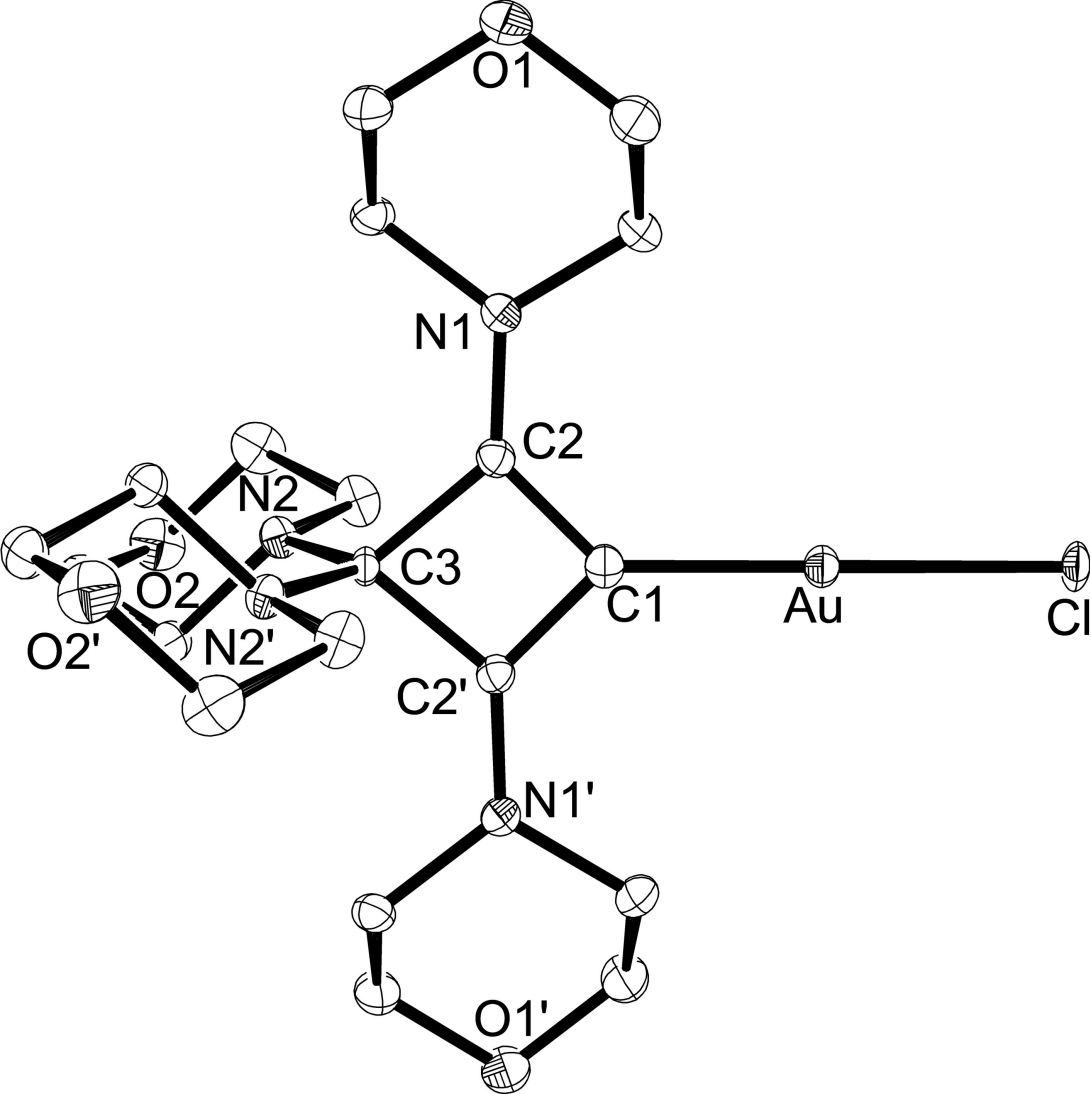
Molecular structure of **5** with thermal displacement parameters drawn at 50 % probability; hydrogen atoms are omitted for clarity. Selected bond lengths and angles are given in Table 1.

The ability of enyne **4** to form cyclic bent allene (CBA) complexes in the presence of transition metals was investigated by treatment with (THT)AuCl (THT=tetrahydrothiophene) in THF solution. The initial suspension became clear, followed by precipitation of the gold(I) complex **5**, which was isolated as a colorless solid in 82 % yield by filtration (Scheme 2). The formation of a cyclic CBA ligand was confirmed by ^13^C NMR spectroscopy with three signals for the ring carbon atoms at 178.1 (C2/C4), 127.3 (C1), and 95.6 ppm (C3). It is noteworthy that the signal for the metal‐bound carbon atom C1 is found at significantly higher field compared to conventional NHC‐gold complexes such as [(IMes)AuCl] (173.4 ppm, IMes=1,3‐bis(2,4,6‐trimethylphenyl)imidazolin‐2‐ylidene) and [(SIMes)AuCl] (195.0 ppm, SIMes=1,3‐bis(2,4,6‐trimethylphenyl)imidazolidin‐2‐ylidene).^[33]^ X‐ray diffraction analysis provided the molecular structure of **5** (Figure 4). Since the molecule resides on a crystallographic *C*_2_ axis passing through the atoms Cl−Au−C1−C3, the gold coordination sphere is perfectly linear (C1−Au−Cl=180°), and the four‐membered ring as well as the atoms Au, Cl, N1 and N1’ are coplanar to within 0.005 Å. At 1.985(2) Å, the Au−C1 bond length is very similar to those established for NHC gold(I) complexes, compare 1.998(5) Å in [IMes)AuCl] and 1.983(4) Å in [(SIMes)AuCl].^[33]^ Noteworthy, the possibilty to introduce more than one CBA ligand was revealed by isolation of single crystals of the corresponding homoleptic gold(I) complex [(CBA)_2_Au]Cl (**S2**) on one occasion from dichloromethane solution (see the Supporting Information for the crystal structure of **S2** ⋅ 1.5CH_2_Cl_2_). The formation of this by‐product could also account for the slightly reduced yields observed upon metal complexation.

**Figure 5:**
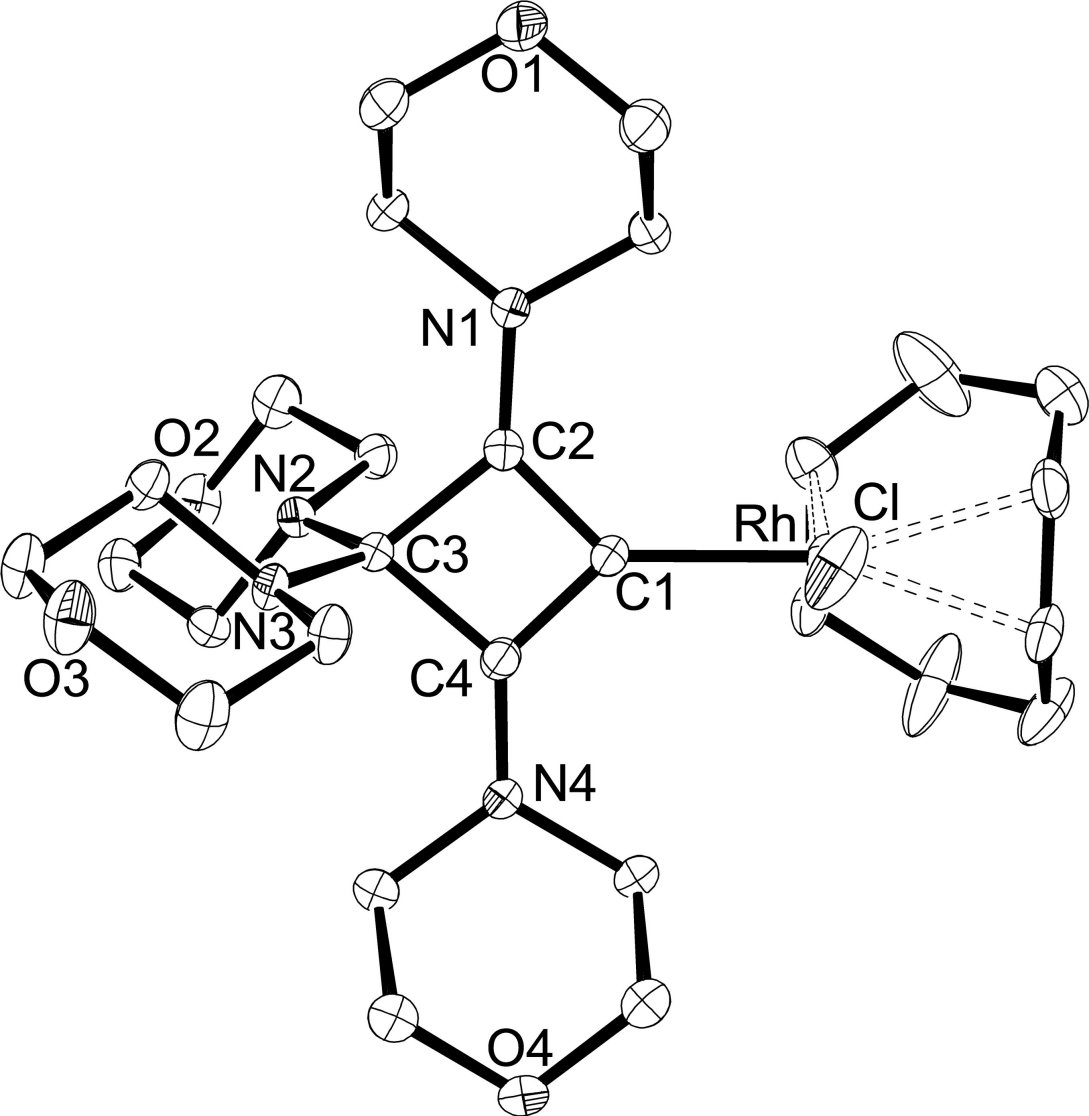
Molecular structure of **6** with thermal displacement parameters drawn at 50 % probability; hydrogen atoms are omitted for clarity. Selected bond lengths and angles are given in Table 1.

In a similar fashion, the rhodium(I) CBA complex **6** was isolated as a yellow solid in 56 % yield from the reaction of enyne **4** with half an equivalent of [Rh(COD)(μ‐Cl)]_2_ in THF solution. Bubbling CO through a THF solution of **6** afforded the *cis‐*dicarbonyl complex **7** as a dark yellow solid in 62 % yield (Scheme 2). The carbon atoms of the four‐membered CBA ligand in **6**/**7** gave rise to ^13^C NMR signals at 179.1/180.2 (C2/C4), 148.6/135.8 (C1), and 95.7/94.6 ppm (C3), with the metal bound carbon atoms (C2) producing dubletts with ^1^ *J*_C‐Rh_ coupling constants of 41/32 Hz (**6**/**7**). These values are in good agreement with those reported for similar bis(piperidinyl) complexes.[^11^, ^15^] Likewise, the CO stretching frequencies determined for **7** of 1984 and 2064 cm^−1^ ($\tilde{\upsilon }$_av_=2024 cm^−1^) confirm the strong donor ability of the CBA ligand. The solid‐state structures of **6** and **7** could be established by X‐ray diffraction analysis, and pertinent structural parameters are assembled in Table 1. The molecular structure of **6** is shown in Figure 5, while that of **7** is presented in the Supporting Information (Figure S5). In both structures, the rhodium atoms display the expected square‐planar coordination spheres, with the four‐membered CBA ligands adopting perpendicular orientations with dihedral angles of 83.9(1)° (**6**) and 85.2(2)° (**7**) between the C1−C2−C3−C4 and C1−Rh−Cl planes. The Rh−C1 distances are 2.012(2) Å (**6**) and 2.053(4) Å (**7**) and fall within the range established for the corresponding bis(piperidinyl) systems.[^11^, ^15^]

**Table 1:** Selected bond lengths and angles of the CBA complexes **5**, **6**, and **7**.

Bond lengths [Å]	**5**	**6**	**7**
C1−M	1.985(2)	2.012(2)	2.053(4)
C1−C2	1.409(2)	1.412(3)	1.400(6)
C2−C3	1.556(2)	1.548(3)	1.538(5)
C3−C4	1.556(2)	1.547(3)	1.5557(5)
C1−C4	1.409(2)	1.415(3)	1.409(6)
C2−N1	1.321(2)	1.327(3)	1.320(5)
C3−N2	1.457(2)	1.456(3)	1.455(5)

Bond angles [°]			
C1−M−Cl	180.00	88.4(7)	86.4(1)
C1−M−E^[a]^	–	90.2(7)	87.3(2)
C2−C1−C4	90.19(18)	89.1(2)	89.8(3)
C1−C2−C3	95.01(12)	95.7(2)	95.8(3)
C2−C3−C4	79.79(14)	79.7(2)	79.7(3)
M−C1−C3	180.00	178.3(2)	176.4(3)

[a] E=centroid_C25‐C26_ (**6**), C22≡O (**7**).

**Scheme 3:**
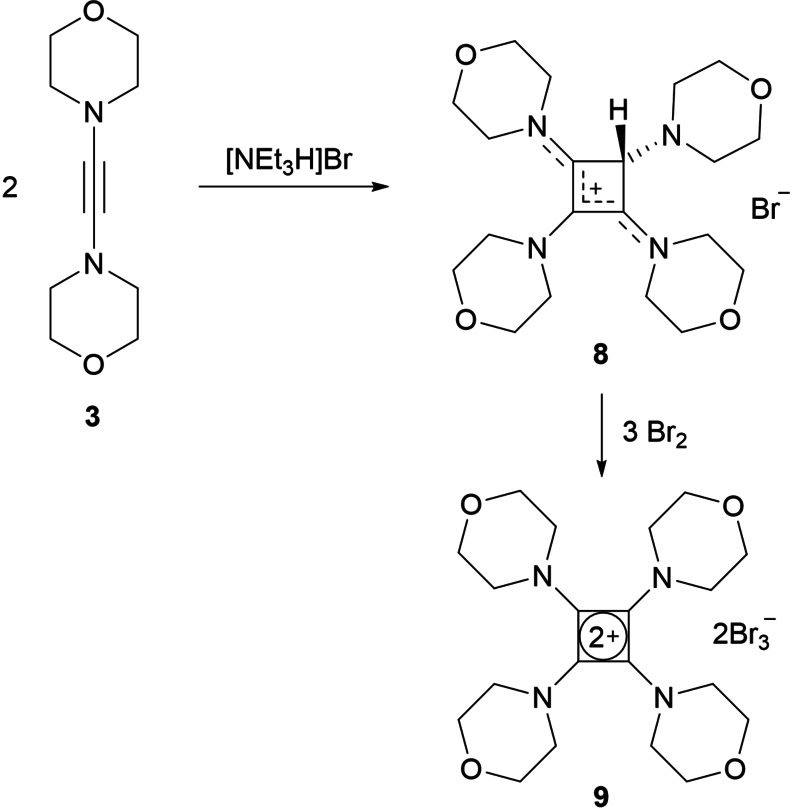
Synthesis of mono‐ and dicationic tetraaminocyclobutadiene species.

**Figure 6:**
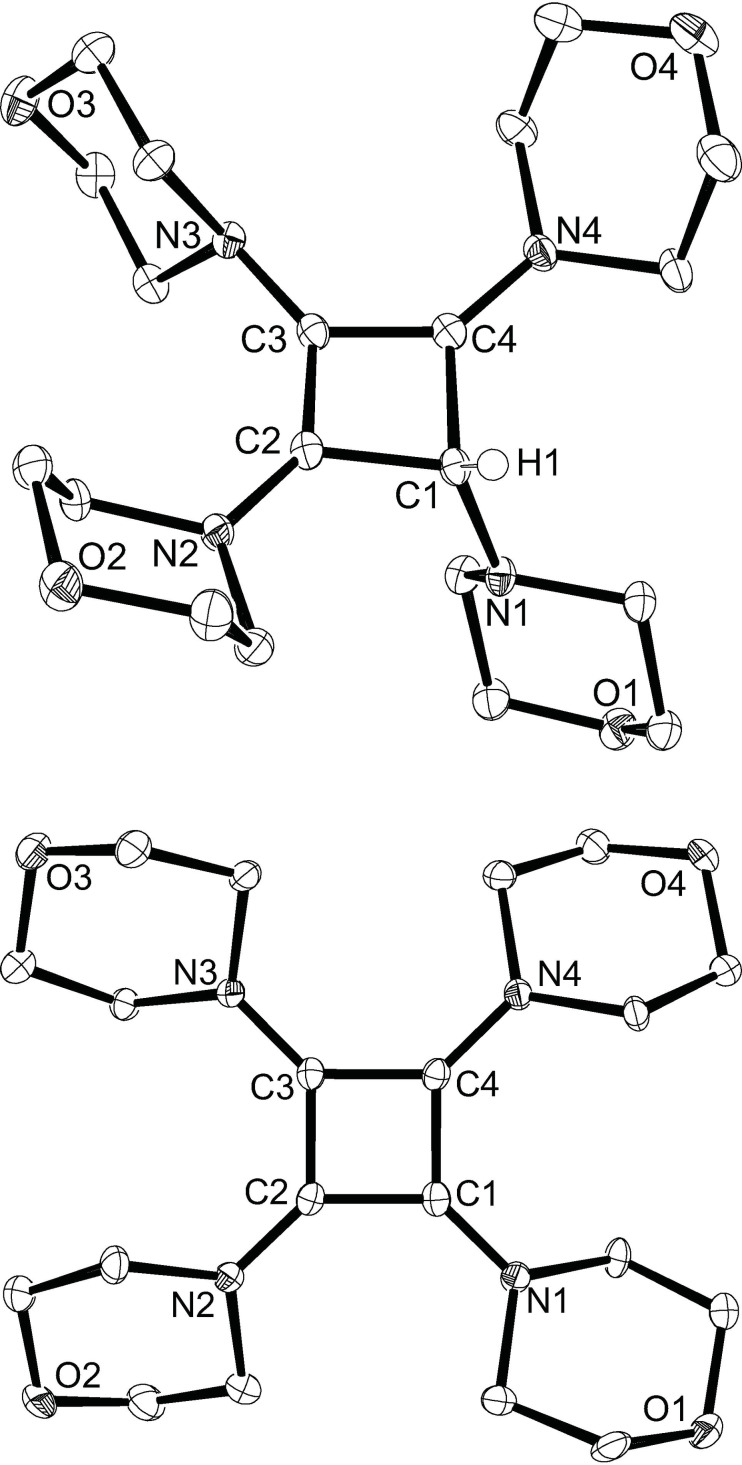
Molecular structures of the cations in **8** and **9** with thermal displacement parameters drawn at 50 % probability; hydrogen atoms (except for H1 in **8**) and anions (Br^−^ in **8** and 2xBr_3_^−^ in **9**) are omitted for clarity. Pertinent structural data is assembled in Table 2.

In a similar fashion as described by Viehe and coworkers for the preparation of tetrakis(dimethylamino)cyclobutenediylium dibromide (**VI**, Figure 1) in two steps from bis(dimethylamino)acetylene,^[17]^ the reaction of dimorpholinoacetylene (**3**) with triethylammonium bromide in dichloromethane afforded the cyclobutenylium bromide **8** as a beige solid in 68 % yield after evaporation and washing with ethyl acetate (Scheme 3). In the ^1^H NMR spectrum, a singlet at 6.04 ppm can be assigned to the hydrogen atom at the ring carbon atom C1, which is at lower field compared to 5.10 ppm reported for the related tetrakis(dimethylamino)cyclobutenylium bromide.^[17]^ While no ^13^C NMR data were reported for the latter compound, **8** gives rise to ^13^C NMR signals at 168.8 (C2/C4), 117.7 (C3), and 67.4 ppm (C1, this signal was identified in the ^1^H‐^13^C‐HSQC 2D NMR by a correlation peak to the signal at 6.04 ppm). These values are in good agreement with those of the structurally related Sn and Ge complexes **II**,[^10^, ^11^] indicating cyclization and formation of a four‐membered ring upon protonation of DAA **3**. X‐ray diffraction analysis provided additional evidence for the formation of a cyclobutenylium ion, and the molecular structure of **8** reveals a nearly planar C_4_ ring that is only slightly puckered with a dihedral angle of 6.1(2)° between the C2−C3−C4 and C2−C1−C4 planes (Figure 6, top). Planarization of the nitrogen atoms N2 and N4 with angle sums of 356.7° (N2) and 359.1° (N4) and short N2−C2 and N4−C4 bond lengths of 1.318(2) and 1.303(2) Å indicate π‐delocalization of the positive charge over the N2−C2−C3−C4−N4 cyanine‐type moiety, whereas the morpholino rings at C1 and C3 adopt perpendicular orientations with significantly smaller angles sums of 339.4° (N1) and 343.7° (N3). The bromide counterion is well separated from the cyclobutenylium cation and lies above the C_4_ plane with 3.603(2) Å from the centroid of the four‐membered ring and has short contacts with the CH_2_ hydrogen atoms of neighboring morpholine rings.

Addition of one equivalent of bromine to a solution of **8** in dichloromethane was expected to afford the tetra(morpholino)cyclobutenediylium dication as a dibromide salt, however, mixtures of bromide and presumably polybromide salts were obtained under various reaction conditions.^[34]^ In contrast, the reaction of **8** with an excess of bromine (3 equiv.) at 0 °C led to the precipiation of the bis(tribromide) salt **9** as a yellow powder from the dichloromethane reaction solution. Careful removal of the supernatant solution and washing with dichloromethane afforded **9** in 38 % yield. This comparatively low yield can tentatively be ascribed to partial hydrolysis of **8** and/or **9** under these reaction conditions since crystals of the monocationic oxygenated side products **S3** and **S4** were isolated from the separated dichloromethane solution (see the Supporting Information for X‐ray crystallographic details). ^1^H and ^13^C{H} NMR spectra of **9** were recorded in CD_3_CN solution, revealing the presence of four equivalent morpholino substituents as expected for a symmetric cyclobutadiene dication. Accordingly, only one lowfield ^13^C NMR signal at 148.3 ppm was found, which can be assigned to the quarternary ring carbon atoms. This chemical shift agrees well with the value of 152.8 ppm reported for hexakis(dimethylamino)benzene.^[35]^

Single crystals of **9** were obtained by layering a CH_3_CN solution on EtOAc at −40 °C, and X‐ray diffraction analysis confirmed the formation of a cyclobutadiene dication with two tribromide counterions. The C_4_N_4_ unit is almost perfectly planar, and the carbon atoms C1−C4 are coplanar to within 0.009 Å, with a maximum deviation of 0.044 Å of the nitrogen atoms from this plane. The atoms N1−N4 exhibit trigonal‐planar environments with angle sums close to 360° and short carbon‐nitrogen bond lengths of 1.312(7)–1.327(7) Å, which reveals significant π‐interaction; however, the morpholino units are slightly twisted by about 30°, presumably for steric reasons. The carbon‐carbon bond lengths in the four‐membered ring range from 1.450(8) to 1.462(6) Å, falling between the typical values of 1.54 and 1.34 Å assigned to carbon‐carbon single and double bonds, respectively. Similar values are also commonly found in compounds containing the squarate dianion (C_4_O_4_^2−^).^[36]^ Finally, the tribromide counterions exhibit strictly linear geometries, i. e. Br1−Br2−Br3=178.29(3)° and Br4−Br5−Br6=179.11(3)°, with typical Br−Br distances of Br1−Br2=2.5764(9) Å, Br2−Br3=2.5284(9) Å, Br4−Br5=2.5535(9) Å, Br5−Br6=2.5534(9) Å.^[34]^ In the crystal structure, the cations and anions form alternating layers with each Br_3_^−^ ion making short contacts of approximately 3.5 Å to the C_4_ ring of one dication, which is just slightly below the sum of the crystallographic van der Waals radii (3.6 Å).^[37]^ A packing diagram is provided in the Supporting Information (Figure S8).

### Computational study of cyclobutadiene dications

**Scheme 4:**
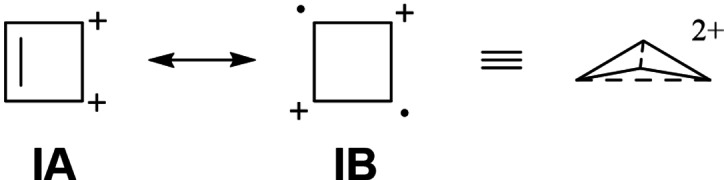
Resonance structures and 1,3‐interactions of folded C_4_H_4_^2+^.

According to Hückel's 4n+2‐rule,^[38]^ cyclobutadiene dications should be aromatic systems, as they fulfil this rule with n=0, which would result in a planar C_4_X_4_^2+^ unit. However, this planarity has never been demonstrated experimentally for the dicationic systems C_4_H_4_^2+^ and C_4_Me_4_^2+^, and theoretical calculations yielded nonplanar ground‐state structures with *D*_2d_ instead of *D*_4h_ symmetry.[^21^, ^22^, ^23^, ^24^, ^25^] The non‐planarity of the system can be explained by considering the resonance structures of the parent cyclobutadiene dication. Thus, there are four structures of type **IA** in which the two positive charges are located at adjacent carbon atoms, rendering these structures unstable due to charge repulsion. The two resonance forms of type **IB** have the charges further apart but are diradicals. To stabilize the diradical, the system folds to form partial bonds between the carbon atoms in the 1,3‐ and 2,4‐positions, respectively (Scheme 4). Note that despite the non‐planarity, the two π‐electrons are fully delocalized over the four carbon atoms, resulting in an aromatic system. This also explains why the planar cyclobutadiene dication (a transition state between the two non‐planar structures) is less aromatic than the non‐planar structure since the destruction of the 1,3‐interactions reduces the delocalization (see below).

It was also predicted that the quest for planar C_4_X_4_^2+^ systems will depend on the substituents X and their π‐donating ability.^[21]^ With the available structure of the tetramorpholinocyclobutadiene dication, i. e. C_4_(Morph)_4_^2+^ in **9**, we set out to evaluate its aromaticity and that of the related species C_4_(NMe_2_)_4_^2+^ and C_4_(NH_2_)_4_^2+^ together with C_4_H_4_^2+^ and C_4_Me_4_^2+^ by quantum chemical methods. Thus, the structures of these species were optimized and characterized as local minima at the D3‐B3LYP/6‐311++G(d,p) level of theory. Surprisingly, geometry optimization of C_4_(Morph)_4_^2+^ afforded two stable isomers **a** and **b** with almost equal energy (Δ*E*_0_=2.09 kcal mol^−1^). The energetically slightly favored isomer **a** is planar with a marginal C1−C2−C3−C4 torsion angle of 0.7° and structurally almost identical to the experimentally determined structure of the dication in **9** (Table 2). In contrast, isomer **b** is significantly folded and exhibits a dihedral angle of 17.1°. The structural parameters of the C_4_(NMe_2_)_4_^2+^ system are very similar to those in isomer **a** with twisted orientations of the amino substituents and a planar C_4_ ring. In contrast, the parent amino system C_4_(NH_2_)_4_^2+^ exhibits consistently shorter bonds and a perfectly planar *D*_4h_ geometry, in agreement with semi‐empirical calculations.^[29]^ For the dications C_4_Me_4_^2+^ and C_4_H_4_^2+^, puckered structures with dihedral angles of 20.2° and 30.5° were obtained, in agreement with previous calculations.^[26]^

**Table 2:** Endo‐ and exocyclic bond lengths, ring torsion angles of **8**, **9**, and of calculated cyclobutadiene dications.^[a]^

Bond lengths [Å]	**8** (exp)^[b]^	C_4_(Morph)_4_^2+^ in **9** (exp)^[b]^	C_4_(Morph)_4_^2+^ Isomer **a** (calc)^[b]^	C_4_(Morph)_4_^2+^ Isomer **b** (calc)^[b]^	C_4_(NMe_2_)_4_^2+^ (calc)^[b]^	C_4_(NH_2_)_4_^2+^ (calc)^[b]^	C_4_Me_4_^2+^ (calc)^[c]^	C_4_H_4_^2+^ (calc)^[d]^
C1−C2	1.526(2)	1.454(7)	1.467	1.480	1.464	1.447	1.445	1.426
C2−C3	1.407(2)	1.449(8)	1.466	1.460	1.464	1.447	1.445	1.426
C3−C4	1.421(2)	1.462(6)	1.467	1.471	1.464	1.447	1.445	1.426
C4−C1	1.536(2)	1.453(8)	1.466	1.455	1.464	1.447	1.445	1.426
C1−X1	1.449(2)	1.321(7)	1.320	1.328	1.320	1.310	1.452	1.098
C2−X2	1.318(2)	1.327(7)	1.320	1.322	1.320	1.310	1.452	1.098
C3−X3	1.410(2)	1.312(7)	1.320	1.312	1.320	1.310	1.452	1.098
C4−X4	1.303(2)	1.320(7)	1.320	1.314	1.320	1.310	1.452	1.098
Dihedral angle C1−C2−C3−C4 [°]								
	4.6(1)	1.4(4)	0.7	17.1	0.0	0.0	20.2	30.5

[a] D3‐B3LYP/6‐311++G*. [b] X=N. [c] X=C_Me_. [d] X=H.

When assessing aromaticity, one must be aware that substituents, especially π donors (and π acceptors) are not electronically benign.^[39]^ Taking C_4_(NH_2_)_4_^2+^ as an example: The system is completely planar, so there is a perfect conjugation between all p_π_ orbitals – four on the carbon atoms and four on the nitrogen atoms. The number of π electrons is 10, delocalized over the entire π system. Looking only at the four‐membered ring gives an incomplete picture. Even using the fragment molecular orbital (FMO) approach, it is clear that π donors/acceptors change the electron density at the ring and its aromatic properties. In the case discussed here, electron donors cause the *cyclo*‐C_4_ system to contain more than 2 electrons, reducing its aromaticity by moving towards a 4π‐electron (antiaromatic) system. Accordingly, two contradictory stabilizing effects must be considered for the dications C_4_(NR_2_)_4_^2+^: on the one hand, aromaticity, which is maximal when two electrons are delocalized, and on the other hand, the positive charge, which is further delocalized and stabilized when electron density is transferred from the substituents to the ring. In fact, the NBO charges indicate almost equal localization of the two positive charges on the C_4_ ring and on the four amino substituents, for example, +1.08 and +0.92 for C_4_(NR_2_)_4_^2+^ (R=H, Me; see the Supporting Information). In this regard, the two isomers **a** and **b** of the dication C_4_(Morph)_4_^2+^ might be regarded as a manifestation of this “dilemma” between optimizing charge stabilization and aromaticity in planar (**a**) and folded (**b**) systems, respectively.

The Nucleus Independent Chemical Shift (NICS) method has become the most widely used computational method for the identification and quantification of aromaticity.^[40]^ There are several NICS‐based methods, all are based on the idea of a ghost probe (no nuclei, no orbitals, no electrons) placed anywhere, and its absolute shielding, which is a manifestation of the induced magnetic field, is computed, usually by the Gauge‐Independent Atomic Orbital (GIAO) method.^[41]^ The most common NICS‐based method is NICS(1)_zz_, where the probe is placed 1 Å above the centre of the molecular plane and only the out‐of‐plane component ZZ (assuming that the system is in the XY plane) of the shielding tensor is considered. The most refined NICS‐based method, however, is based on distilling the contribution of only the π molecular orbitals to the out‐of‐plane part of the shielding tensor (NICS_π,zz_) at 1 or 1.7 Å above the molecular plane, or as an integral of NICS values from the molecular plane to infinity (∫NICS_π,zz_).^[42]^

**Figure 7:**
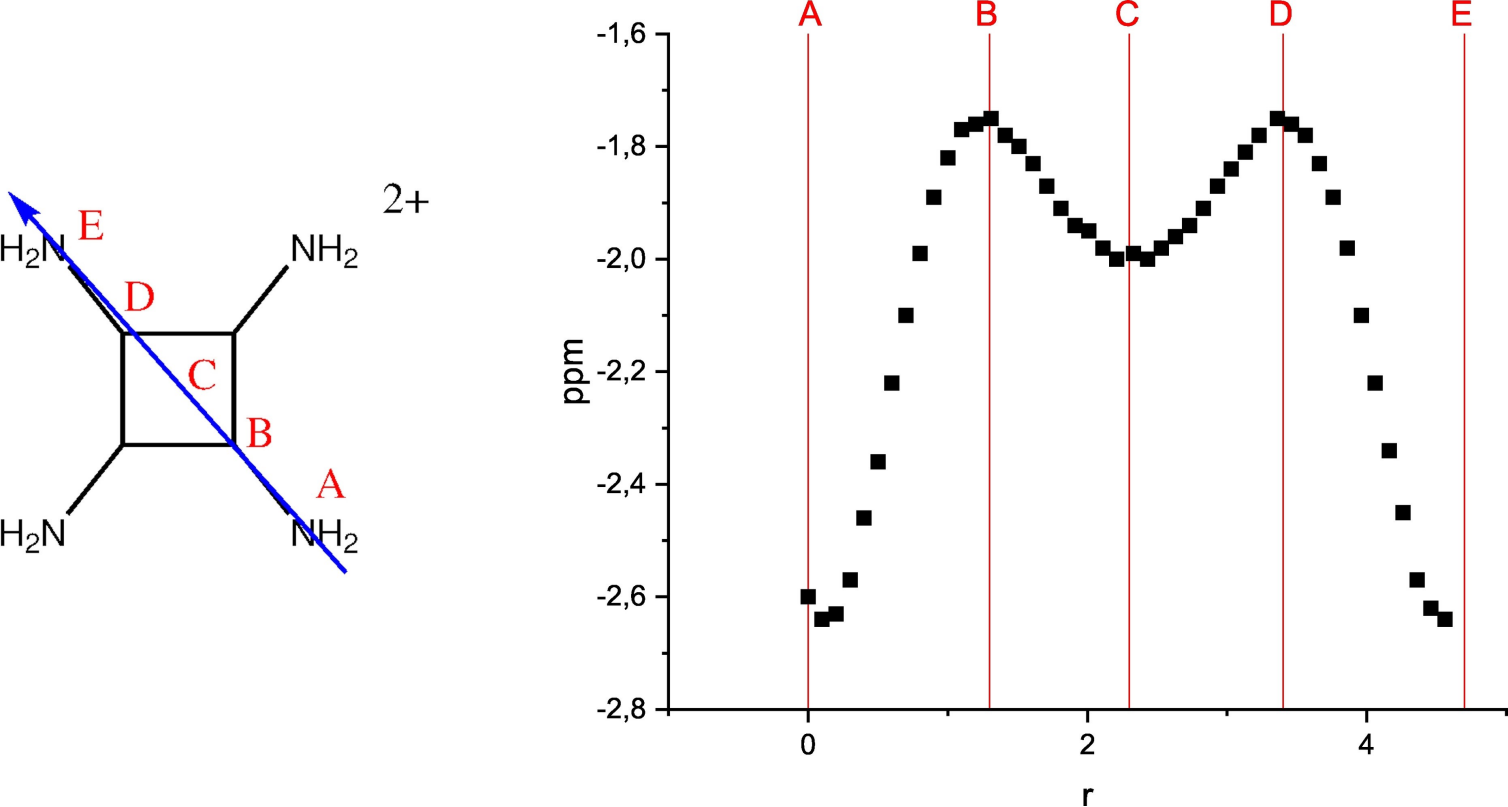
CMO‐NICS(1.7) _π,zz_ scan of C_4_(NH_2_)_4_^2+^.

Table 3 summarizes the NICS(1)_π,zz_ of the different C_4_X_4_^2+^ derivatives. While the parent cyclobutadiene dication is as aromatic as benzene (Entry 1), it loses a large part of its diatropicity upon planarization (see above, Entry 2). The tetramethyl‐substituted system shows ca. 50 % decrease in diatropicity, suggesting a rather effective electron donation from the Me groups into the ring (Entry 3). The relatively short C_ring_−C_Me_ bond length (1.452 Å), the small H−C_Me_−C_ring_ angle of the hydrogen perpendicular to the ring (105.6°) and the longer C−H bond of this hydrogen atom (1.111 Å vs. 1.092 Å for the other two C−H bonds) suggests efficient hyperconjugation. As in the parent dication, about 50 % of the tropicity is lost on planarization (Entry 4). Since conjugation is more efficient than hyperconjugation, more electron density is transferred from the amino (NR_2_) compared to methyl (Me) groups. This is reflected in a further reduction of the NICS(1)_π,zz_ values in the planar C_4_(NR_2_)^2+^ (R=H, Me) systems to values that may be considered as non‐aromatic (Entries 5 and 6). Indeed, the NICS‐XY scan of C_4_(NH_2_)_4_^2+^ (Figure 7) shows that the major diatropicity is located on the nitrogen atoms, while the ring current is minimal (and diatropic) at the four‐membered ring. As mentioned above, the competing effects, charge stabilization and aromaticity, are manifested in the two isomers of C_4_(Morph)_4_^2+^: **a**, which is almost planar and therefore non‐aromatic (Entry 7) and **b**, which is folded and exhibits slightly higher diatropicity, namely, the C_4_ ring is about 19 % aromatic with respect to the parent C_4_H_4_^2+^ system (Entry 8). The electron donation to the four‐membered ring and the diatropic currents at the nitrogen atoms are also experimentally evidenced by a ca. 0.9 ppm downfield shift of the NCH_2_ hydrogen atoms in **9** relative to **3** (see the Experimental Section). Apparently, at least 50 % of the delocalization (aromaticity) at the 4‐membered ring in all C_4_R_4_^2+^ derivatives result from the 1,3‐interactions (Scheme 4) since these are lost upon planarization.

**Table 3:** σ‐only‐NICS(1)_π,zz_ of some dicationic cyclobutadiene derivatives; CMO‐NICS(1)_π,zz_ (available only for completely planar systems) are given in parentheses.

Entry		NICS(1)_π,zz_
1	C_4_H_4_^2+^	−35.4
2	C_4_H_4_^2+^, planar	−14.7 (−11.6)
3	C_4_Me_4_^2+^	−16.6
4	C_4_Me_4_^2+^, planar	−8.9 (−7.8)
5	C_4_(NH_2_)_4_^2+^	−4.7 (−3.0)
6	C_4_(NMe_2_)_4_^2+^	−2.3
7	C_4_(Morph)_4_^2+^ (isomer **a**)	−2.4
8	C_4_(Morph)_4_^2+^ (isomer **b**)	−6.9

**Figure 8:**
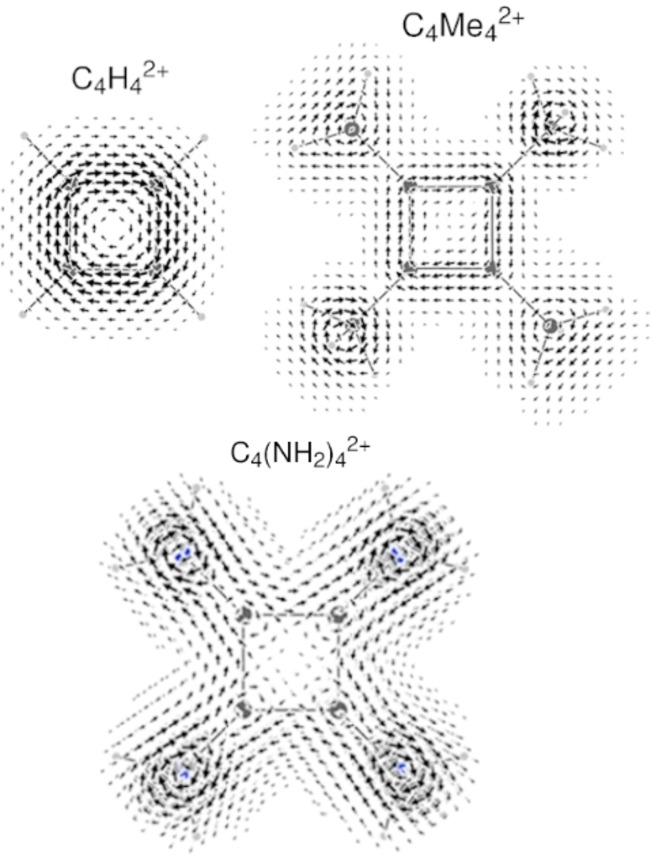
π‐Current density of planar C_4_H_4_^2+^, planar C_4_Me_4_^2+^ and C_4_(NH_2_)_4_^2+^.

To obtain a clean π contribution based on orbital analyses, the π system must be orthogonal to the σ system, which is possible only in planar systems. Thus, Figure 8 shows the current density of planar C_4_H_4_^2+^, C_4_Me_4_^2+^, and C_4_(NH_2_)_4_^2+^. Although the tropicity of planar C_4_H_4_^2+^ and C_4_Me_4_^2+^ are reduced with respect to the optimized folded structures, the trend is very clear: The parent C_4_H_4_^2+^ shows a diatropic circuit at the ring. The C_4_Me_4_^2+^ shows local diatropic circuits on the Me groups, a global diatropic current and a local current at the four‐membered ring, which is significantly reduced relative to the parent system. This trend, which is also reflected in the NICS(1)_π,zz_ values (Table 3), continues with C_4_(NH_2_)_4_^2+^, exhibiting a global current, local currents at the NH_2_ groups and only a negligible local current at the four‐membered ring, in accordance with the NICS‐XY scan (Figure 7).

## Conclusion

With the synthesis and characterization of dimorpholinoacetylene (**3**) an important new derivative of this interesting class of electron‐rich diamminoacetylenes (DAA) has been established, and the tendency of these species to undergo dimerization has been confirmed by isolation and structural characterization of the enyne 1,3,4,4‐tetramorpholinobut‐1‐yn‐3‐ene (**4**). The reaction of the latter with transition metals provided access to complexes containing four‐membered cyclic bent allene (CBA) ligands, which represent an interesting new class of potential ancillary ligands for applications in homogeneous catalysis. Furthermore, the reaction of DAA **1** with triethylammonium bromide followed by oxidation with bromine afforded tetra(morpholino)cyclobutenediylium bis(tribromide) (**9**). X‐ray diffraction analysis provided the molecular stucture of a tetraaminocyclobutadiene dication, namely C_4_(Morph)_4_^2+^ in **9**, with an almost perfectly planar C_4_ ring, which agrees with the ground‐state geometries predicted for this and related tetraaminocyclobutadiene dications C_4_(NR_2_)_4_^2+^ (R=Me, H) by density functional theory (DFT) calculations, in contrast to the puckered geometry of the parent C_4_H_4_^2+^ system. Detailed studies employing advanced NICS methods were conducted to assess the issue of aromaticity for these species, which reveal that the π‐donating ability of the amino substituents is responsible for the observed planarity and for the delocalization of the positive charge over the nitrogen atoms, which perturbs the aromatic ring current. It is important to realize that ‐ counterintuitively ‐ the cyclobutadiene dication derivatives are more aromatic when they are puckered rather than planar. The presence of π‐donating amino substituents, however, leads to planarization at the expense of aromaticity. In principle, this conflict manifests itself in the two computationally derived, almost isoenergetic isomers of C_4_(Morph)_4_^2+^, of which the planar isomer **a**, as structurally authenticated in **9**, can be described as non‐aromatic, while the puckered isomer **b** can be considered slightly aromatic with a ring current about 20 % of that in benzene.^[39]^

## Experimental Section

**Material and Methods**: Unless otherwise noted, all reactions have been performed in dry argon atmosphere in a Glove Box (MBraun 200B) or using a high vacuum line with common Schlenk techniques. Elevated temperatures were achieved by a silicone oil bath, whereas low temperatures were provided by a sodium chloride/ice bath (0 °C). Starting materials were purchased from commercial sources (TCI, abcr, Merck, Roth, Alfa‐Aesar) and if necessary purified by conventional techniques or dried over CaH_2_. Solvents were dried either over Na/benzophenone, CaH_2_ (chlorinated solvents) and distilled or by a SPS (solvent purification system) with subsequent degassing (“freeze‐pump” or ultrasonic bath). They were stored in dry argon atmosphere over molecular sieves (4 Å). NMR spectra were recorded on Bruker AVII300, AVIIHD300 (300 MHz) or AVII500 (500 MHz) and referenced to the residual undeuterated solvent signal (^1^H NMR) or the solvent signal itself (^13^C{^1^H} NMR), respectively. Coupling constants (*J*) are indicated in Hertz (Hz) and splitting patterns are specified as multiplett (m), singlet (s), doublet (d) or triplet (t). Figures of the NMR spectra can be found in the Supporting Information. Elemental analyses were performed on a Vario Micro Cube System. IR spectra were measured on a Bruker Vertex 70 with a spectral resolution of 1 cm^−1^ in a KCl glass cuvette and DCM as solvent. For crystallographic details, see the Supporting Information.

Deposition Number(s) 2159699, 2159700, 2159701, 2159702, 2159703, 2159704, 2159705, 2159706, 2159707, 2159708, 2159709 contain(s) the supplementary crystallographic data for this paper. These data are provided free of charge by the joint Cambridge Crystallographic Data Centre and Fachinformationszentrum Karlsruhe Access Structures service.

### Synthetic procedures

**1,1‐Dimorpholinoethene (1)**: In a vacuum distillation apparatus, N,N‐dimethylacetamide dimethyl acetal (10.3 g, 77.3 mmol, 1 equiv.) was treated with morpholine (20 mL, 231.9 mmol, 3 equiv.) and heated in a pre‐heated oil bath to 110 °C for 1 h, while methanol destilled off. The temperature was then raised to 150 °C and stirred overnight. Thereafter, the excess of morpholine and all remaining volatile components were removed in vacuo (50 mbar, 150 °C, 30 min). The residue was cooled to rt, leading to crystallization. The product was dried under high vacuum and isolated as an orange solid (12.32 g, 62.1 mmol, 80 %). ^**1**^ **H NMR** (300 MHz; CDCl_3_): *δ* (ppm)=3.68 (t, ^3^ *J*_H,H_=4.6 Hz, 8H, O−CH_2_), 3.30 (s, 2H, =CH_2_), 2.86 (t, ^3^ *J*_H,H_=4.6 Hz, 8H, N−CH_2_). ^**13**^ **C{^1^H} NMR** (75 MHz; CDCl_3_): *δ* (ppm)=162.3 ((N)_2_C=), 70.4 (CH_2_=), 67.2 (O−CH_2_), 49.9 (N−CH_2_). **EA** – Anal. calc. for C_10_H_18_N_2_O_2_: C 60.58; H 9.15; N 14.13; found: C 60.79, H 9.32, N 14.22.

**1,1‐Dibromo‐2,2‐dimorpholinoethene (2)**: 1,1‐Dimorpholinoethene (3.8 g, 19.2 mmol, 1 equiv.) und triethylamine (6.37 mL, 46.0 mmol, 2.4 equiv.) were dissolved in DCM and bromine (2.01 mL in 15 mL DCM, 39.3 mmol, 2.05 equiv.) was added drop by drop over a period of 15 min at 0 °C. The mixture was stirred further 10 min at that temperature and the solvent was removed in vacuo. The product was extracted with toluene (20+2×15 mL) quickly in air and has been obtained after removal of the solvent and drying in high vacuum as a light‐yellow solid (5.578 g, 15.7 mmol, 82 %). ^**1**^ **H NMR** (300 MHz; C_6_D_6_): *δ* (ppm)=3.31 (t, ^3^ *J*_H,H_=4.6 Hz, 8H, O−CH_2_), 2.71 (t, ^3^ *J*_H,H_=4.6 Hz, 8H, N−CH_2_). ^**13**^ **C{^1^H} NMR** (75 MHz; C_6_D_6_): *δ* (ppm)=153.6 ((N)_2_C=), 67.0 (O−CH_2_), 55.2 (CBr_2_), 49.5 (N−CH_2_). **EA** – Anal. Calc. for C_10_H_16_Br_2_N_2_O_2_: C 33.73; H 4.53; N 7.87; found: C 33.63, H 4.58, N 7.53.

**Dimorpholinoacetylene (3)**: 1,1‐Dibromo‐2,2‐dimorpholinoethene (1.0 g, 0.28 mmol, 1 equiv.) was dissolved in toluene (20 mL) and treated with 1,4‐dioxane (0.48 mL, 5.6 mmol, 2 equiv.). Then *n*BuLi (1.6 m in *n*‐hexane, 2.12 mL, 3.4 mmol, 1.2 equiv.) was added dropwise while cooling with a water bath. The resulting orange suspension was stirred for 30 min at rt. Thereafter, it was filtrated over Celite, and the solvent of the light‐yellow filtrate was removed in vacuo. The residue was purified by recrystallization from THF/*n*‐hexane solution (2 : 2 mL) at −40 °C to yield the product as a colorless solid (325 mg, 0.17 mmol, 60 %). ^**1**^ **H NMR** (300 MHz; C_6_D_6_): *δ* (ppm)=3.42–3.36 (m, 8H, O−CH_2_), 2.87–2.81 (m, 8H, N−CH_2_). ^**13**^ **C{^1^H} NMR** (75 MHz; C_6_D_6_): *δ* (ppm)=73.9 (C≡C), 66.4 (O−CH_2_), 54.4 (N‐CH_2_). **EA** – Anal. Calc. for C_10_H_16_N_2_O_2_: C 61.20; H 8.22; N 14.27; found: C 61.47, H 8.52, N 14.37.

**1,1,2,4‐Tetramorpholino‐1‐buten‐3‐yne (4)**: Dimorpholinoacetylene (**3**) was dissolved in toluene (2 mL) and stirred at 110 °C for 24 h, with the solution taking on an orange color. The solvent was removed in vacuo, and the sticky orange residue was dissolved in *n*‐hexane (10 mL). By renewed removal of the solvent, the product was quantitatively obtained as a beige powder. ^**1**^ **H NMR** (500 MHz; C_6_D_6_): *δ* (ppm)=3.70 (t, ^3^ *J*_H,H_=4.7 Hz, 4H, =C(NCH_2_CH_2_O)), 3.57 (t, ^3^ *J*_H,H_=4.7 Hz, 4H, =C(NCH_2_CH_2_O)_2_), 3.51 (t, ^3^ *J*_H,H_=4.7 Hz, 4H, =C(NCH_2_CH_2_O)_2_), 3.44–3.40 (m, 4H, ≡C(NCH_2_CH_2_O)), 3.12 (t, ^3^ *J*_H,H_=4.7 Hz, 4H, =C(NCH_2_CH_2_O)_2_), 2.95 (t, ^3^ *J*_H,H_=4.7 Hz, 4H, =C(NCH_2_CH_2_O)_2_), 2.83–2.80 (m, 4H, ≡C(NCH_2_CH_2_O)), 2.68–2.58 (m, 4H, =C(NCH_2_CH_2_O)). ^**13**^ **C{^1^H} NMR** (126 MHz; C_6_D_6_): *δ* (ppm)=155.4 (=C(N_2_)), 101.4 (C=C(N_2_)), 101.1 (N−C≡C), 67.7 and 67.6 (=C(NCH_2_ CH_2_O)_2_), 67.5 (=C(NCH_2_ CH_2_O)), 66.1 (≡C(NCH_2_ CH_2_O), 58.5 (N−C≡C), 53.7 (≡C(NCH_2_CH_2_O)), 53.5 (=C(NCH_2_CH_2_O)), 50.1 and 50.0 (=C(NCH_2_CH_2_O)_2_). **EA**: Anal. calc. for C_20_H_32_N_4_O_4_: C 61.20; H 8.22; N 14.27; found: C 61.20, H 8.30, N 14.14.

**(CBA)AuCl (5)**: Under the exclusion of light, 1,3,4,4‐tetramorpholinobut‐1‐yn‐3‐ene (46.5 mg, 0.12 mmol, 2 equiv.) was dissolved in THF (2 mL) and (THT)AuCl (18.8 mg, 0.06 mmol, 1 equiv., in 2 mL THF) was added. The resulting yellow suspension was stirred for 4 h at rt and filtered. The solid was washed with THF (2 mL) and dissolved in DCM (5 mL). After the removal of the solvent in vacuo, the product was obtained as a colorless powder (30 mg, 0.03 mmol, 82 %). ^**1**^ **H NMR** (500 MHz; CDCl_3_): *δ* (ppm)=4.48–4.40 and 3.82–3.78 (m, 8H, C2/C4−N(CH_2_CH_2_)_2_O), 3.76–3.63 (m, 16H, C1−4‐N(CH_2_CH_2_)_2_O), 2.66 (br s, 8H, C3−N(CH_2_CH_2_)_2_O). ^**13**^ **C{^1^H} NMR** (126 MHz; CDCl_3_): *δ* (ppm)=178.1 (2C, C2/C4), 127.3 (1C, C1), 95.6 (1C, C3), 67.3 (4C, C3−N(CH_2_ CH_2_)_2_O), 67.2 und 66.7 (4C, C2/C4‐N(CH_2_ CH_2_)_2_O), 50.8 und 47.4 (4C, C2/C4−N(CH_2_CH_2_)_2_O), 49.0 (4C, C3−N(CH_2_CH_2_)_2_O). **EA** – Anal. Calc. for C_20_H_32_AuClN_4_O_4_: C 38.44; H 5.16; N 8.97; found: C 38.05, H 5.08, N 8.78.

**[(CBA)Rh(COD)Cl] (6)**: [Rh(COD)Cl]_2_ (62.8 mg, 0.127 mmol, 1 equiv.) was dissolved in THF (5 mL) and 1,3,4,4‐tetramorpholinobut‐1‐yn‐3‐ene (100 mg in 2 mL THF, 0.254 mmol 2 equiv.) was added, with the solution turning orange and appearance of a precipitate. The reaction mixture was stirred overnight, and the solvent was removed in vacuo. The residue was washed with *n*‐hexane (2×1 mL) and dried under high vacuum to yield the product as a yellow solid (91 mg, 0.14 mmol, 56 %). ^**1**^ **H NMR** (500 MHz; CDCl_3_): *δ* (ppm)=5.39–5.24 (m, 2H, C2/C4−N(CH_2_CH_2_)_2_O), 5.10–4.96 (m, 2H, C2/C4−N(CH_2_CH_2_)_2_O), 4.82–4.73 (m, 2H, CH_COD_), 4.08–3.85 (m, 4H, C2/C4−N(CH_2_CH_2_)_2_O), 3.78–3.50 (m, 16H, C2/C4−N(CH_2_CH_2_)_2_O und C3−N(CH_2_CH_2_)_2_O), 3.08–2.98 (m. 2H, CH_COD_), 2.77–2.68 (m, 4H, C3−N(CH_2_CH_2_)_2_O), 2.55–2.43 (m, 4H, C3−N(CH_2_CH_2_)_2_O), 2.28–2.12 (m, 4H, (CH_2_)_COD_), 1.89–1.77 (m, 4H, (CH_2_)_COD_). ^**13**^ **C{^1^H} NMR** (126 MHz; CDCl_3_): *δ* (ppm)=179.1 (2C, C2/C4), 148.6 (d, ^1^ *J*_RhC_=41 Hz, 1C, C−Rh), 95.7 (d, ^1^J_RhC_=6 Hz, 2C, CH_COD_), 93.8 (d, ^3^ *J*_RhC_=3 Hz, 1C, C3), 67.7 und 67.6 (2×2C, C3−N(CH_2_ CH_2_)_2_O), 67.4 und 67.0 (2×2C, C2/C4−N(CH_2_ CH_2_)_2_O), 66.0 (d, ^1^J_RhC_=15 Hz, 2C, HC_COD_), 50.1 und 48.4 (2×2C, C2/C4−N(CH_2_CH_2_)_2_O), 49.2 und 49.0 (2×2C, C3−N(CH_2_CH_2_)_2_O), 33.0 und 29.3 (2×2C, (CH_2_)_COD_). **EA** ‐ Anal. Calc. for C_28_H_44_ClN_4_O_4_Rh: C 52.63; H 6.94; N 8.77; found: C 52.27; H 7.18; N 8.43.

**[(CBA)Rh(CO)_2_Cl)] (7)**: [(MorphCBA)Rh(COD)Cl] (17.5 mg, 0.027 mmol) was dissolved in THF (4 mL) and CO was purged for 10 min into the solution. The reaction mixture was stirred for 1 h under CO atmosphere, and the solvent was removed in vacuo. The residue was washed with *n*‐hexane, precipitated out of a THF/n‐hexane solution and dried under high vacuum to yield the product as a dark yellow solid (10 mg, 0.017 mmol, 62 %). ^**1**^ **H NMR** (300 MHz; CDCl_3_): *δ* (ppm)=4.94–4.81 (m, 2H, C2/C4−N(CH_2_CH_2_)_2_O), 4.50–4.38 (m, 2H, C2/C4−N(CH_2_CH_2_)_2_O), 4.04–3.50 (m, 20H, C2/C4−N(CH_2_CH_2_)_2_O und C3−N(CH_2_CH_2_)_2_O), 2.83–2.59 (m, 8H, C3−N(CH_2_CH_2_)_2_O). ^**13**^ **C{^1^H} NMR** (75 MHz; CDCl_3_): *δ* (ppm)=186.3 (d, ^1^J_RhC_=53 Hz, 1C, C≡O) 184.6 (d, ^1^J_RhC_=78 Hz, C≡O), 180.2 (2C, C2/C4), 135.8 (d, ^1^J_RhC_=32 Hz, 1C, C1), 94.6 (d, ^3^ *J*_RhC_=3 Hz, 1C, C3), 67.6, 67.4, 67.1 and 66.8 (8C, N(CH_2_CH_2_)_2_O), 50.5, 49.4, 49.0 and 48.9 (8C, N(CH_2_CH_2_)_2_O). **IR** (DCM): ${\tilde{\nu }}_{CO}$ (cm^−1^)=2063.7, 1983.7. **EA** – Anal. calc. for C_22_H_32_ClN_4_O_6_Rh: C 45.03, H 5.50, N 9.55; found: C 44.57, H 5.23, N 9.21.

**1,2,3,4‐Tetramorpholinocyclobutenylium bromide (8)**: Dimorpholinoacetylene (100 mg, 0.51 mmol, 2 equiv.) was dissolved in DCM (3 mL) and triethylammoniumbromide (46.4 mg in 2 mL DCM, 0.51 mmol, 1 equiv.) was slowly added. The dark yellow suspension was stirred for 1.5 h at rt, and the solvent was removed in vacuo. The yellow residue was washed with ethyl acetate (3×4 mL) and dried under high vacuum to yield the product as a beige solid (83 mg, 0.18 mmol, 68 %). ^**1**^ **H NMR** (500 MHz; CDCl_3_): *δ* (ppm)=6.04 (s, 1H, C1−H), 4.17–4.03 (m, 4H, C3−N(CH_2_CH_2_)_2_O), 4.01–3.82 (m, 8H, C2/C4−N(CH_2_CH_2_)_2_O), 3.77–3.62 (m, 12H, C2/C4−N(CH_2_CH_2_)_2_O und C1−N(CH_2_CH_2_)_2_O), 2.91 (t, ^3^ *J*_H,H_=4.6 Hz, 4H, C3−N(CH_2_CH_2_)_2_O), 2.82 (t, ^3^J_H,H_=4.6 Hz, 4H, C1−N(CH_2_CH_2_)_2_O). ^**13**^ **C{^1^H} NMR** (126 MHz; CDCl_3_): *δ* (ppm)=168.8 (C2/C4), 117.7 (C3), 67.5 (C3−N(CH_2_ CH_2_)_2_O), 67.4 (C1−H), 67.2 and 67.1 (C2/C4−N(CH_2_ CH_2_)_2_O), 66.2 (C1−N(CH_2_ CH_2_)_2_O), 51.2 (C3−N(CH_2_CH_2_)_2_O), 51.0 and 49.7 (C2/C4−N(CH_2_CH_2_)_2_O), 49.1 (C1−N(CH_2_CH_2_)_2_O). **EA** – Anal. calc. for C_10_H_16_N_2_O_2_: C 50.74; H 7.03; N 11.83; found: C 50.90, H 7.27, N 11.36.

**Tetramorpholinocyclobutenediylium bis(tribromide) (9)**: 1,2,3,4‐Tetramorpholinocyclobutenylium bromide (90 mg, 0.19 mmol, 1 equiv.) was dissolved in DCM (6 mL) and bromine (10 Vol % in DCM, 0.29 mL, 0.57 mmol, 3 equiv.) was slowly added at 0 °C and stirred for 1 h at this temperature, affording an orange precipitate. The mixture was warmed up to rt, and the supernatant orange solution was removed. The residue was washed with additional DCM (3×3 mL) and dried under vacuum. The product was obtained as a yellow powder (64 mg, 0.073 mmol, 39 %). ^**1**^ **H NMR** (300 MHz; CD_3_CN): *δ* (ppm)=3.94–3.87 (m, 16H, OCH_2_), 3.75–3.69 (m, 16H, NCH_2_). ^**13**^ **C{^1^H} NMR** (76 MHz; CD_3_CN): *δ* (ppm)=148.3 (4C, C_q_), 66.3 (8C, OCH_2_), 52.7 (8C, NCH_2_). **EA** ‐ Anal. calc. for C_20_H_32_Br_6_N_4_O_4_: C 27.55, H 3.70, N 6.43; found: C 27.44, H 3.64, N 6.21.

**Computational methods**: All the calculations were carried out using the Gaussian 09^[41]^ program. Geometry optimizations and NICS calculations were carried out at the D3‐B3LYP/6‐311++G(d,p) and GIAO‐B3LYP/6‐311+G(d) computational level, respectively. All the NICS calculations were carried out using the Aroma^[43]^ software. NICS_π,zz_ values are obtained from the σ‐only model^[44]^ and (for planar systems) from CMO‐NICS_π,zz_ using the NCS^[45]^ procedure within NBO6.^[46]^ Current density plots were produced with SYSMOIC.^[47]^

## Conflict of interest

The authors declare no conflict of interest.

1

## Supporting information

As a service to our authors and readers, this journal provides supporting information supplied by the authors. Such materials are peer reviewed and may be re‐organized for online delivery, but are not copy‐edited or typeset. Technical support issues arising from supporting information (other than missing files) should be addressed to the authors.

Supporting Information

## Data Availability

The data that support the findings of this study are available in the supplementary material of this article.
