# Supplementary material for: Dimorpholinoacetylene and Its Use for the Synthesis of Tetraaminocyclobutadiene Species
Source: Chemistry. 2022 Nov 3;28(65):e202202737. doi: 10.1002/chem.202202737 (PMC9828195; doi:10.1002/chem.202202737)
Supplement: Supplementary file 1 — Supporting Information [file CHEM-28-0-s001.pdf]

Chemistry–A European Journal

Supporting Information

Dimorpholinoacetylene and Its Use for the Synthesis of Tetraaminocyclobutadiene Species

Lukas Körner, Luong Phong Ho, Ralph Puchta, Amnon Stanger, and Matthias Tamm*

Supporting Information

Content

S1 Crystallographic Details 2
S1.1 Dimorpholinoacetylene (**3**) 3
S1.2 1,3,4,4-Tetramorpholinobut-1-yn-3-ene (**4**) 5
S1.3 (CBA)AuCl (**5**) 7
S1.4 (CBA)Rh(COD)Cl (**6**). 9
S1.5 (CBA)Rh$(CO)_2$Cl (**7**) 11
S1.6 1,2,3,4-Tetramorpholinocyclobutenylium bromide (**8**) 13
S1.7 Tetramorpholinocyclobutenediylium bis(tribromide) (**9**). 15
S1.8 1,1-Dibromo-2,2-dimorpholinoethyl bromide (**S1**·$CHCl_3$). 17
S1.11 [$(CBA)_2$Au]Cl (**S2**) 19
S1.9 1-Bromo-3-oxo-2,4-dimorpholinocyclobutenylium tribromide (**S3**) 21
S1.10 1-Oxotrimorpholinocyclobutenylium tribromide (**S4**) 23
S2 NMR Spectra 25
S2.1 1,1-Dimorpholinoethene (**1**) 25
S2.2 1,1-Dibromo-2,2-dimorpholinoethene (**2**) 27
S2.3 Dimorpholinoacetylene (**3**) 29
S2.4 1,3,4,4-Tetramorpholinobut-1-yn-3-ene (**4**) 31
S2.5 (CBA)AuCl (**5**) 33
S2.6 (CBA)Rh(COD)Cl (**6**) 35
S2.7 (CBA)Rh$(CO)_2$Cl (**7**) 37
S.2.8 1,2,3,4-Tetramorpholinocyclobutadiene-1-hydrobromide **(8)** 39
S.2.9 Tetramorpholinocyclobutenediylium bis(tribromide) (**9**). 41
S3 Additional computational charge analysis 43
S4 Coordinates of optimized structures 44
S5 References 54

S1 Crystallographic Details

Suitable single crystals were mounted on a hair or on a MiTiGen mount in perfluorinated inert oil. The intensity measurements were performed at 100 K on an Oxford Diffraction Nova A and a Rigaku XtaLAB Synergy S Single Source diffractometer using mirror-focussed CuKα radiation or on an Oxford Diffraction Eos using monochromated MoKα radiation. The diffractometer software CrysAlisPRO was employed.[1] Absorption corrections were based on multiscans. The structures were refined anisotropically on F2 using SHELXL-2017/1 or -2018/3.[2] Hydrogen atoms were included using a riding model or rigid methyl groups. Further details are given in Table S1-11.

Solvent content: Compound **S2** contains one half of a dichloromethane per asymmetric unit and is disordered over several positions.

Exceptions and special details: Compound **4** contains two molecules per asymmetric unit. Compound **5** is located on a special position. Compound **S3** contains two Br_3 anions per asymmetric unit, which are both located on a special position and therefore both are only half occupied. Compound **S2** is located on a special position. The disordered solvent molecules in **S2** were refined using appropriate restraints to improve stability of refinement, but the dimensions are not entirely satisfactory and should be interpreted with caution.

Complete data have been deposited with the Cambridge Crystallographic Data Centre under the CCDC numbers 2159699–2159709 for compounds **3**–**9** and **S1**–**S4**. These data can be obtained free of charge from http://www.ccdc.cam.ac.uk/.

S1.1 Dimorpholinoacetylene (**3**)

Compound	**3**
Identification code	2159699
Empirical formula	$C_{10}H_{16}N_2O_2$
Formula weight	196.25
Temperature	100(2) K
Wavelength	1.54184 Å
Instrument (scan mode)	XtaLAB Synergy, Single source at home/near
Crystal system	Monoclinic
Space group	*P*2/*c*
Unit cell dimensions	a = 9.15188(12) Å α = 90°
	b = 5.36460(6) Å β = 96.779(2)°
	c = 21.3814(4) Å γ = 90°
Volume	1042.41(3) Å^3
Z	4
Density (calculated)	1.250 Mg/m^3
Absorption coefficient	0.715 mm^{-1}
F(000)	424
Crystal habitus	plate (colourless)
Crystal size	0.235 x 0.083 x 0.022 mm^3
Theta range for data collection	4.164 to 77.540°
Index ranges	-11<=h<=11, -5<=k<=6, -26<=l<=26
Reflections collected	20575
Independent reflections	2195 [R(int) = 0.0248]
Completeness to theta = 67.684°	100.0 %
Absorption correction	Gaussian
Max. and min. transmission	1.000 and 0.789
Refinement method	Full-matrix least-squares on F^2
Data / restraints / parameters	2195 / 0 / 127
Goodness-of-fit on F^2	1.096
Final R indices [I>2sigma(I)]	R1 = 0.0364, wR2 = 0.0995
R indices (all data)	R1 = 0.0373, wR2 = 0.1002
Largest diff. peak and hole	0.325 and -0.184 e.Å^{-3}
Crystallisation details	A saturated solution of **3** in THF was layered with *n*hexane at –40°C under inert conditions.
Solution	SHELXT-2014/5 (G. M. Sheldrick, Acta Cryst., 2015, A71, 3-8)
Refinement	SHELXL-2018/3 (G. M. Sheldrick, Acta Cryst., 2008, A64, 112-122)
Interface	OLEX2 v1.2 (O. V. Dolomanov et al., J. Appl. Cryst., 2009, 42, 339-341)

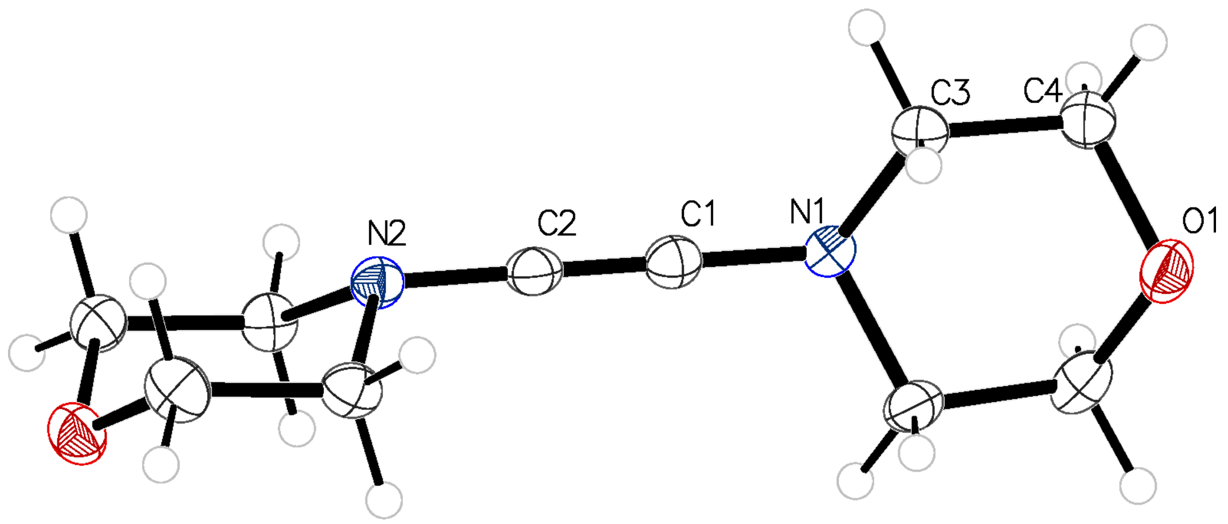

Figure S1: Molecular structure of **3** with thermal displacement parameters drawn at 50% probability.

S1.2 1,3,4,4-Tetramorpholinobut-1-yn-3-ene (**4**)

Compound	**4**
Identification code	2159700
Empirical formula	$C_{40}H_{64}N_8O_8$
Formula weight	784.99
Temperature	100(2) K
Wavelength	1.54184 Å
Instrument (scan mode)	XtaLAB Synergy, Single source at home/near
Crystal system	Triclinic
Space group	*P*-1
Unit cell dimensions	a = 5.9827(5) Å α = 71.137(5)°
	b = 17.0100(12) Å β = 83.344(6)°
	c = 21.2470(10) Å γ = 85.419(6)°
Volume	2030.2(3) Å^3
Z	2
Density (calculated)	1.284 Mg/m^3
Absorption coefficient	0.734 mm^{-1}
F(000)	848
Crystal habitus	needle (colorless)
Crystal size	0.190 x 0.037 x 0.031 mm^3
Theta range for data collection	2.208 to 77.874°
Index ranges	-7<=h<=7, -21<=k<=21, -26<=l<=26
Reflections collected	82889
Independent reflections	8470 [R(int) = 0.1463]
Completeness to theta = 67.684°	99.7 %
Absorption correction	Gaussian
Max. and min. transmission	1.000 and 0.698
Refinement method	Full-matrix least-squares on F^2
Data / restraints / parameters	8470 / 0 / 505
Goodness-of-fit on F^2	1.043
Final R indices [I>2sigma(I)]	R1 = 0.0753, wR2 = 0.1850
R indices (all data)	R1 = 0.1094, wR2 = 0.2106
Largest diff. peak and hole	0.408 and -0.456 e. Å^{-3}
Crystallisation details	A saturated solution of **4** in *n*hexane was allowed to slowly evaporate under inert conditions.
Solution	SHELXT-2014/5 (G. M. Sheldrick, Acta Cryst., 2015, A71, 3-8)
Refinement	SHELXL-2018/3 (G. M. Sheldrick, Acta Cryst., 2008, A64, 112-122)
Interface	OLEX2 v1.2 (O. V. Dolomanov et al., J. Appl. Cryst., 2009, 42, 339-341)

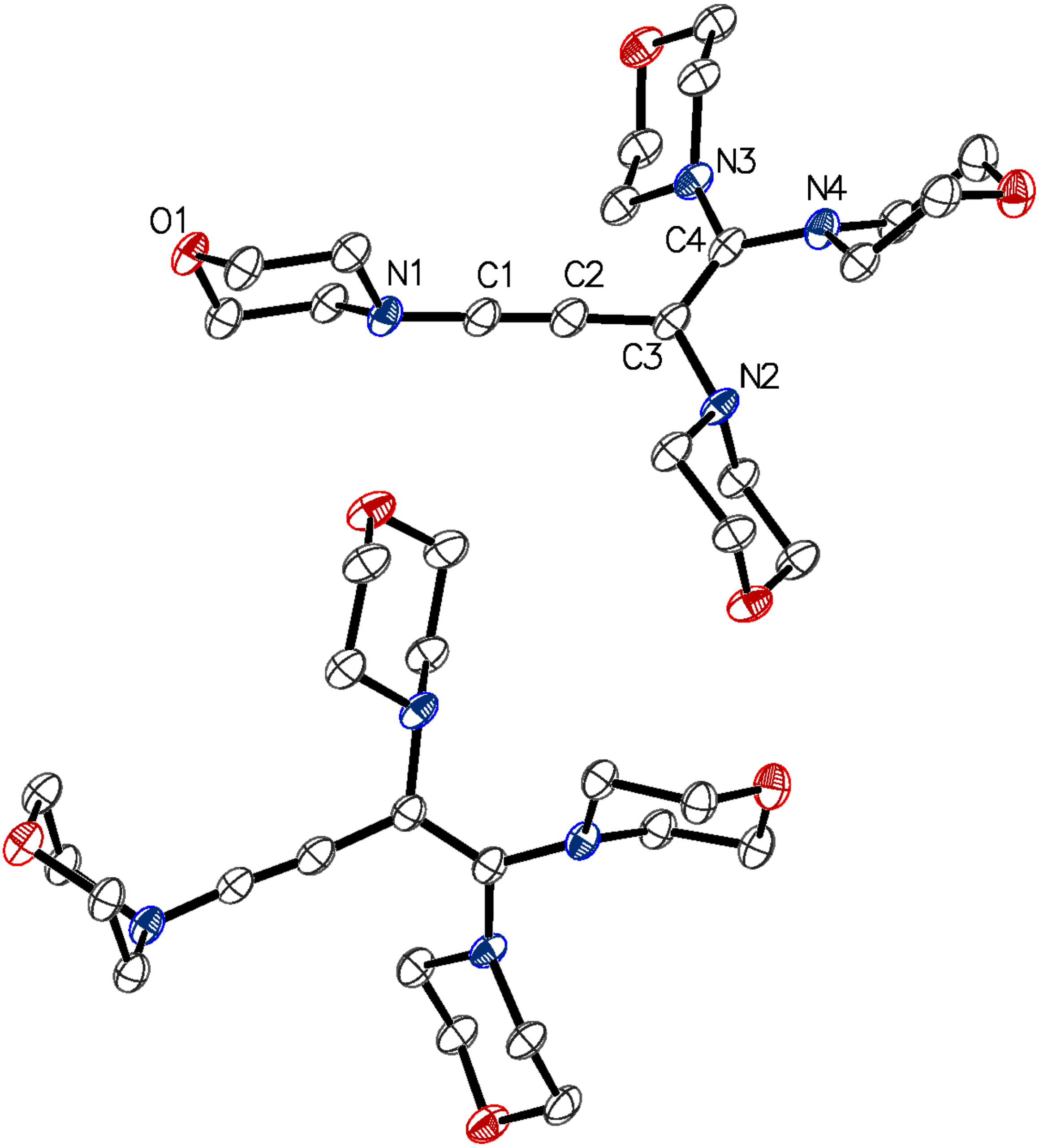

Figure S2: Molecular structure (asymmetric unit) of **4** with thermal displacement parameters drawn at 50% probability, hydrogen atoms are omitted for clarity.

S1.3 (CBA)AuCl (**5**)

Compound	**5**
Identification code	2159701
Empirical formula	$C_{20}H_{32}AuClN_4O_4$
Formula weight	624.91
Temperature	100(2) K
Wavelength	0.71073 Å
Instrument (scan mode)	XtaLAB Synergy, Single source at offset/far, HyPix ([illegible] scan)
Crystal system	Monoclinic
Space group	*I*2/*a*
Unit cell dimensions	a = 11.1565(3) Å [illegible]= 90°
	b = 11.8475(2) Å [illegible]= 107.046(3)°
	c = 18.2462(5) Å [illegible] = 90°
Volume	2305.77(10) Å^3
Z	4
Density (calculated)	1.800 Mg/m^3
Absorption coefficient	6.529 mm^{-1}
F(000)	1232
Crystal habitus	block (colourless)
Crystal size	0.180 x 0.080 x 0.060 mm^3
Theta range for data collection	2.078 to 41.402°
Index ranges	-20<=h<=20, -21<=k<=21, -33<=l<=33
Reflections collected	76737
Independent reflections	7628 [R(int) = 0.0351]
Completeness to theta = 25.242°	100.0 %
Absorption correction	Gaussian
Max. and min. transmission	1.000 and 0.370
Refinement method	Full-matrix least-squares on F^2
Data / restraints / parameters	7628 / 0 / 138
Goodness-of-fit on F^2	1.184
Final R indices [I>2sigma(I)]	R1 = 0.0273, wR2 = 0.0659
R indices (all data)	R1 = 0.0310, wR2 = 0.0669
Largest diff. peak and hole	4.054 and -1.480 e.Å^{-3}
Crystallisation details	A saturated solution of **5** in CH_2Cl_2 was layered with *n*hexane at room temperature under inert conditions.
Solution	SHELXT 2018/2 (G. M. Sheldrick, Acta Cryst., 2015, A71, 3-8)
Refinement	SHELXL-2018/3 (G. M. Sheldrick, Acta Cryst., 2015, C71, 3-8)
Interface	OLEX2 v1.3 (O. V. Dolomanov et al., J. Appl. Cryst., 2009, 42, 339-341)

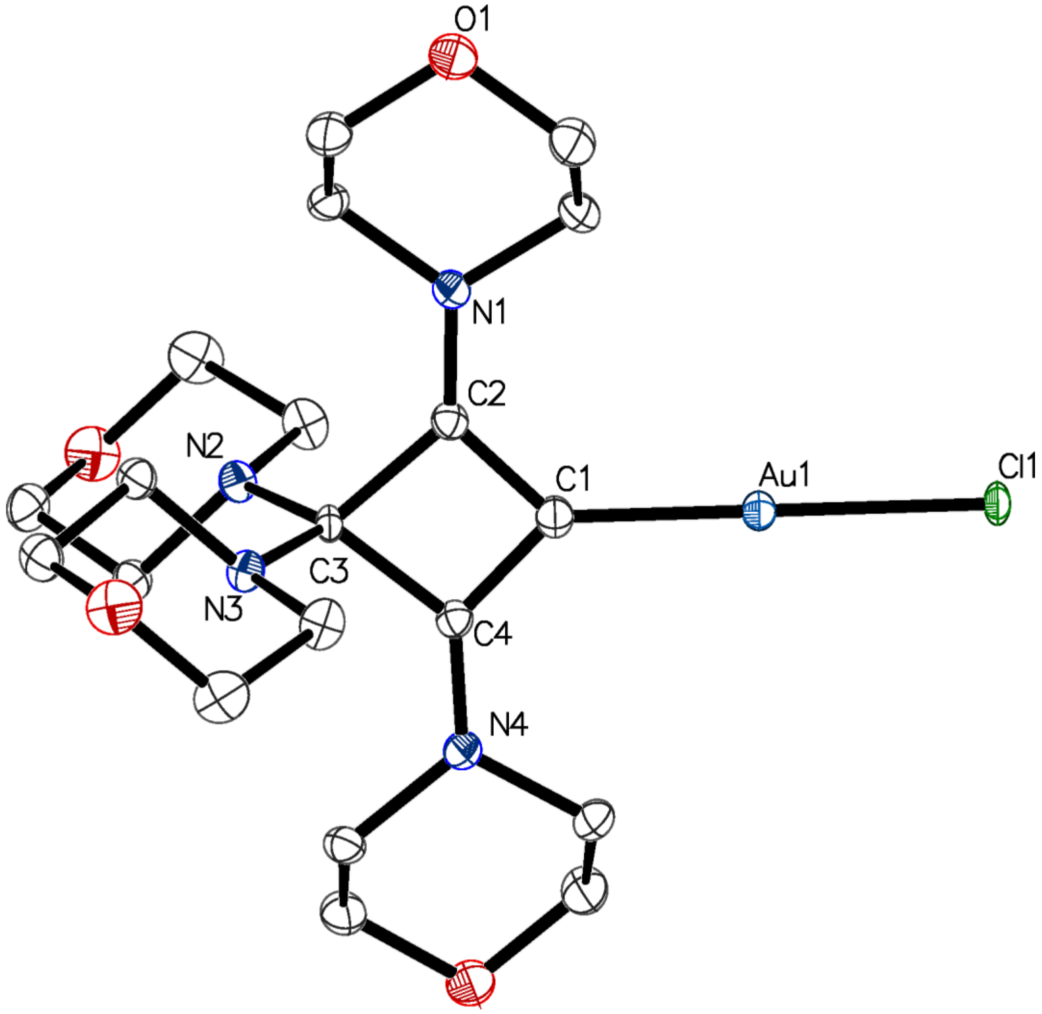

Figure S3: Molecular structure of **5** with thermal displacement parameters drawn at 50% probability, hydrogen atoms are omitted for clarity.

S1.4 (CBA)Rh(COD)Cl (**6**).

Compound	**6**
Identification code	2159702
Empirical formula	$C_{28}H_{44}ClN_4O_4Rh$
Formula weight	639.03
Temperature	100(2) K
Instrument (scan mode)	XtaLAB Synergy, Single source at offset/far
Crystal system	Orthorhombic
Space group	*Pbca*
Unit cell dimensions	a = 17.5960(2) Å α = 90°
	b = 17.0899(2) Å β = 90°
	c = 19.0313(2) Å γ = 90°
Volume	5722.98(11) $Å^3$
Z	8
Density (calculated)	1.483 Mg/m^3
Absorption coefficient	0.731 mm^{-1}
F(000)	2672
Crystal habitus	irregular (yellow)
Crystal size	0.218 x 0.152 x 0.133 mm^3
Theta range for data collection	1.976 to 28.282°
Index ranges	-23<=h<=23, -22<=k<=22, -25<=l<=25
Reflections collected	669602
Independent reflections	7103 [R(int) = 0.1129]
Completeness to theta = 25.242°	100.0 %
Absorption correction	Gaussian
Max. and min. transmission	1.000 and 0.325
Refinement method	Full-matrix least-squares on F^2
Data / restraints / parameters	7103 / 0 / 343
Goodness-of-fit on F^2	1.060
Final R indices [I>2sigma(I)]	R1 = 0.0431, wR2 = 0.1147
R indices (all data)	R1 = 0.0478, wR2 = 0.1183
Largest diff. peak and hole	1.533 and -0.793 e. $Å^{-3}$
Crystallisation details	A saturated solution of **6** in CH_2Cl_2 was layered with *n*pentane at room temperature under inert conditions.
Solution	SHELXT-2014/5 (G. M. Sheldrick, Acta Cryst., 2015, A71, 3-8)
Refinement	SHELXL-2018/3 (G. M. Sheldrick, Acta Cryst., 2008, A64, 112-122)
Interface	OLEX2 v1.2 (O. V. Dolomanov et al., J. Appl. Cryst., 2009, 42, 339-341)

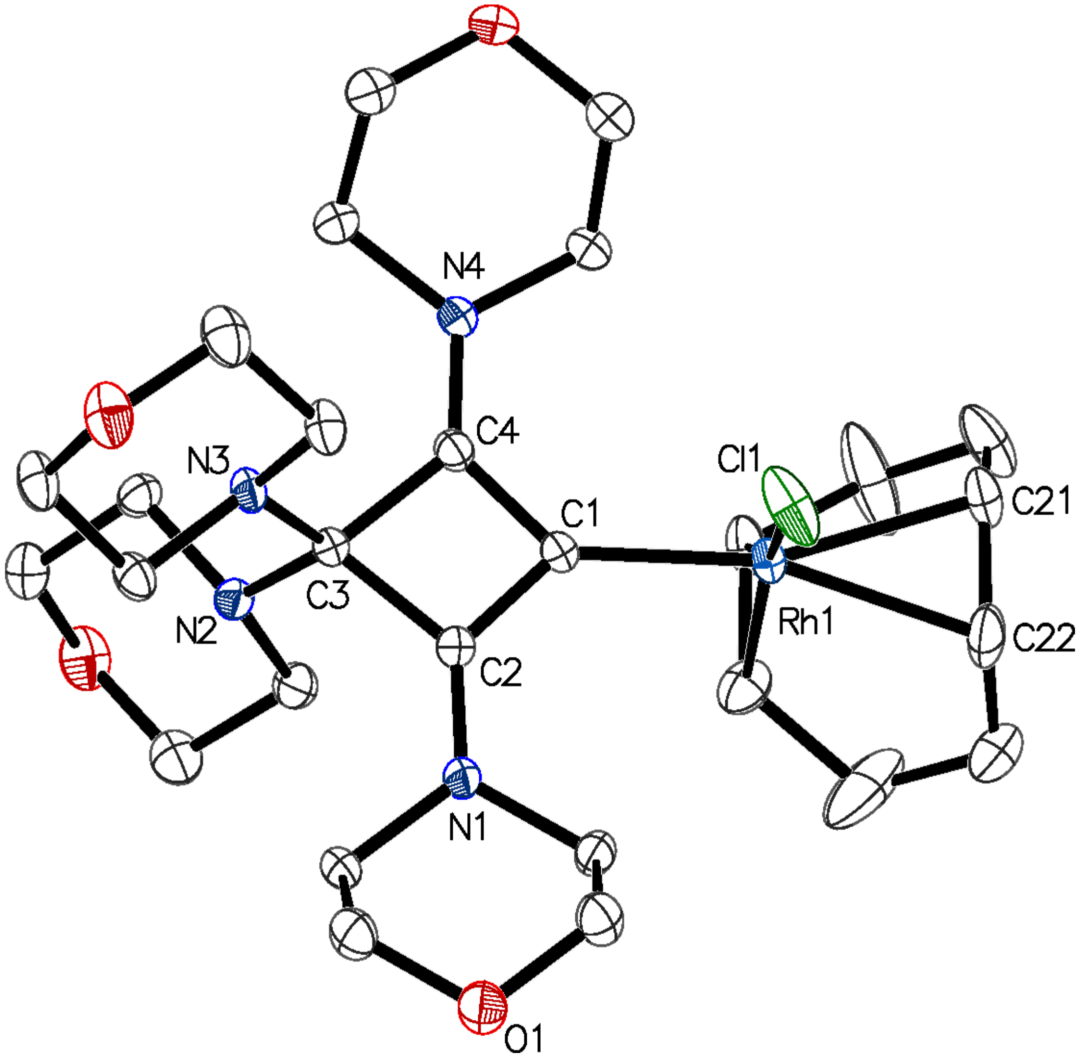

Figure S4: Molecular structure of **6** with thermal displacement parameters drawn at 50% probability, hydrogen atoms are omitted for clarity.

S1.5 (CBA)Rh(CO)$_2$Cl (**7**)

Compound	**7**
Identification code	2159703
Empirical formula	$C_{22}H_{32}ClN_4O_6Rh$
Formula weight	586.87
Temperature	100(2) K
Wavelength	0.71073 Å
Instrument (scan mode)	XtaLAB Synergy, Single source at offset/far
Crystal system	Orthorhombic
Space group	*Pbca*
Unit cell dimensions	a = 16.4159(2) Å α = 90°
	b = 16.9489(2) Å β = 90°
	c = 18.6345(3) Å γ = 90°
Volume	5184.70(12) Å^3
Z	8
Density (calculated)	1.504 Mg/m^3
Absorption coefficient	0.805 mm^{-1}
F(000)	2416
Crystal habitus	prism (yellow)
Crystal size	0.258 x 0.205 x 0.175 mm^3
Theta range for data collection	2.514 to 28.282°
Index ranges	-21<=h<=21, -22<=k<=21, -24<=l<=24
Reflections collected	265785
Independent reflections	6433 [R(int) = 0.0882]
Completeness to theta = 25.242°	99.9 %
Absorption correction	Gaussian
Max. and min. transmission	1.000 and 0.364
Refinement method	Full-matrix least-squares on F^2
Data / restraints / parameters	6433 / 30 / 344
Goodness-of-fit on F^2	1.291
Final R indices [I>2sigma(I)]	R1 = 0.0627, wR2 = 0.1276
R indices (all data)	R1 = 0.0676, wR2 = 0.1292
Largest diff. peak and hole	1.032 and -1.351 e. Å^{-3}
Crystallisation details	A saturated solution of **7** in THF was layered with *n*-hexane at room temperature under inert conditions.
Solution	SHELXT-2014/5 (G. M. Sheldrick, Acta Cryst., 2015, A71, 3-8)
Refinement	SHELXL-2018/3 (G. M. Sheldrick, Acta Cryst., 2008, A64, 112-122)
Interface	OLEX2 v1.2 (O. V. Dolomanov et al., J. Appl. Cryst., 2009, 42, 339-341)
Measurement and Refinement Details	One morpholine moiety is disordered and was refined over two positions.

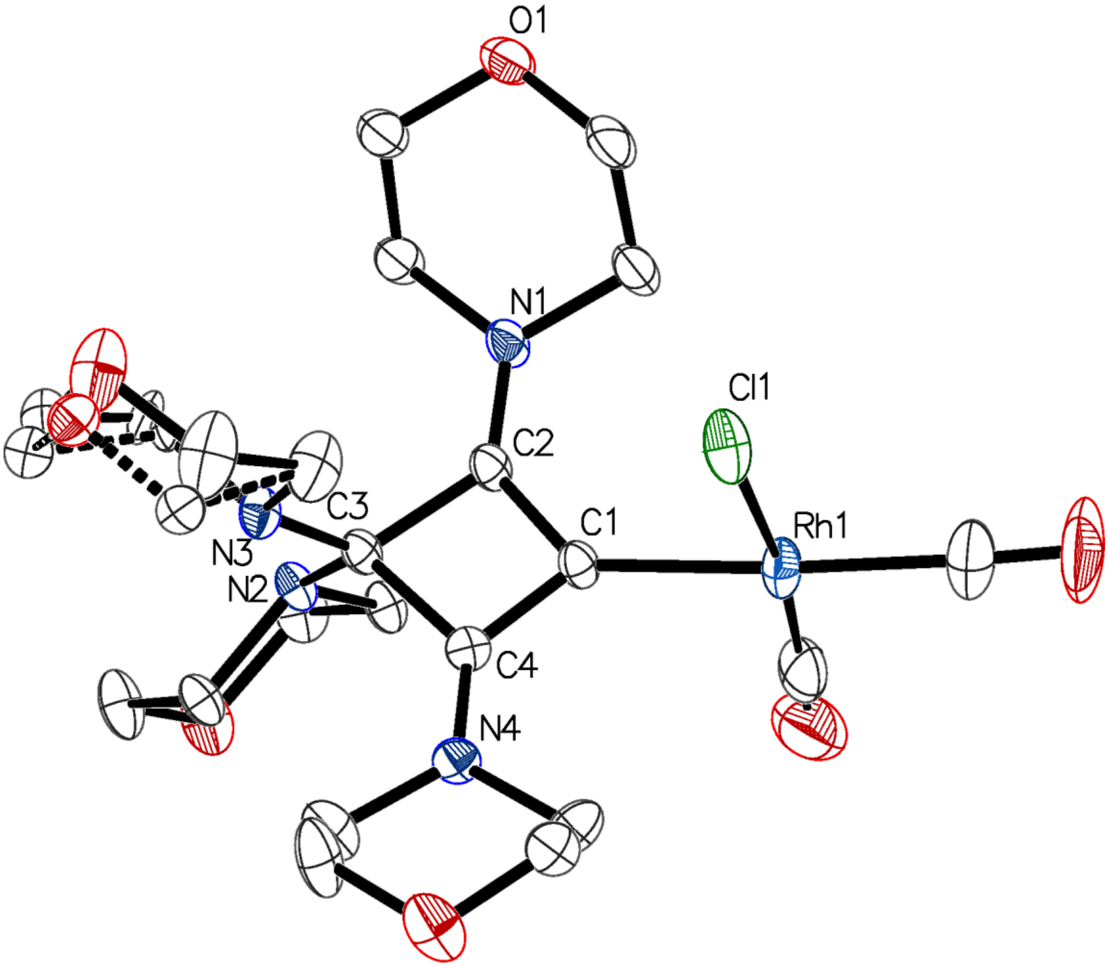

Figure S5: Molecular structure of **7** with thermal displacement parameters drawn at 50% probability, hydrogen atoms are omitted for clarity.

S1.6 1,2,3,4-Tetramorpholinocyclobutenylium bromide (**8**).

Compound	**8**
Identification code	2159704
Empirical formula	$C_{20}H_{33}BrN_4O_4$
Formula weight	473.41
Temperature	100(2) K
Wavelength	1.54184 Å
Instrument (scan mode)	XtaLAB Synergy, Single source at home/near
Crystal system	Monoclinic
Space group	*P*2₁/*c*
Unit cell dimensions	a = 13.8197(2) Å α = 90°
	b = 10.1590(2) Å β = 102.016(2)°
	c = 15.5767(2) Å γ = 90°
Volume	2138.96(6) Å^3
Z	4
Density (calculated)	1.470 Mg/m^3
Absorption coefficient	2.904 mm^{-1}
F(000)	992
Crystal habitus	block (colourless)
Crystal size	0.261 x 0.218 x 0.084 mm^3
Theta range for data collection	3.270 to 77.714°
Index ranges	-17<=h<=17, -12<=k<=12, -19<=l<=19
Reflections collected	83397
Independent reflections	4513 [R(int) = 0.0296]
Completeness to theta = 67.684°	100.0 %
Absorption correction	Gaussian
Max. and min. transmission	1.000 and 0.327
Refinement method	Full-matrix least-squares on F^2
Data / restraints / parameters	4513 / 0 / 262
Goodness-of-fit on F^2	1.064
Final R indices [I>2sigma(I)]	R1 = 0.0249, wR2 = 0.0634
R indices (all data)	R1 = 0.0251, wR2 = 0.0635
Largest diff. peak and hole	0.345 and -0.469 e. Å^{-3}
Crystallisation details	A saturated solution of **8** in CH_2Cl_2 was layered with *n*hexane at ambient temperatures under inert conditions.
Solution	SHELXT-2014/5 (G. M. Sheldrick, Acta Cryst., 2015, A71, 3-8)
Refinement	SHELXL-2018/3 (G. M. Sheldrick, Acta Cryst., 2008, A64, 112-122)
Interface	OLEX2 v1.2 (O. V. Dolomanov et al., J. Appl. Cryst., 2009, 42, 339-341)

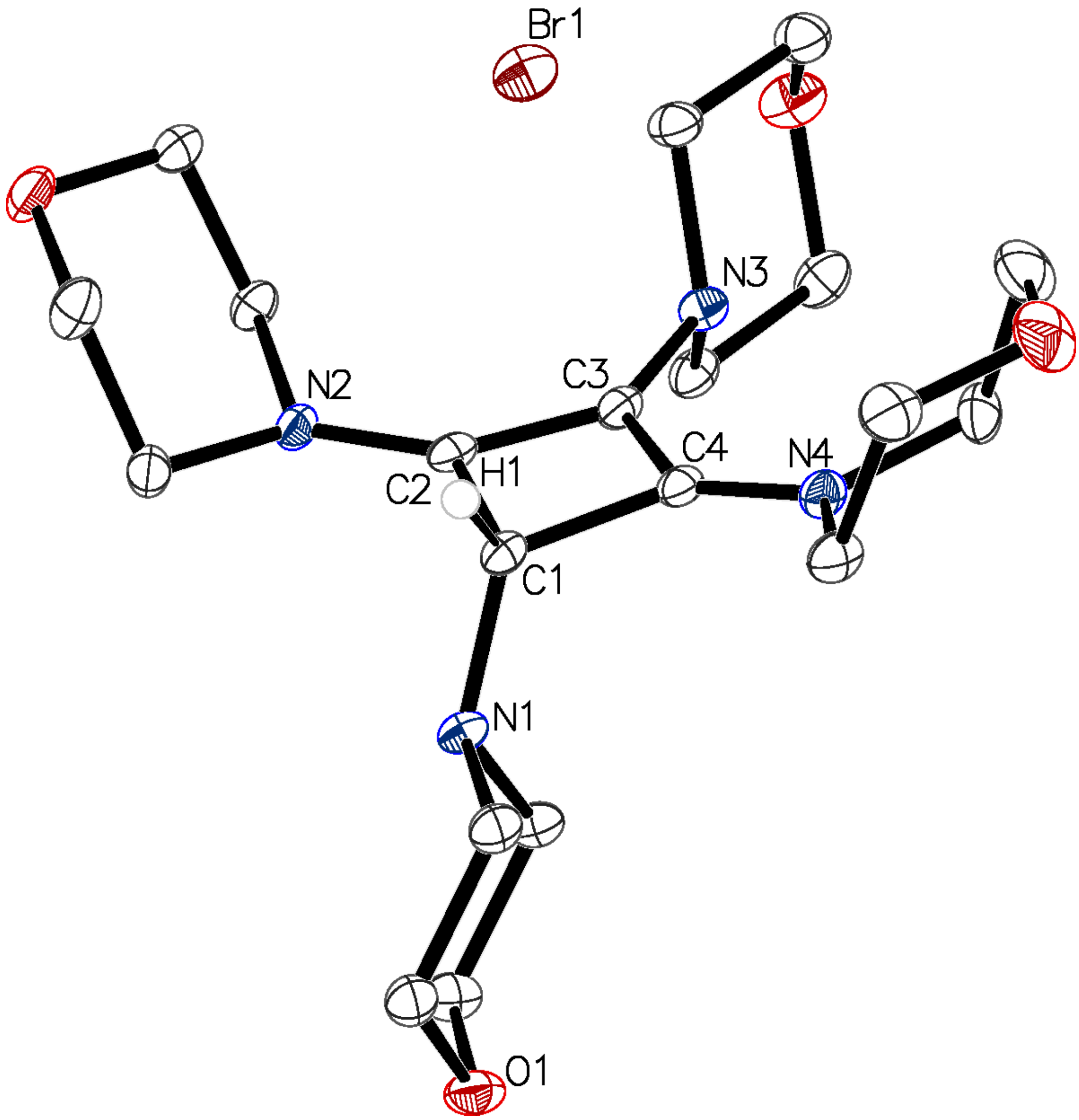

Figure S6: Molecular structure of **8** with thermal displacement parameters drawn at 50% probability, hydrogen atoms, except H1, are omitted for clarity.

S1.7 Tetramorpholinocyclobutenediylium bis(tribromide) (**9**).

Compound	**9**
Identification code	2159705
Empirical formula	$C_{20}H_{32}Br_6N_4O_4$
Formula weight	871.95
Temperature	100(2) K
Wavelength	1.54184 Å
Instrument (scan mode)	XtaLAB Synergy, Single source at home/near
Crystal system	Monoclinic
Space group	*Cc*
Unit cell dimensions	a = 13.48707(2) Å α = 90°
	b = 11.51191(2) Å β = 100.4481(2)°
	c = 18.68011(5) Å γ = 90°
Volume	2852.223(10) $Å^3$
Z	4
Density (calculated)	2.031 Mg/m^3
Absorption coefficient	10.452 mm^{-1}
F(000)	1688
Crystal habitus	irregular (orange)
Crystal size	0.179 x 0.149 x 0.105 mm^3
Theta range for data collection	4.815 to 77.614°
Index ranges	-17<=h<=17, -14<=k<=14, -19<=l<=22
Reflections collected	112383
Independent reflections	5700 [R(int) = 0.0286]
Completeness to theta = 67.684°	100.0 %
Absorption correction	Gaussian
Max. and min. transmission	0.717 and 0.223
Refinement method	Full-matrix least-squares on F^2
Data / restraints / parameters	5700 / 2 / 308
Goodness-of-fit on F^2	1.081
Final R indices [I>2sigma(I)]	R1 = 0.0271, wR2 = 0.0725
R indices (all data)	R1 = 0.0271, wR2 = 0.0725
Largest diff. peak and hole	0.910 and -0.725 e. $Å^{-3}$
Crystallisation details	EtOAc was layered with a saturated solution of **9** in MeCN at -40 °C and stored under inert conditions.
Solution	SHELXT-2014/5 (G. M. Sheldrick, Acta Cryst., 2015, A71, 3-8)
Refinement	SHELXL-2018/3 (G. M. Sheldrick, Acta Cryst., 2008, A64, 112-122)
Interface	OLEX2 v1.2 (O. V. Dolomanov et al., J. Appl. Cryst., 2009, 42, 339-341)

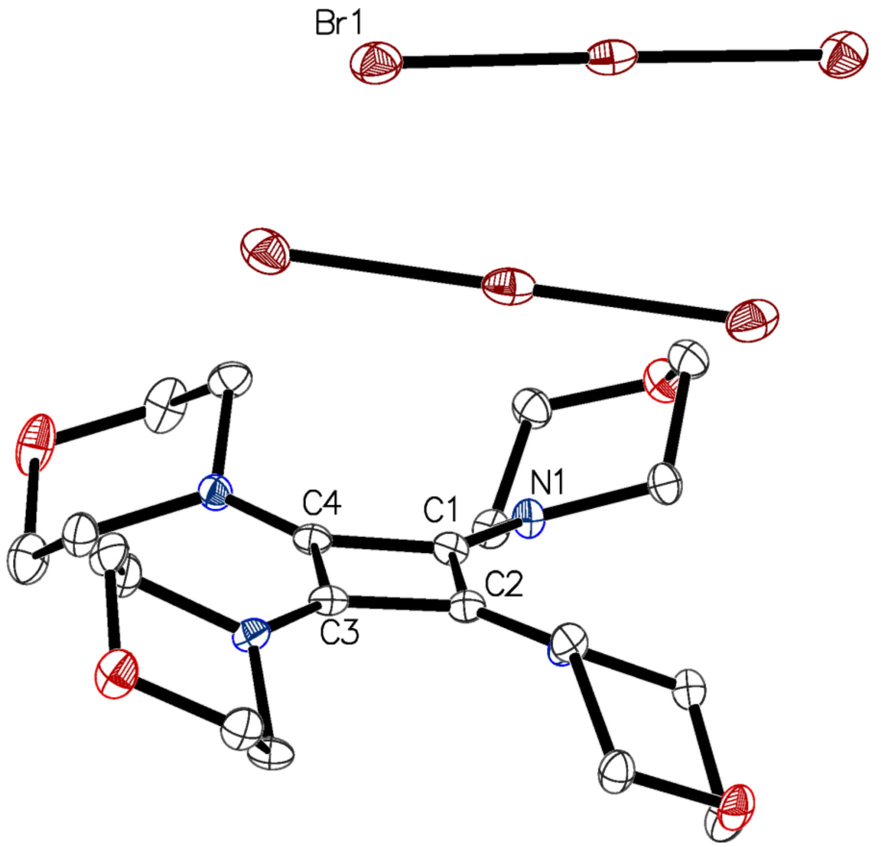

Figure S7: Molecular structure of **9** with thermal displacement parameters drawn at 50% probability, hydrogen atoms are omitted for clarity.

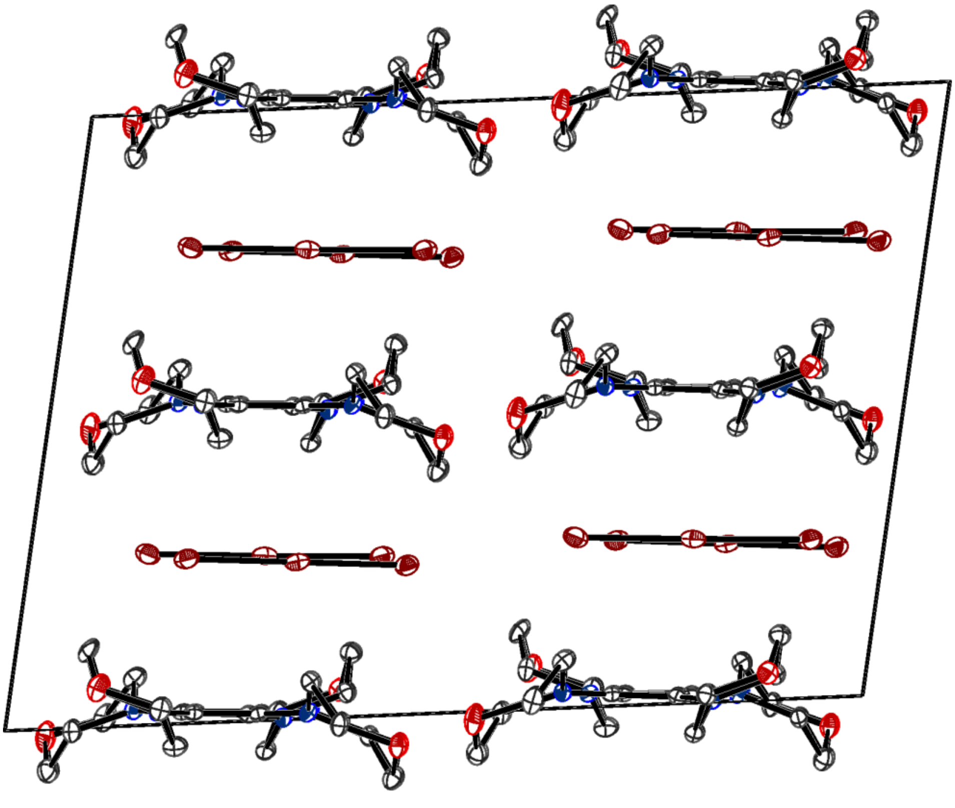

Figure S8: Packing diagram of compound **9**. Looking along the c axis, a stacked arrangement of alternating cyclobutadiene dications and Br_3 anions is observed.

S1.8 1,1-Dibromo-2,2-dimorpholinoethyl bromide (**S1**·$CHCl_3$)

Compound	**S1**·$CHCl_3$
Identification code	2159706
Empirical formula	$C_{11}H_{18}Br_3Cl_3N_2O_2$
Formula weight	556.35
Temperature	100(2) K
Wavelength	1.54184 Å
Instrument (scan mode)	XtaLAB Synergy, Single source at home/near
Crystal system	Orthorhombic
Space group	*Pbca*
Unit cell dimensions	a = 12.5966(4) Å [illegible]= 90°
	b = 14.3284(3) Å [illegible]= 90°
	c = 21.0649(5) Å [illegible] = 90°
Volume	3802.01(17) $Å^3$
Z	8
Density (calculated)	1.944 Mg/m^3
Absorption coefficient	11.799 mm^{-1}
F(000)	2160
Crystal habitus	irregular (colourless)
Crystal size	0.280 x 0.189 x 0.073 mm^3
Theta range for data collection	4.198 to 77.332°
Index ranges	-15<=h<=13, -18<=k<=18, -26<=l<=26
Reflections collected	148503
Independent reflections	4012 [R(int) = 0.0463]
Completeness to theta = 67.684°	100.0 %
Absorption correction	Gaussian
Max. and min. transmission	0.894 and 0.097
Refinement method	Full-matrix least-squares on F^2
Data / restraints / parameters	4012 / 0 / 191
Goodness-of-fit on F^2	1.244
Final R indices [I>2sigma(I)]	R1 = 0.0222, wR2 = 0.0545
R indices (all data)	R1 = 0.0222, wR2 = 0.0545
Largest diff. peak and hole	0.429 and -0.448 e.$Å^{-3}$
Crystallisation details	lk07ph was dissolved in $CDCl_3$ and was stored in a NMR tube for several days.
Solution	SHELXT-2014/5 (G. M. Sheldrick, Acta Cryst., 2015, A71, 3-8)
Refinement	SHELXL-2018/3 (G. M. Sheldrick, Acta Cryst., 2008, A64, 112-122)
Interface	OLEX2 v1.2 (O. V. Dolomanov et al., J. Appl. Cryst., 2009, 42, 339-341)

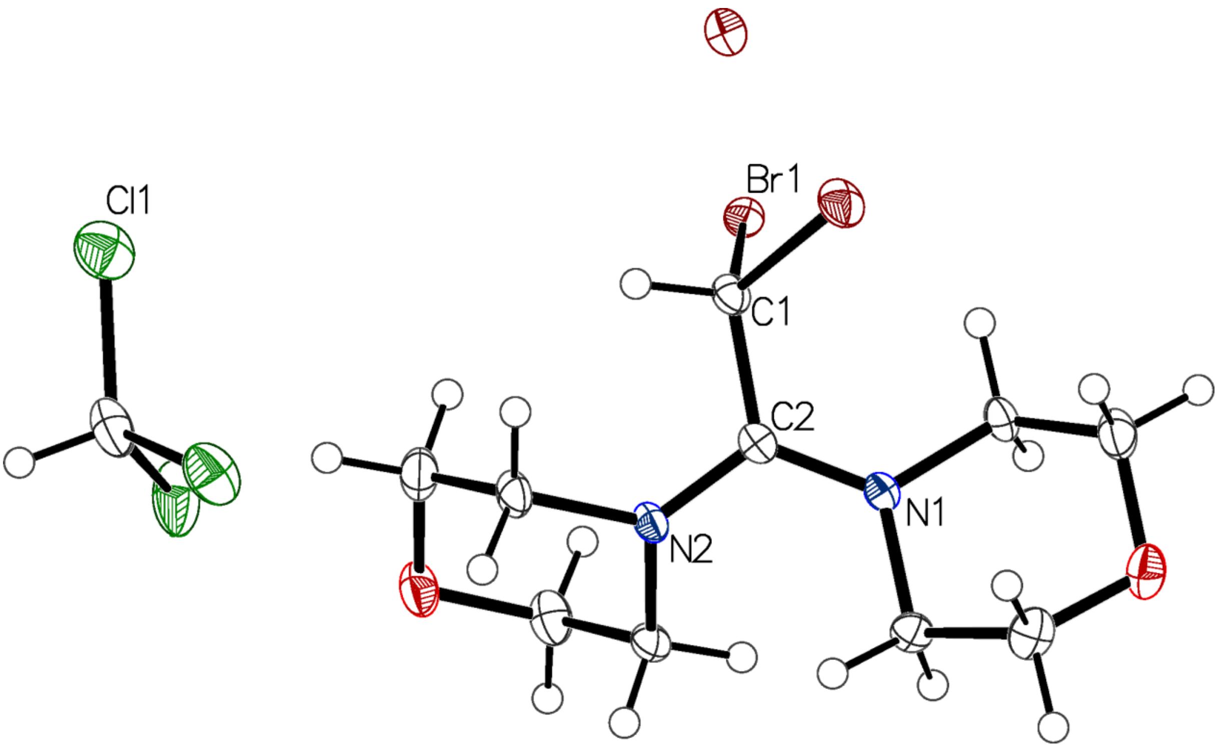

Figure S9: Molecular structure of **S1**·$CHCl_3$ with thermal displacement parameters drawn at 50% probability, hydrogen atoms are omitted for clarity. Selected bond lengths and angles: C1-C2 1.506(3), C2-N1 1.329(3), C2-N2 1.335(3), C1-Br1 1.963(2), N1-C2-N2 121.7(2), C1-C2-N1-N2 177.4(4).

When 1,1-Dibromo-2,2-dimorpholinoethene (**2**) was stored for several days in a chloroform NMR solution in air, the formation of colourless single crystals on the wall of the NMR tube was observed. X-ray diffraction analysis revealed the molecular structure of **S1**·$CHCl_3$. The formation of this compound can be explained by the slow decomposition of **2** due to residual HCl in the solvent and contact with atmospheric moisture which leads to protonation in C1 position leaving a carbocationic C2 atom, which is planarized, with bromide probably from a second equivalent of **2** as a counter ion.

S1.11 [$(CBA)_2$Au]Cl (**S2**)

Compound	**S2**·1.5DCM
Identification code	2159709
Empirical formula	$C_{21.50}H_{36}Au_{0.50}Cl_{3.50}N_4O_4$
Formula weight	637.10
Temperature	373(2) K
Wavelength	0.71073 Å
Instrument (scan mode)	XtaLAB Synergy, Single source at offset/far
Crystal system	Monoclinic
Space group	*C*2/*c*
Unit cell dimensions	a = 28.4607(5) Å α = 90°
	b = 17.0100(12) Å β = 108.4232(17)°
	c = 21.7961(3) Å γ = 90°
Volume	5315.83(17) $Å^3$
Z	8
Density (calculated)	1.592 Mg/m^3
Absorption coefficient	3.176 mm^{-1}
F(000)	2592
Crystal habitus	plate (colourless)
Crystal size	0.099 x 0.085 x 0.020 mm^3
Theta range for data collection	1.508 to 28.281°
Index ranges	-37<=h<=37, -12<=k<=12, -29<=l<=28
Reflections collected	190218
Independent reflections	6590 [R(int) = 0.1098]
Completeness to theta = 25.242°	100.0 %
Absorption correction	Gaussian
Max. and min. transmission	1.000 and 0.721
Refinement method	Full-matrix least-squares on F^2
Data / restraints / parameters	6590 / 129 / 374
Goodness-of-fit on F^2	1.084
Final R indices [I>2sigma(I)]	R1 = 0.0414, wR2 = 0.1030
R indices (all data)	R1 = 0.0561, wR2 = 0.1093
Largest diff. peak and hole	1.886 and -1.172 e. $Å^{-3}$
Crystallisation details	A saturated solution in CH_2Cl_2 was layered with *n*hexane at ambient temperatures under inert conditions.
Solution	SHELXT-2014/5 (G. M. Sheldrick, Acta Cryst., 2015, A71, 3-8)
Refinement	SHELXL-2018/3 (G. M. Sheldrick, Acta Cryst., 2008, A64, 112-122)
Interface	OLEX2 v1.2 (O. V. Dolomanov et al., J. Appl. Cryst., 2009, 42, 339-341)
Measurement and Refinement Details	One DCM molecule is disordered over several positions and was refined as such. This DCM molecule is only half occupied, hence the non-integer number of C and Cl atoms in the sum formula. The Au atom is located on a special position and is therefore only half occupied per asymmetric unit.

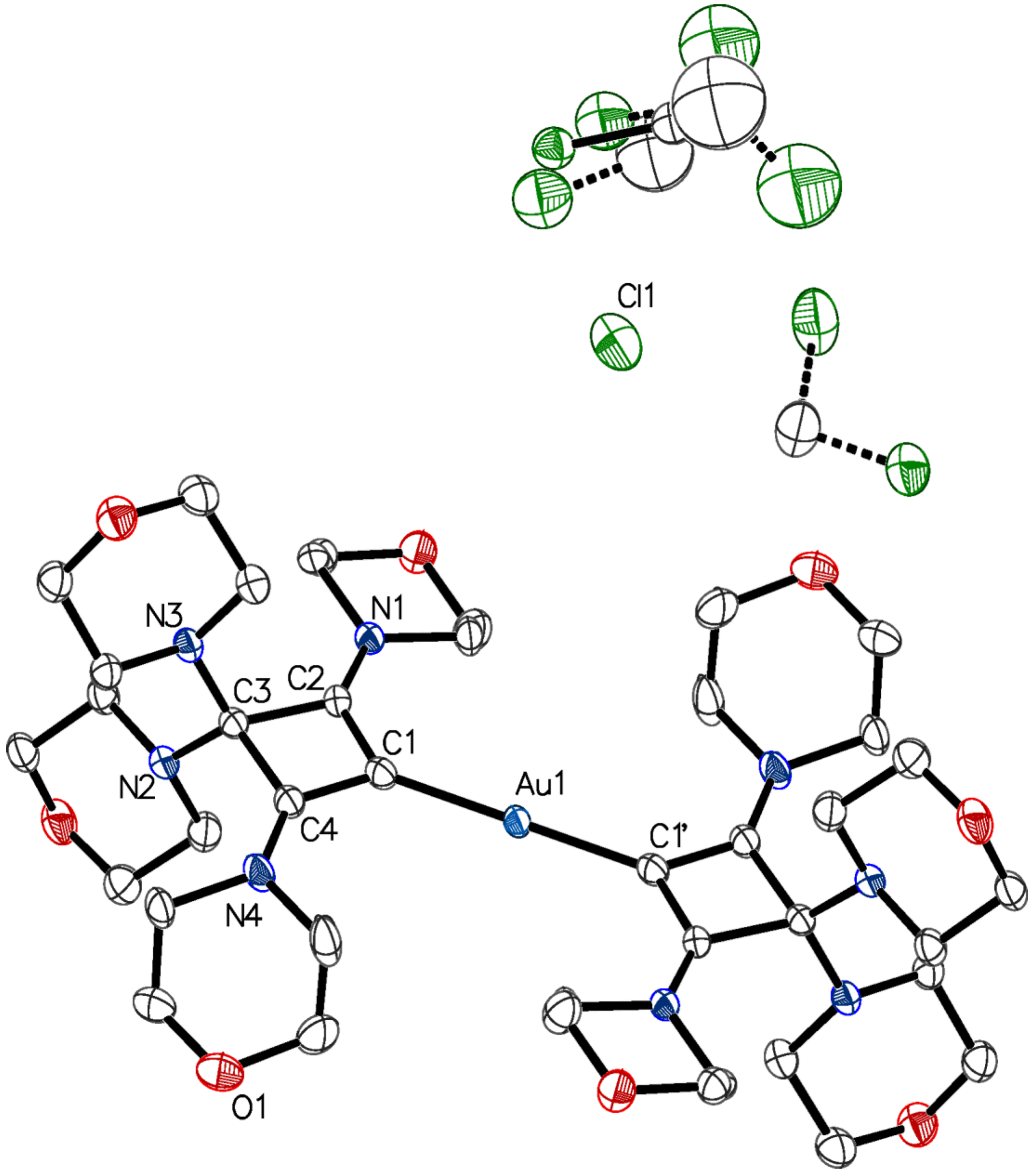

Figure S10: Molecular structure of **S4** with thermal displacement parameters drawn at 50% probability, hydrogen atoms are omitted for clarity; Selected bond lengths and angles: C1–Au1 2.033(4), C1–C2 1.406(6), C2–C3 1.546(5), C3–C4 1.543(5), C4–C1 1.412(6), C2–N1 1.320(5), C3–N2 1.462(5), C3–N3 1.454(5), C4–N4 1.327(5), C1–Au1–C1ʻ 180.0(2), C2–C1–C4 88.6(3), C1–C4–C3 96.1(3), C4–C3–C2 79.1(3), C3–C2–C1 96.2(3), C1–C2–C3–C4 1.8(3).

A second crystal structure as the result of the reaction of enyne **4** and (THT)AuCl was determined. When a DCM solution was layered with n-hexanes at ambient temperature, crystals suitable for X-ray diffraction analysis were obtained which revealed the molecular structure of **S2**·1.5DCM which exhibits a homoleptic CBA-Au(I) complex with an uncoordinated chloride counter ion. The synthesis of this compound could not be achieved selectively and its formation is assumed to be an artefact due to residual free enyne **4** in the crystallization solution.

S1.9 1-Bromo-3-oxo-2,4-dimorpholinocyclobutenylium tribromide (**S3**)

Compound	**S3**
Identification code	2159707
Empirical formula	$C_{12}H_{16}Br_4N_2O_3$
Formula weight	555.91
Temperature	100(2) K
Wavelength	0.71073 Å
Instrument (scan mode)	XtaLAB Synergy, Single source at offset/far
Crystal system	Monoclinic
Space group	*C*2/*c*
Unit cell dimensions	a = 21.9648(6) Å α = 90°
	b = 8.0280(2) Å β = 111.416(4)°
	c = 20.8590(6) Å γ = 90°
Volume	3424.18(18) Å^3
Z	8
Density (calculated)	2.157 Mg/m^3
Absorption coefficient	9.412 mm^{-1}
F(000)	2128
Crystal habitus	prism (orange)
Crystal size	0.811 x 0.379 x 0.194 mm^3
Theta range for data collection	1.992 to 35.845°
Index ranges	-35<=h<=35, -13<=k<=12, -33<=l<=34
Reflections collected	133184
Independent reflections	7654 [R(int) = 0.0878]
Completeness to theta = 25.242°	100.0 %
Absorption correction	Gaussian
Max. and min. transmission	0.489 and 0.007
Refinement method	Full-matrix least-squares on F^2
Data / restraints / parameters	7654 / 0 / 192
Goodness-of-fit on F^2	1.046
Final R indices [I>2sigma(I)]	R1 = 0.0259, wR2 = 0.0615
R indices (all data)	R1 = 0.0351, wR2 = 0.0637
Largest diff. peak and hole	1.287 and -0.637 e.Å^{-3}
Crystallisation details	Crystals were obtained from a CH_2Cl_2 solution at room temperature by storing overnight.
Solution	SHELXT-2014/5 (G. M. Sheldrick, Acta Cryst., 2015, A71, 3-8)
Refinement	SHELXL-2018/3 (G. M. Sheldrick, Acta Cryst., 2008, A64, 112-122)
Interface	OLEX2 v1.2 (O. V. Dolomanov et al., J. Appl. Cryst., 2009, 42, 339-341)

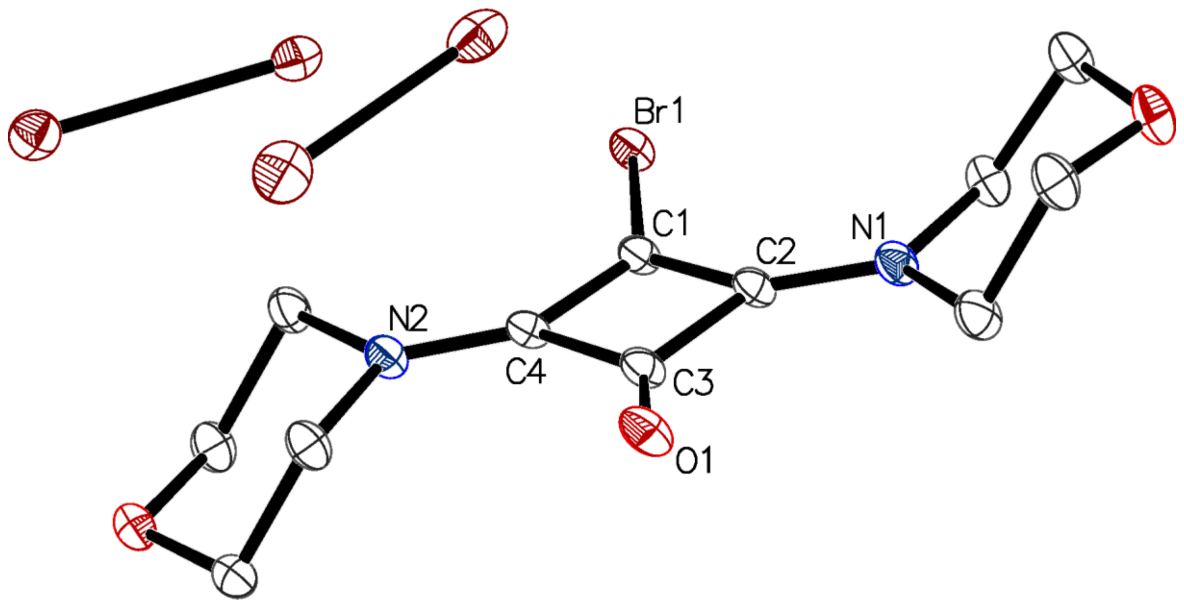

Figure 11: Molecular structure of **S2** with thermal displacement parameters drawn at 50% probability, hydrogen atoms are omitted for clarity; Selected bond lengths and angles: C1-C2 1.418(2), C2-C3 1.505(2), C3-C4 1.509(2), C4-C1 1.420(2), C2-N1 1.303(2), C1-Br1 1.846(1), C3-O1 1.211(2), C1-C2-C3 89.44(12), C2-C3-C4 87.17(11), C1-C2-C3-C4 0.42(11).

When the DCM solution which was separated during the synthesis of **9** was stored overnight under inert conditions, the formation of orange single crystals was observed. X-ray diffraction analysis revealed the molecular structure of **S3**. There are two mono anionic Br_3^- units which are each half occupied, which results one anionic counter charge per asymmetric unit. Hence, the cyclobutadiene fragment is mono cationic. It shows two morpholino, one oxo and one bromo substituent. It should be regarded as a cyclobutadienone with a π electron delocalisation over the N1-C2-C1-C4-N2 moiety. This side product probably formed through hydrolysis due to residual moisture which entered the reaction mixture by the bromine that is stored in air.

S1.10 1-Oxotrimorpholinocyclobutenylium tribromide (**S4**)

Compound	**S4**
Identification code	2159708
Empirical formula	$C_{16}H_{24}Br_3N_3O_4$
Formula weight	562.11
Temperature	110(2) K
Wavelength	1.54184 Å
Instrument (scan mode)	XtaLAB Synergy, Single source at home/near
Crystal system	Monoclinic
Space group	$P2_1/n$
Unit cell dimensions	a = 9.9406(2) Å α = 90°
	b = 7.3538(2) Å β = 99.741(2)°
	c = 28.5913(6) Å [illegible] = 90°
Volume	2059.92(8) $Å^3$
Z	4
Density (calculated)	1.813 Mg/m^3
Absorption coefficient	7.493 mm^{-1}
F(000)	1112
Crystal habitus	needle (colourless)
Crystal size	0.116 x 0.052 x 0.014 mm^3
Theta range for data collection	3.137 to 77.601°
Index ranges	-12<=h<=12, -9<=k<=8, -36<=l<=36
Reflections collected	153442
Independent reflections	4365 [R(int) = 0.0580]
Completeness to theta = 67.684°	100.0 %
Absorption correction	Gaussian
Max. and min. transmission	1.000 and 0.569
Refinement method	Full-matrix least-squares on F^2
Data / restraints / parameters	4365 / 0 / 235
Goodness-of-fit on F^2	1.077
Final R indices [I>2sigma(I)]	R1 = 0.0352, wR2 = 0.0927
R indices (all data)	R1 = 0.0369, wR2 = 0.0939
Largest diff. peak and hole	1.765 and -0.887 e.$Å^{-3}$
Crystallisation details	A solution of **S3** in THF was layered with *n*hexane at ambient temperatures under inert conditions.
Solution	SHELXT-2014/5 (G. M. Sheldrick, Acta Cryst., 2015, A71, 3-8)
Refinement	SHELXL-2018/3 (G. M. Sheldrick, Acta Cryst., 2008, A64, 112-122)
Interface	OLEX2 v1.2 (O. V. Dolomanov et al., J. Appl. Cryst., 2009, 42, 339-341)

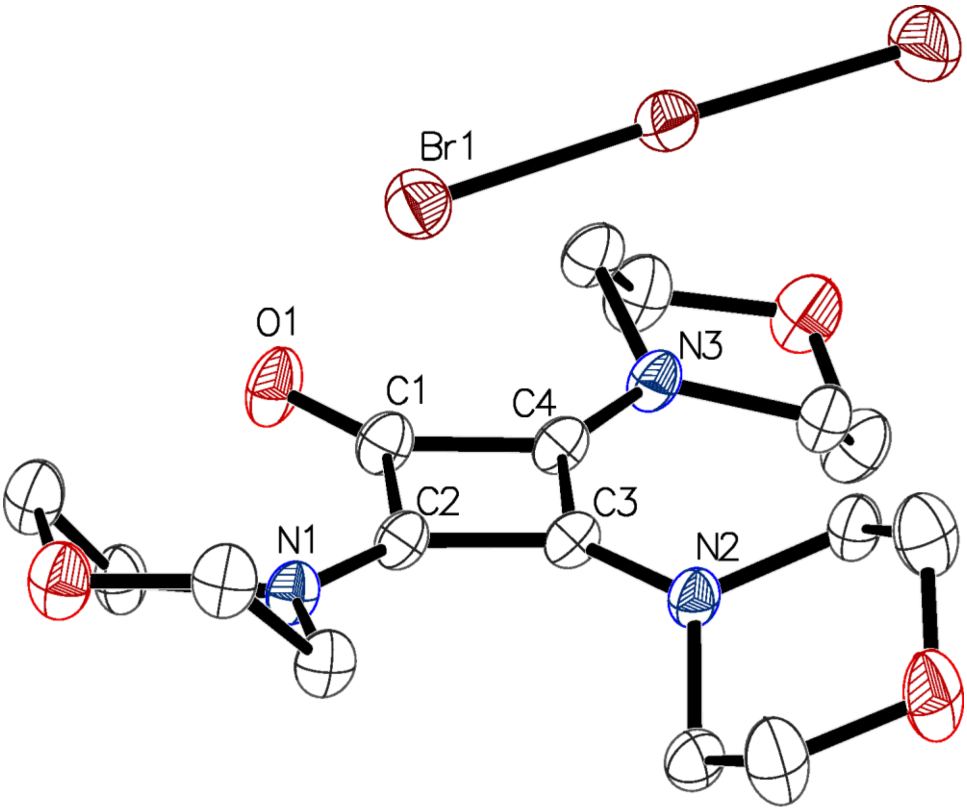

Figure S12: Molecular structure of **S4** with thermal displacement parameters drawn at 50% probability, hydrogen atoms are omitted for clarity; Selected bond lengths and angles: C1-C2 1.479(4), C2-C3 1.439(4), C3-C4 1.445(4), 1.478(4), C1-O1 1.216(4), C2-N1 1.311(4), C3-N2 1.356(4), C4-N3 1.316(4), C1-C2-C3-C4 0.0(2).

Out of a THF solution which was layered with *n*-hexanes under inert conditions at ambient temperature a second side product of the synthesis of **9** was crystallized. X-ray diffraction analysis revealed the molecular structure of **S4** which can also be regarded as a cyclobutadienone mono cation. It is comparable to a hydrolysis product which was reported by Viehe in the same paper as the cyclobutadiene dication.[3] Interestingly, in contrast to **S3**, the C-C bond lengths of the C_4 ring only differ slightly from each other. Therefor the contribution of the C1 atom into the π delocalisation is possible which would describe the C_4 ring as another cyclobutadiene dicationic derivative.

.

S2 NMR Spectra

S2.1 1,1-Dimorpholinoethene (**1**)

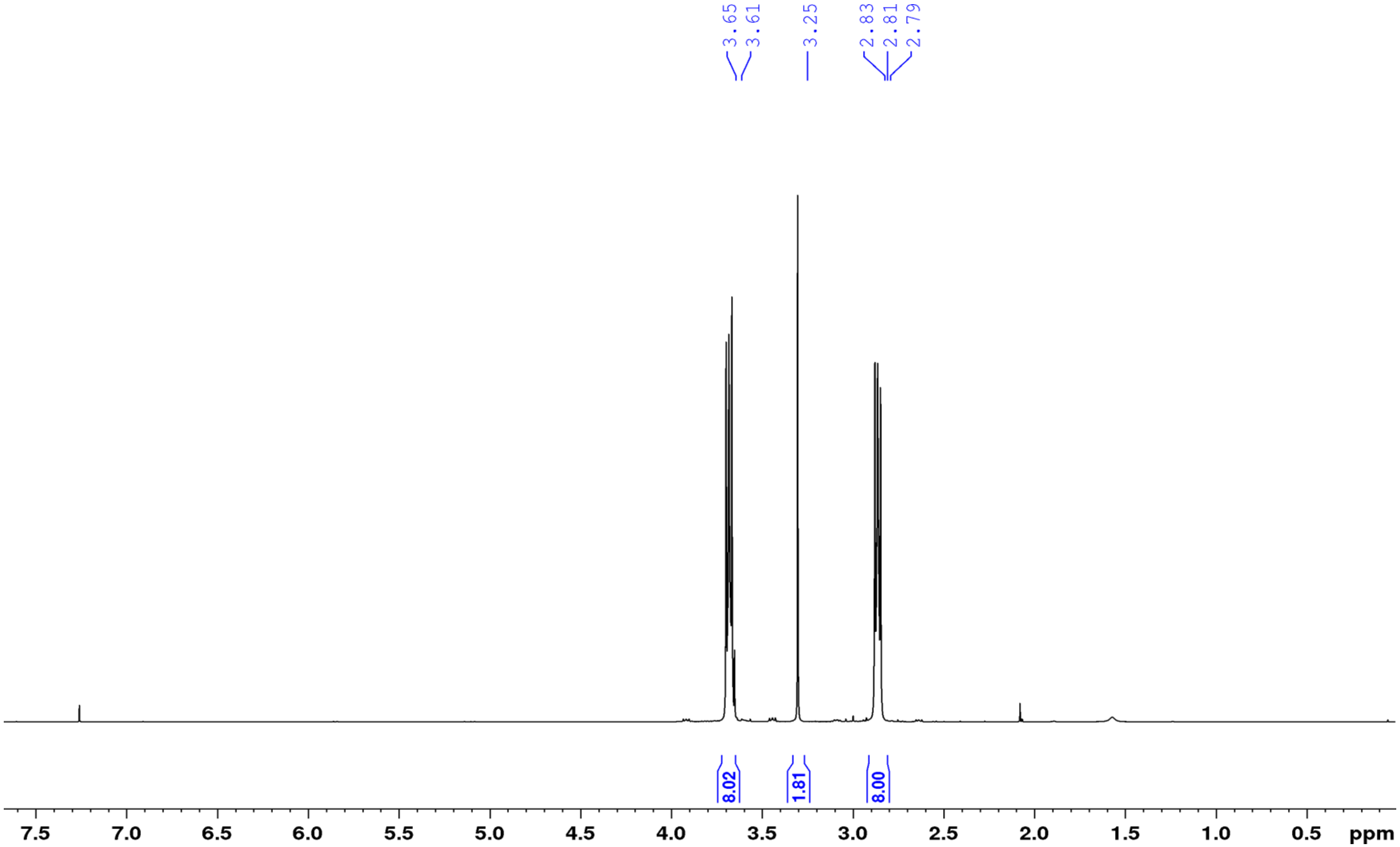

Figure S13: ^{1}H NMR spectrum ($CDCl_3$, 300 MHz, 298 K) of 1,1-dimorpholinoethene (**1**).

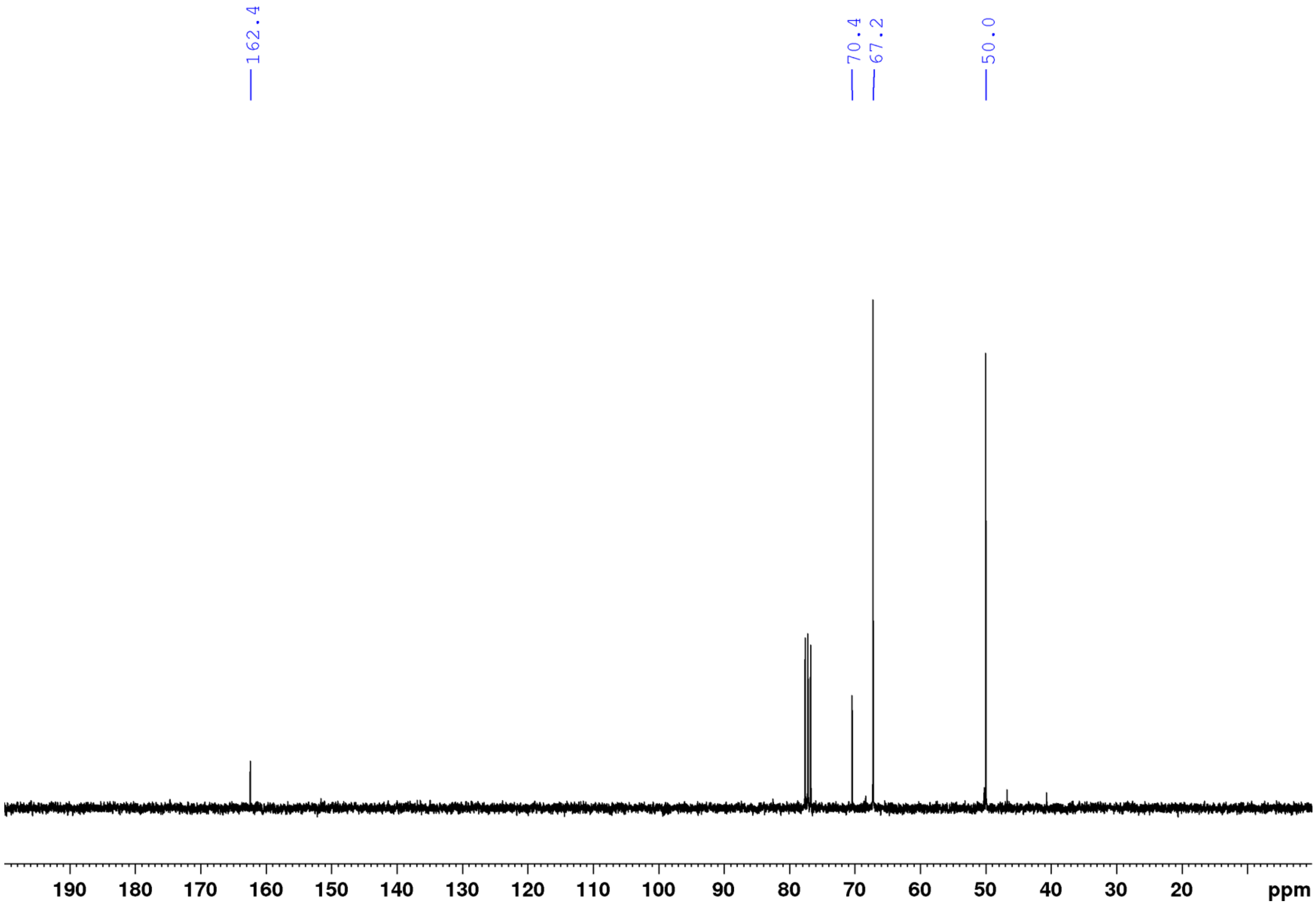

Figure S14: $^{13}C\{^{1}H\}$ NMR spectrum ($CDCl_3$, 76 MHz, 298 K) of 1,1-dimorpholinoethene (**1**).

S2.2 1,1-Dibromo-2,2-dimorpholinoethene (**2**)

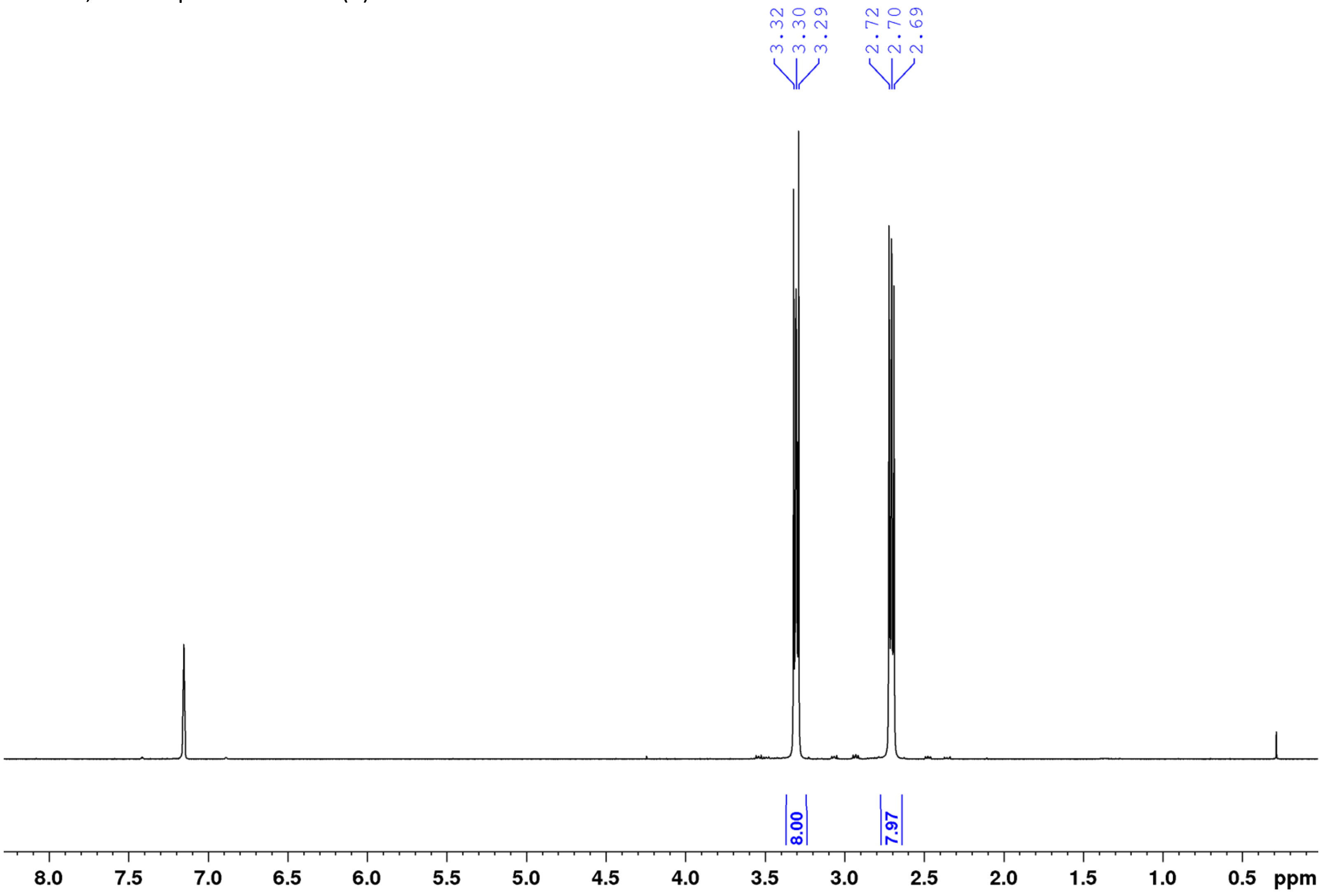

Figure S15: ^{1}H NMR spectrum (C_6D_6, 300 MHz, 298 K) of 1,1-dibromo-2,2-dimorpholinoethene (**2**).

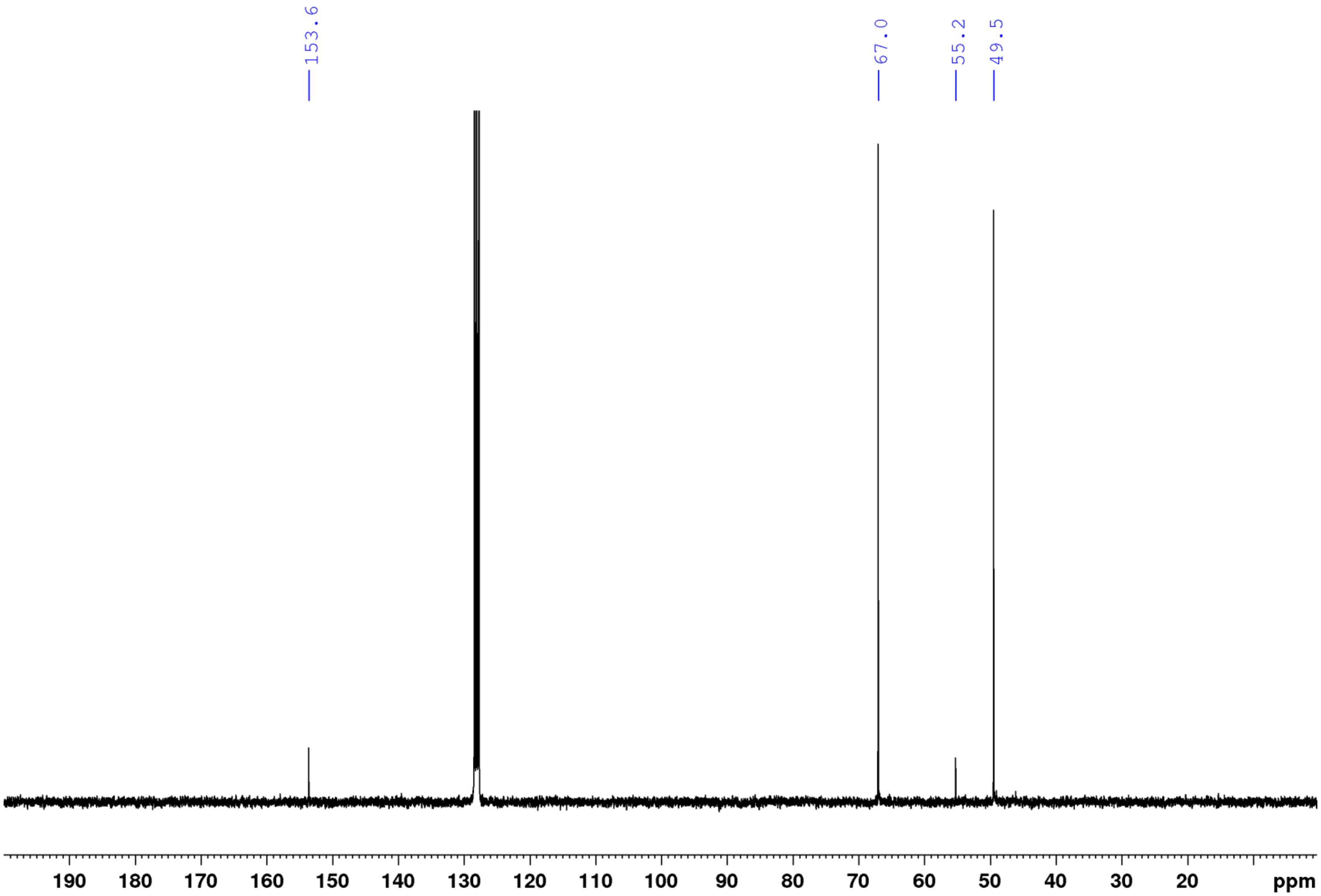

Figure S16: $^{13}C\{^{1}H\}$ NMR spectrum (C_6D_6, 76 MHz, 298 K) of 1,1-dibromo-2,2-dimorpholinoethene (**2**).

S2.3 Dimorpholinoacetylene (**3**)

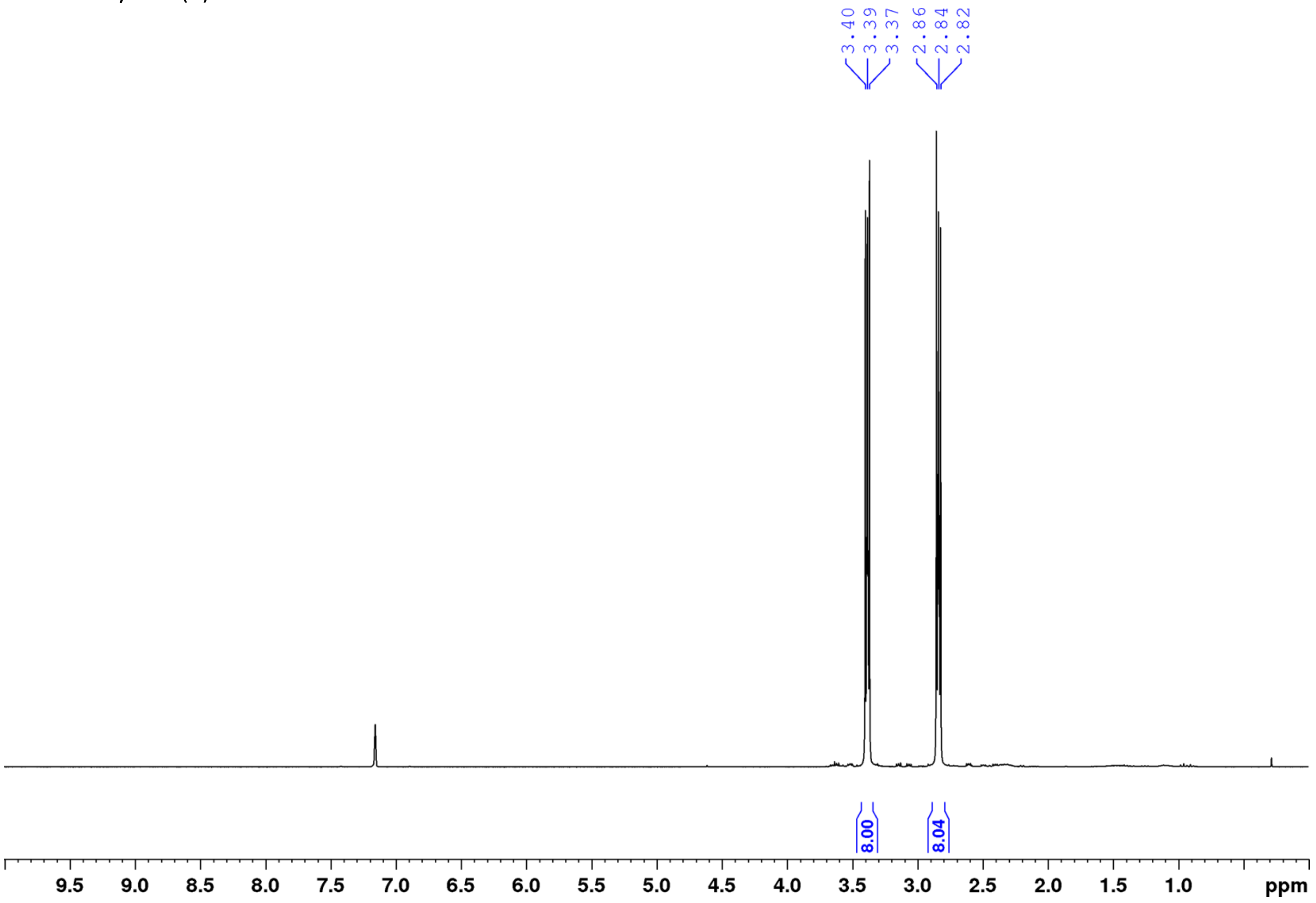

Figure 17: ^{1}H NMR spectrum (C_6D_6, 300 MHz, 298 K) of 1,1-dibromo-2,2-dimorpholinoethene (**3**).

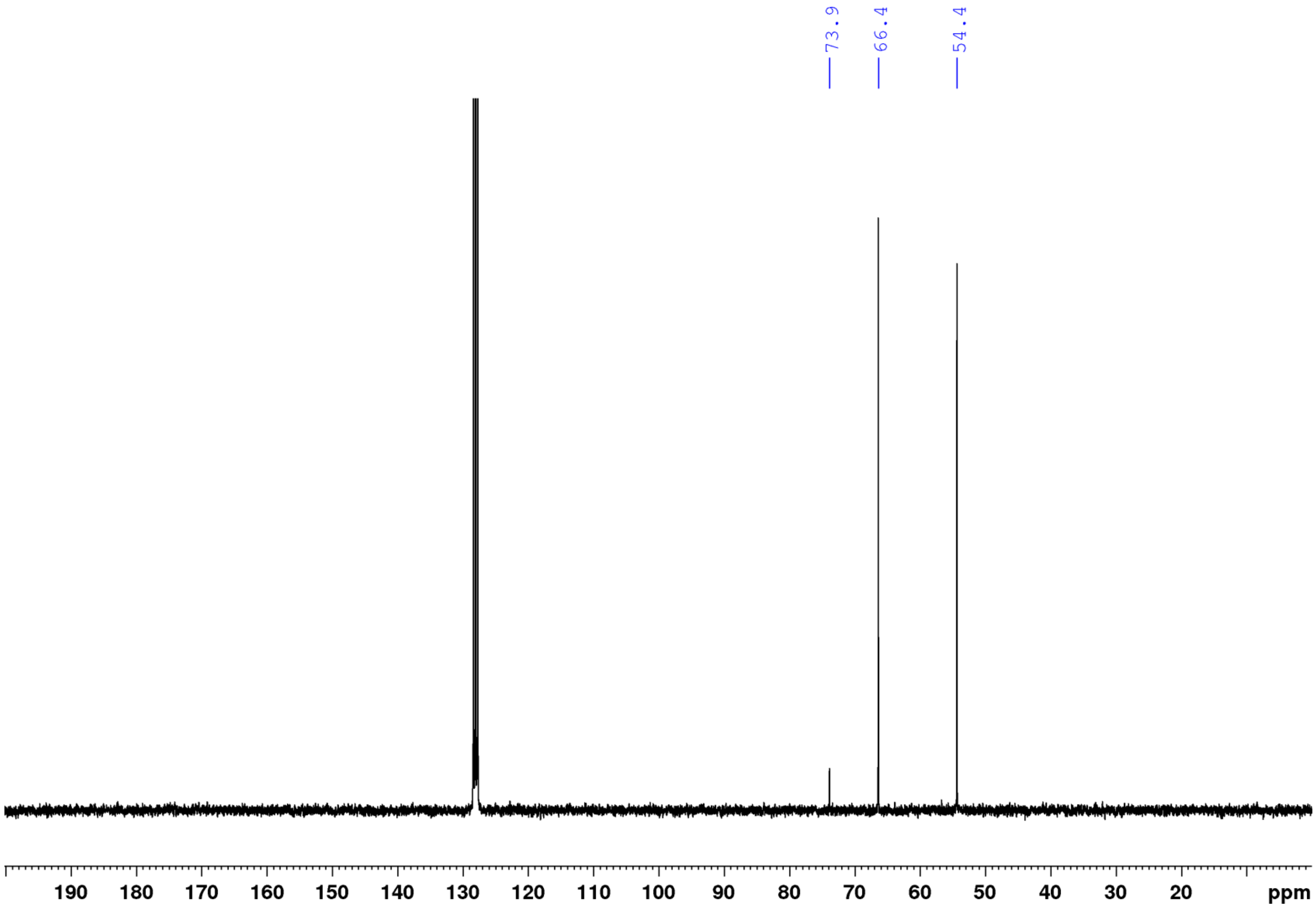

Figure S18: $^{13}C\{^{1}H\}$ NMR spectrum (C_6D_6, 76 MHz, 298 K) of dimorpholinoacetylene (**3**).

S2.4 1,3,4,4-Tetramorpholinobut-1-yn-3-ene (**4**)

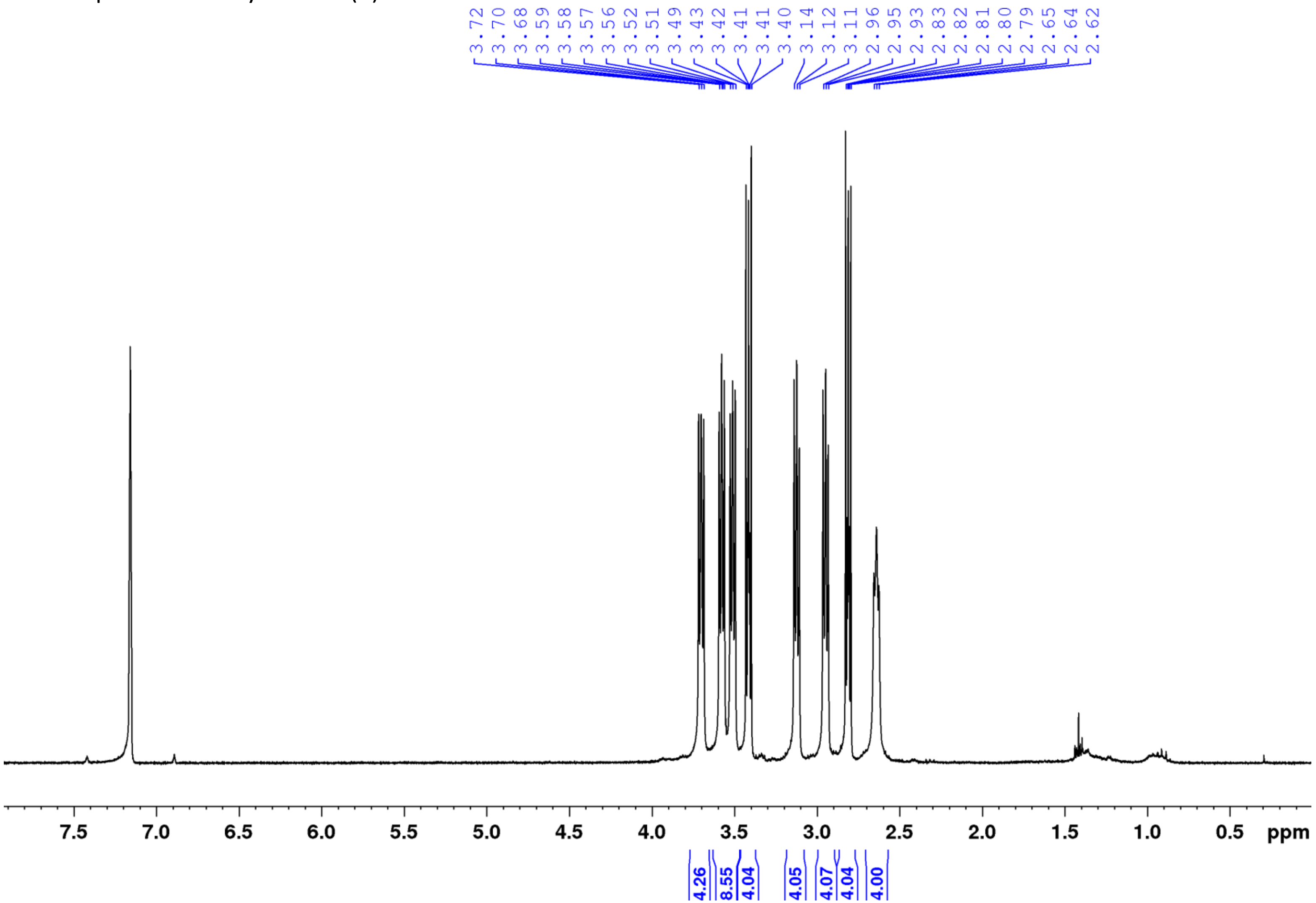

Figure S19: ^{1}H NMR spectrum (C_6D_6, 300 MHz, 298 K) of 1,3,4,4-tetramorpholinobut-1-yn-3-ene (**4**).

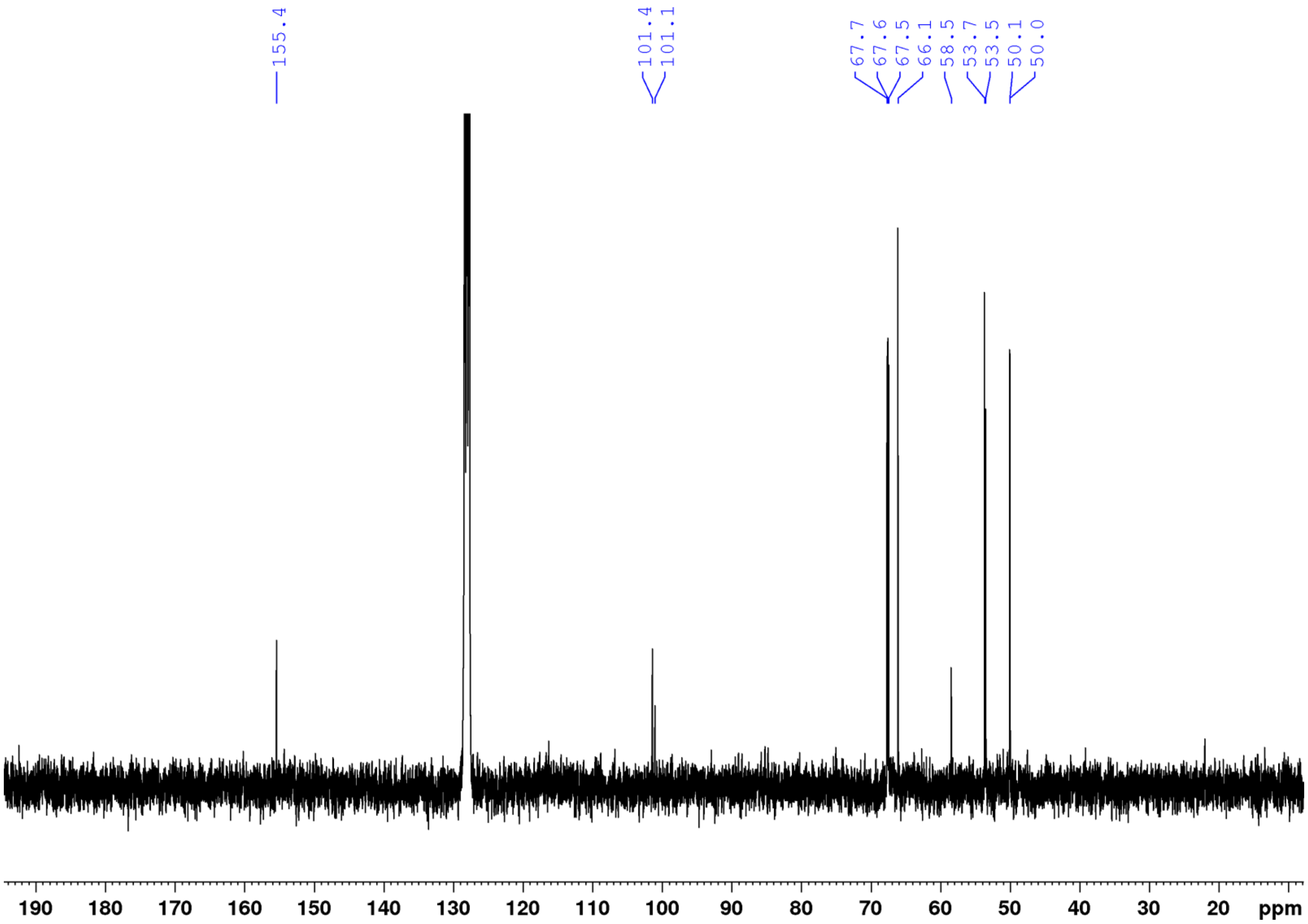

Figure S20: $^{13}C\{^{1}H\}$ NMR spectrum (C_6D_6, 76 MHz, 298 K) of 1,3,4,4-tetramorpholinobut-1-yn-3-ene (**4**).

S2.5 (CBA)AuCl (**5**)

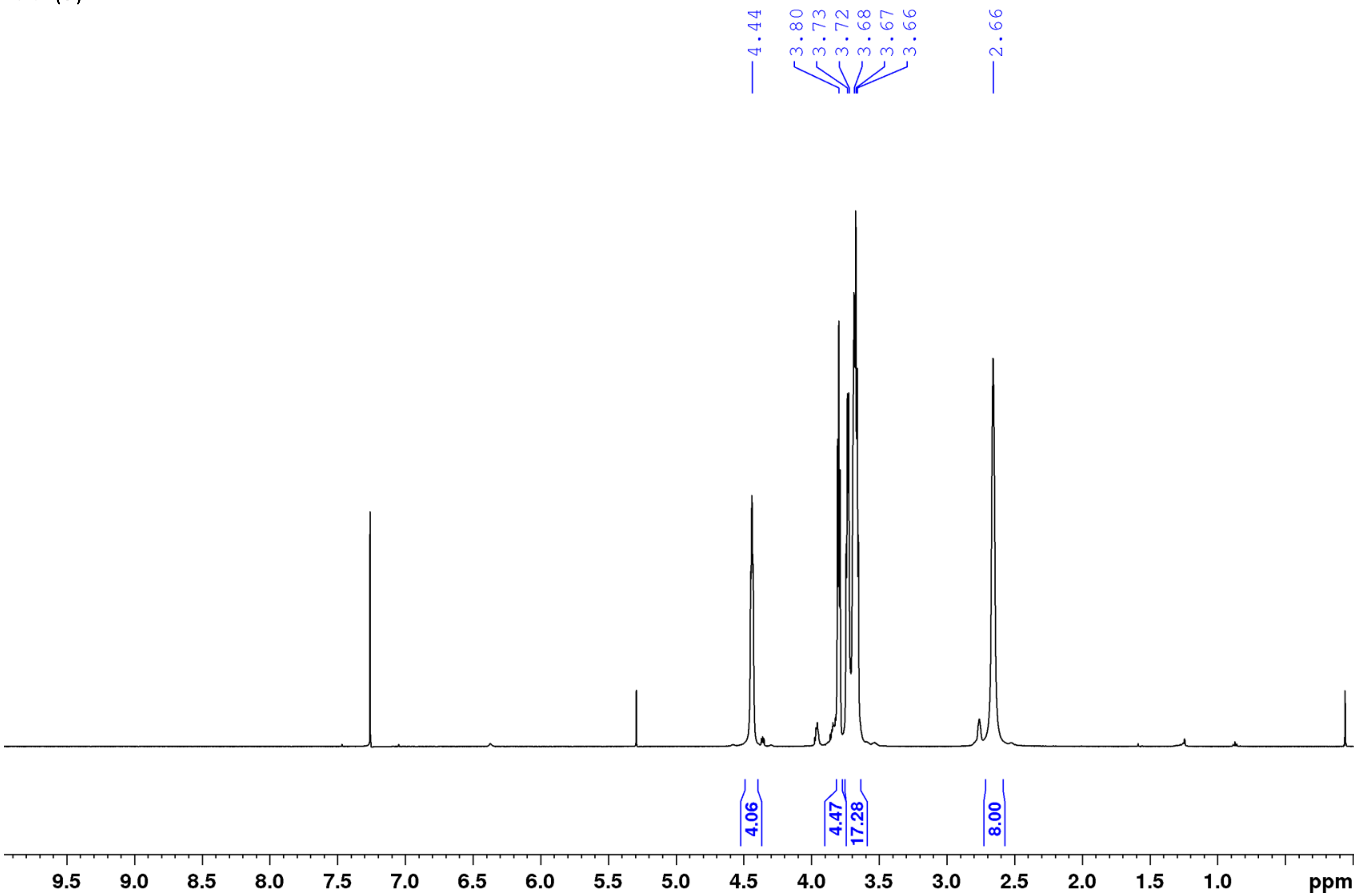

Figure S21: ^{1}H NMR spectrum ($CDCl_3$, 500 MHz, 298 K) of (CBA)AuCl (**5**).

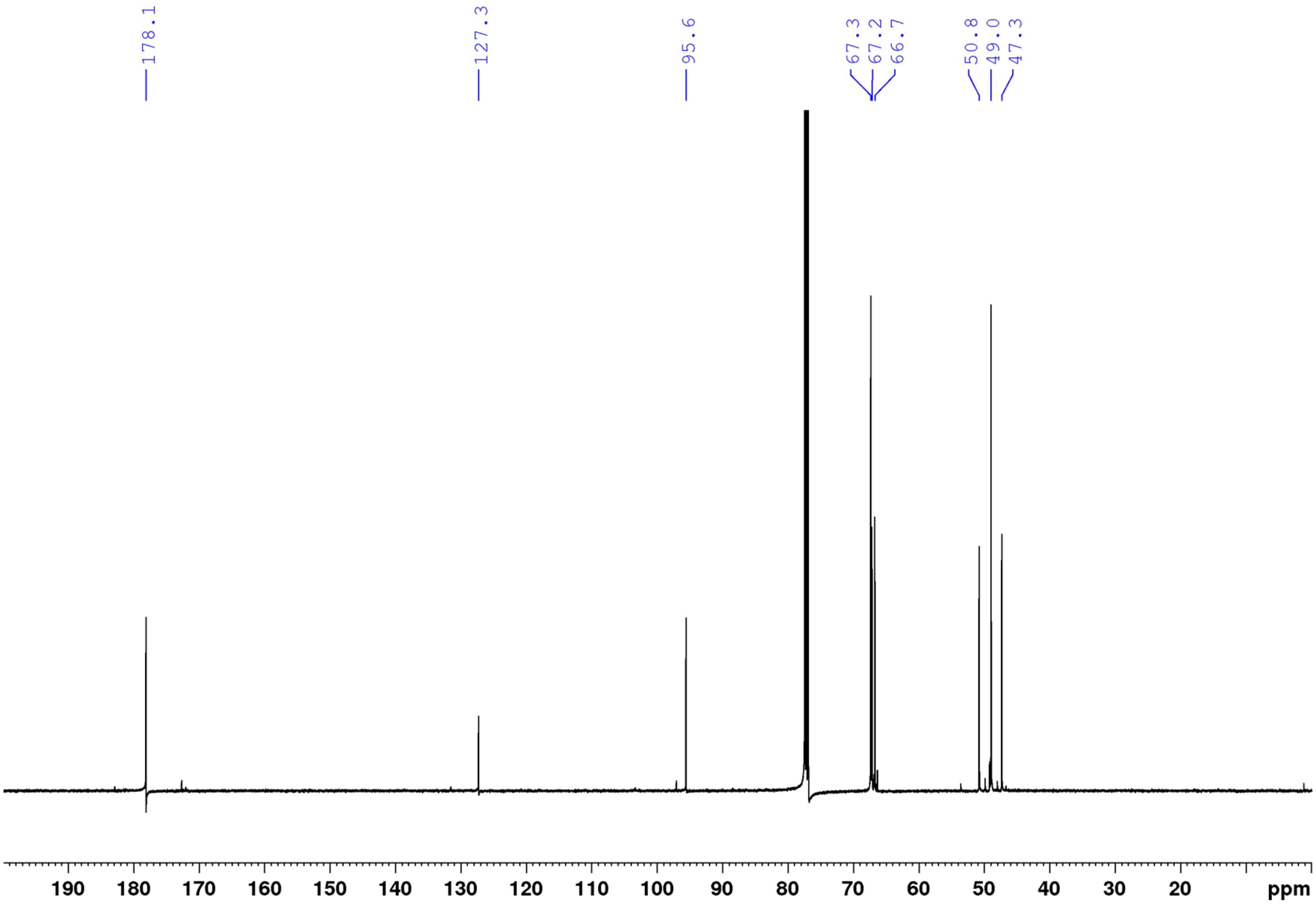

Figure S22: $^{13}C\{^1H\}$ NMR spectrum ($CDCl_3$, 126 MHz, 298 K) of (CBA)AuCl (**5**).

S2.6 (CBA)Rh(COD)Cl (**6**)

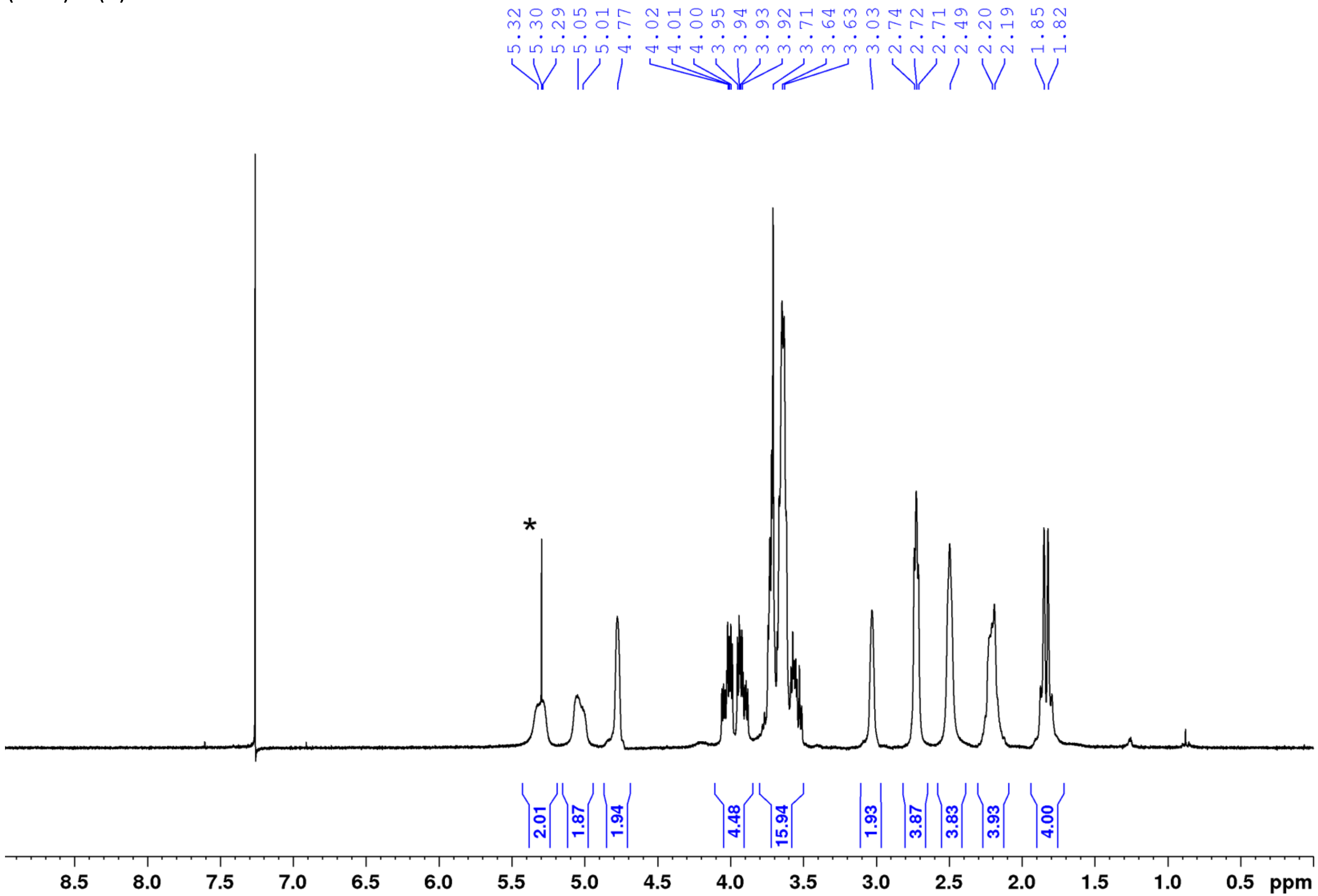

Figure S23: ^{1}H NMR spectrum ($CDCl_3$, 300 MHz, 298 K) of (CBA)Rh(COD)Cl (**6**), * = residual DCM.

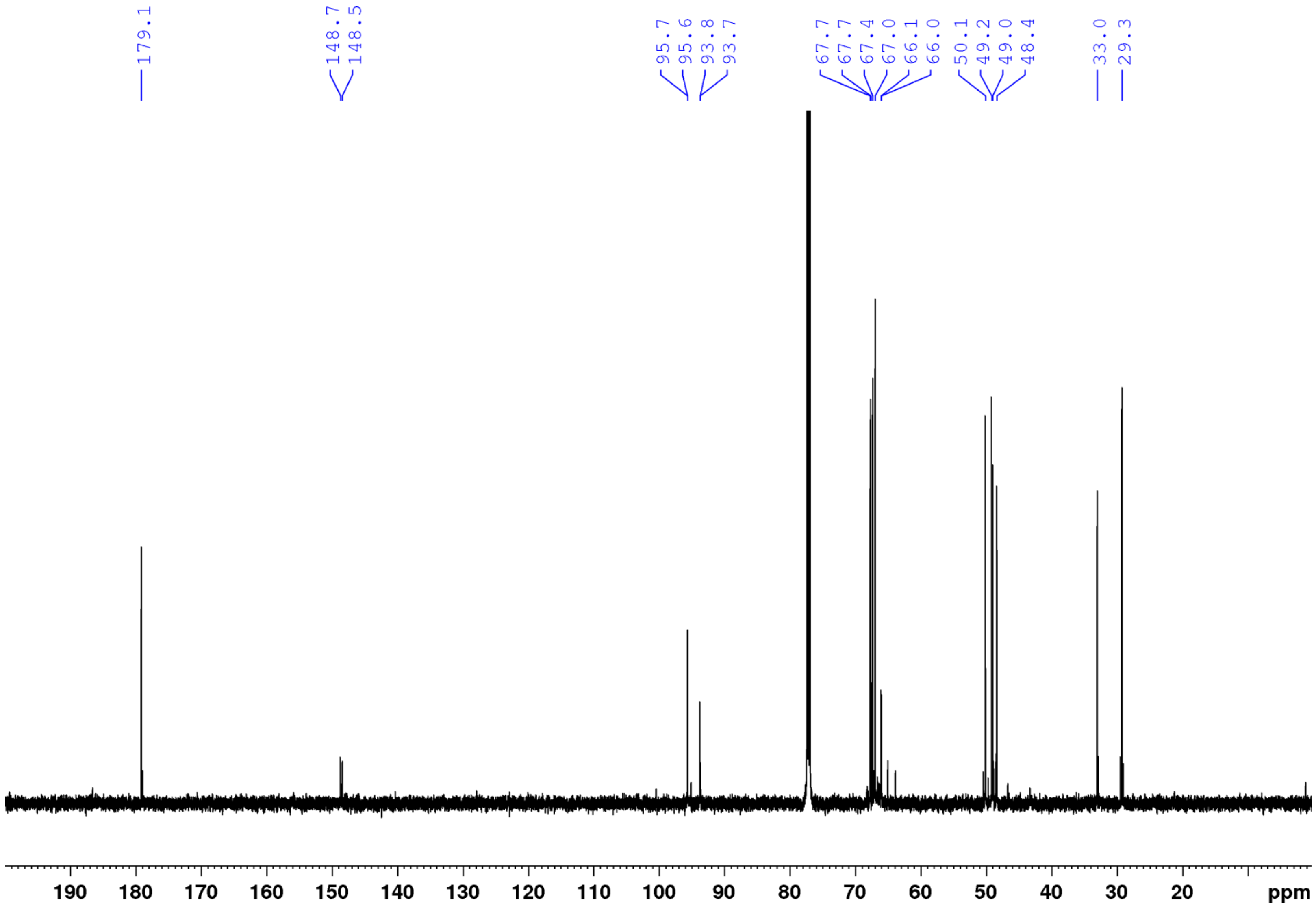

Figure S24: $^{13}C\{^1H\}$ NMR spectrum ($CDCl_3$, 126 MHz, 298 K) of (CBA)Rh(COD)Cl (**6**).

S2.7 (CBA)Rh(CO)$_2$Cl (**7**)

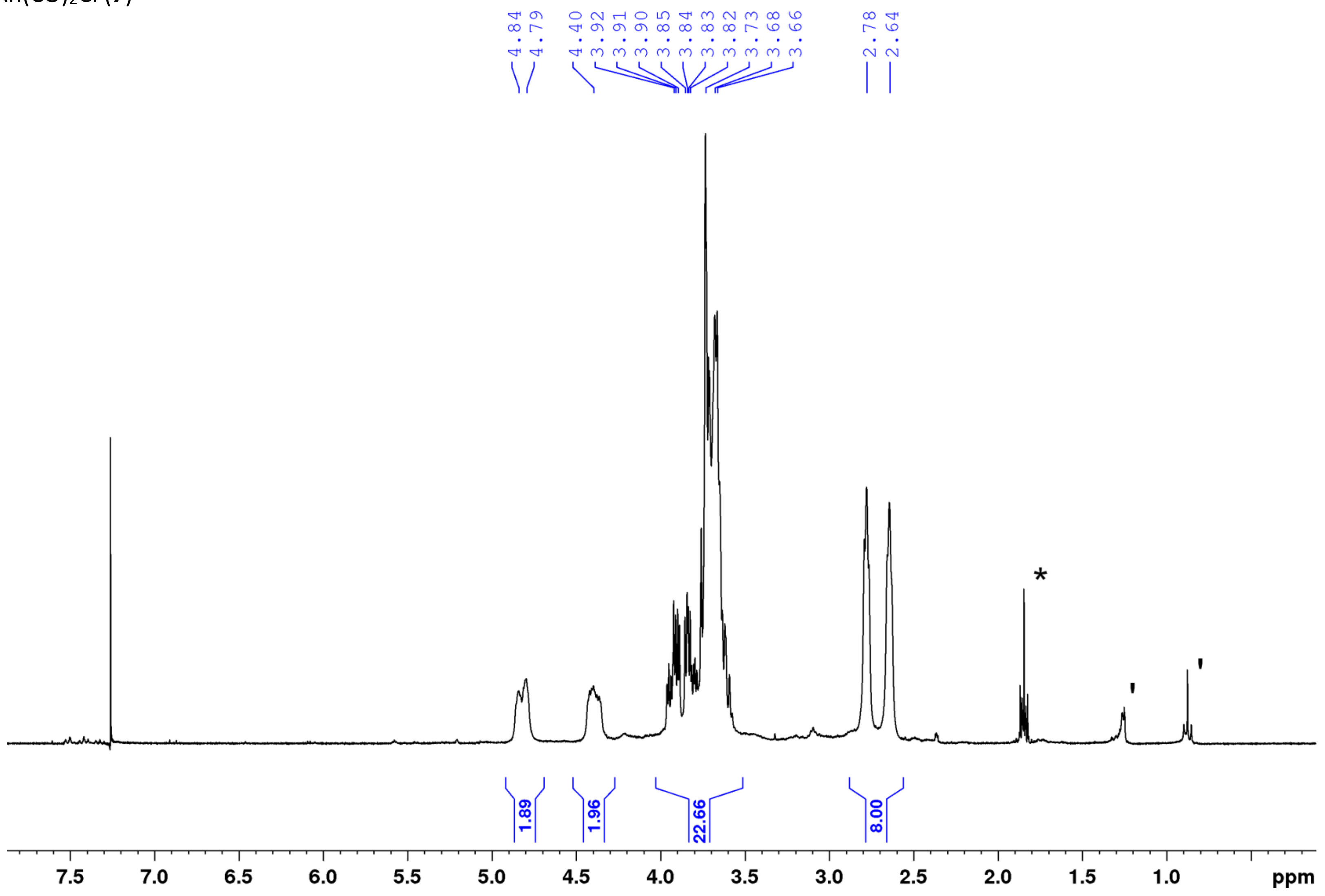

Figure S25: : ^{1}H NMR spectrum ($CDCl_3$, 300 MHz, 298 K) of (CBA)Rh(CO)$_2$Cl (**7**); * = residual THF, ' = residual n-hexane.

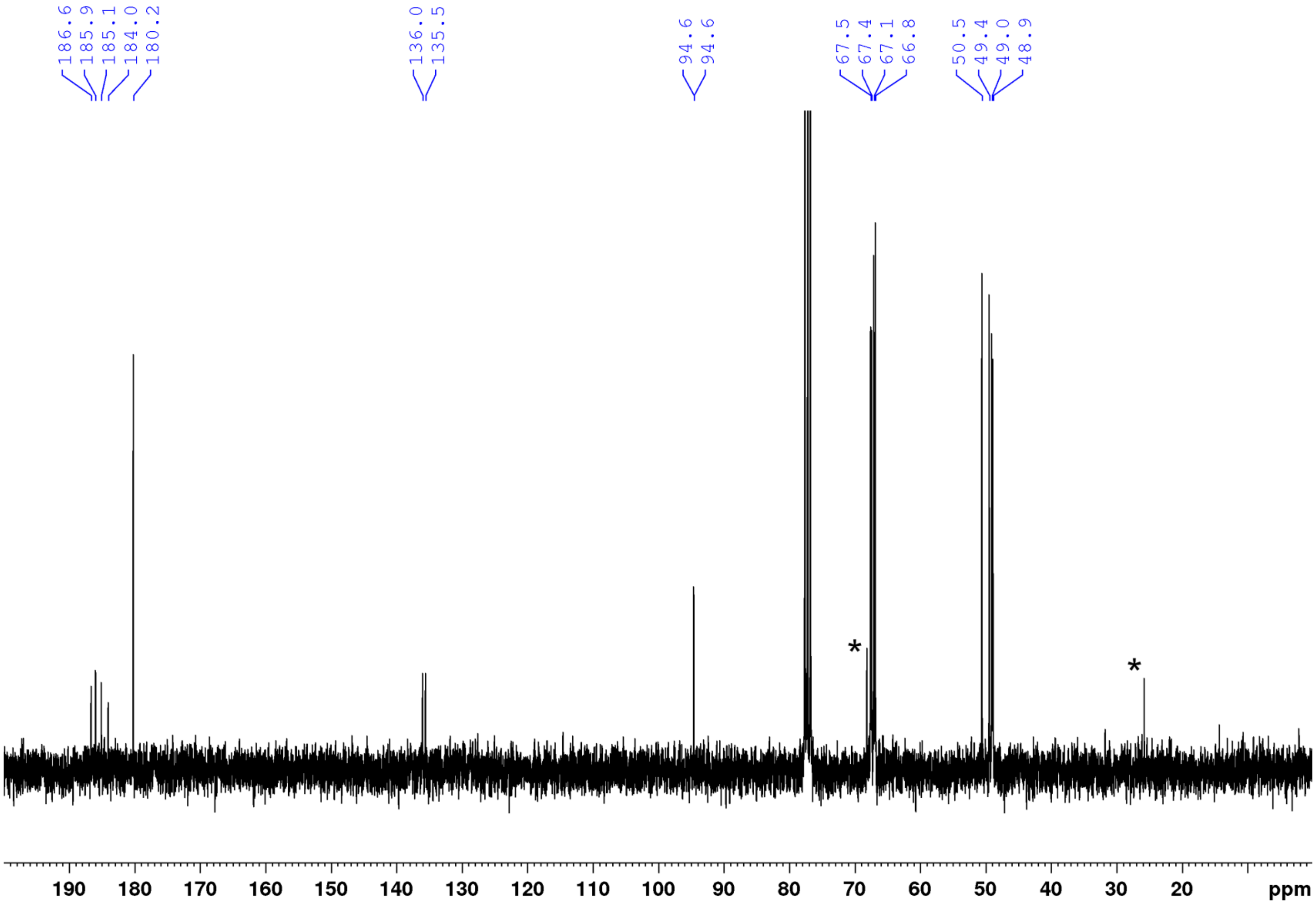

Figure S26: $^{13}C\{^{1}H\}$ NMR spectrum ($CDCl_3$, 76 MHz, 298 K) of (CBA)Rh$(CO)_2$Cl (**7**); * = residual THF.

S.2.8 1,2,3,4-Tetramorpholinocyclobutadiene-1-hydrobromide **(8)**

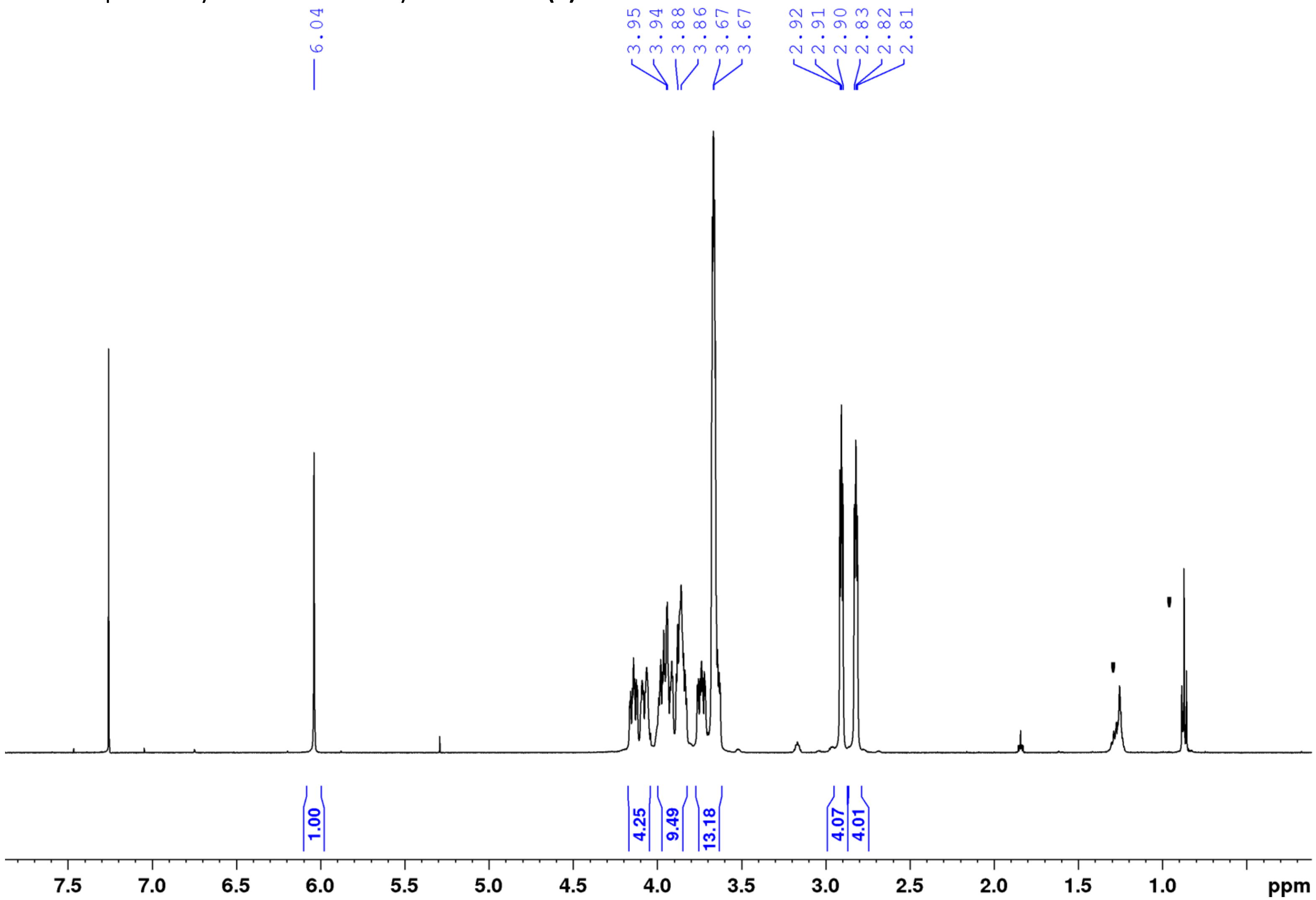

Figure S27: ^{1}H NMR spectrum ($CDCl_3$, 500 MHz, 298 K) of 1,2,3,4-tetramorpholinocyclobutenylium bromide (**8**); ʻ = residual *n*-hexane.

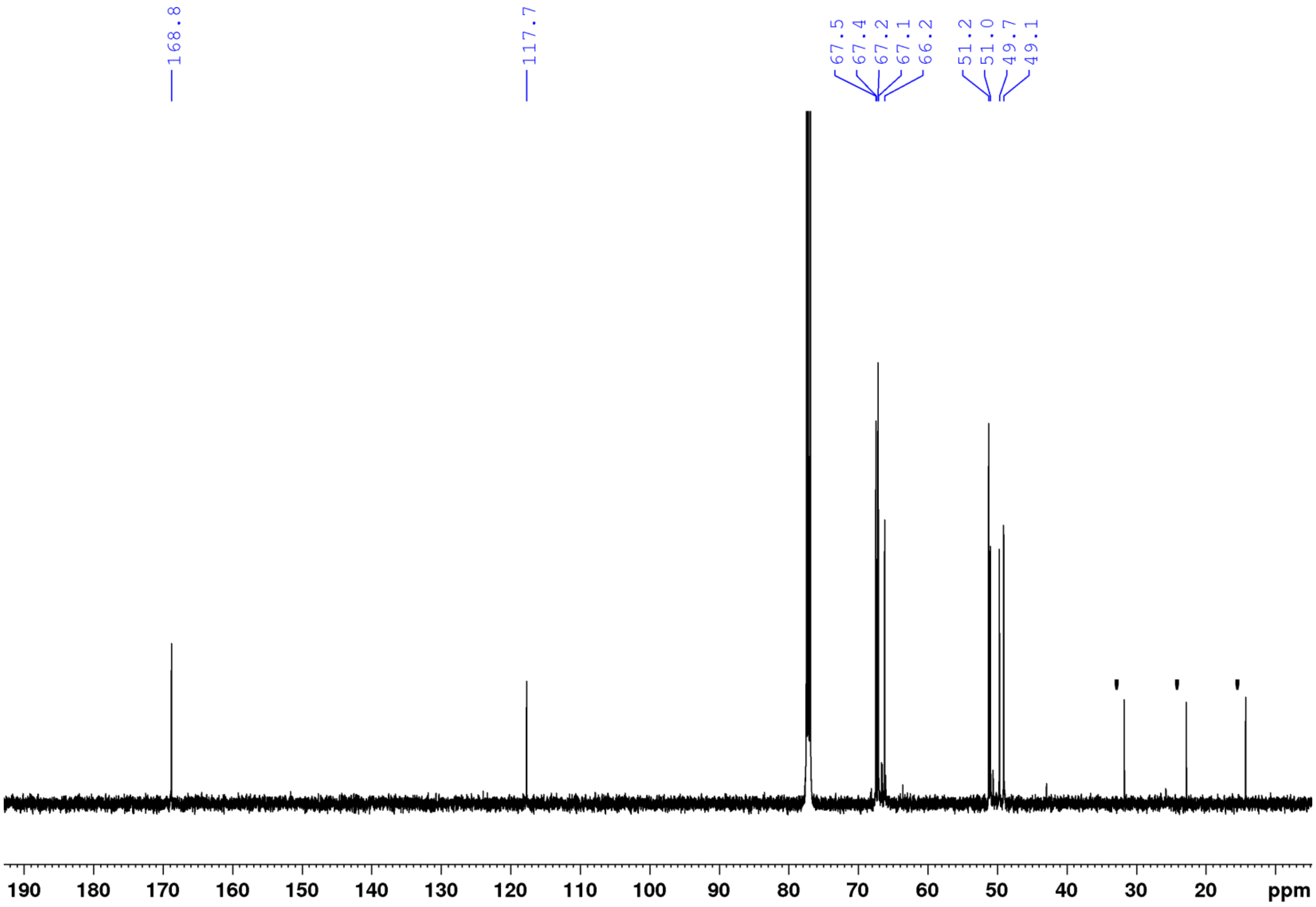

Figure S28: $^{13}C\{^1H\}$ NMR spectrum ($CDCl_3$, 126 MHz, 298 K) of 1,2,3,4-tetramorpholinocyclobutenylium bromide (**8**); ' = residual *n*-hexane.

S.2.9 Tetramorpholinocyclobutenediylium bis(tribromide) (**9**).

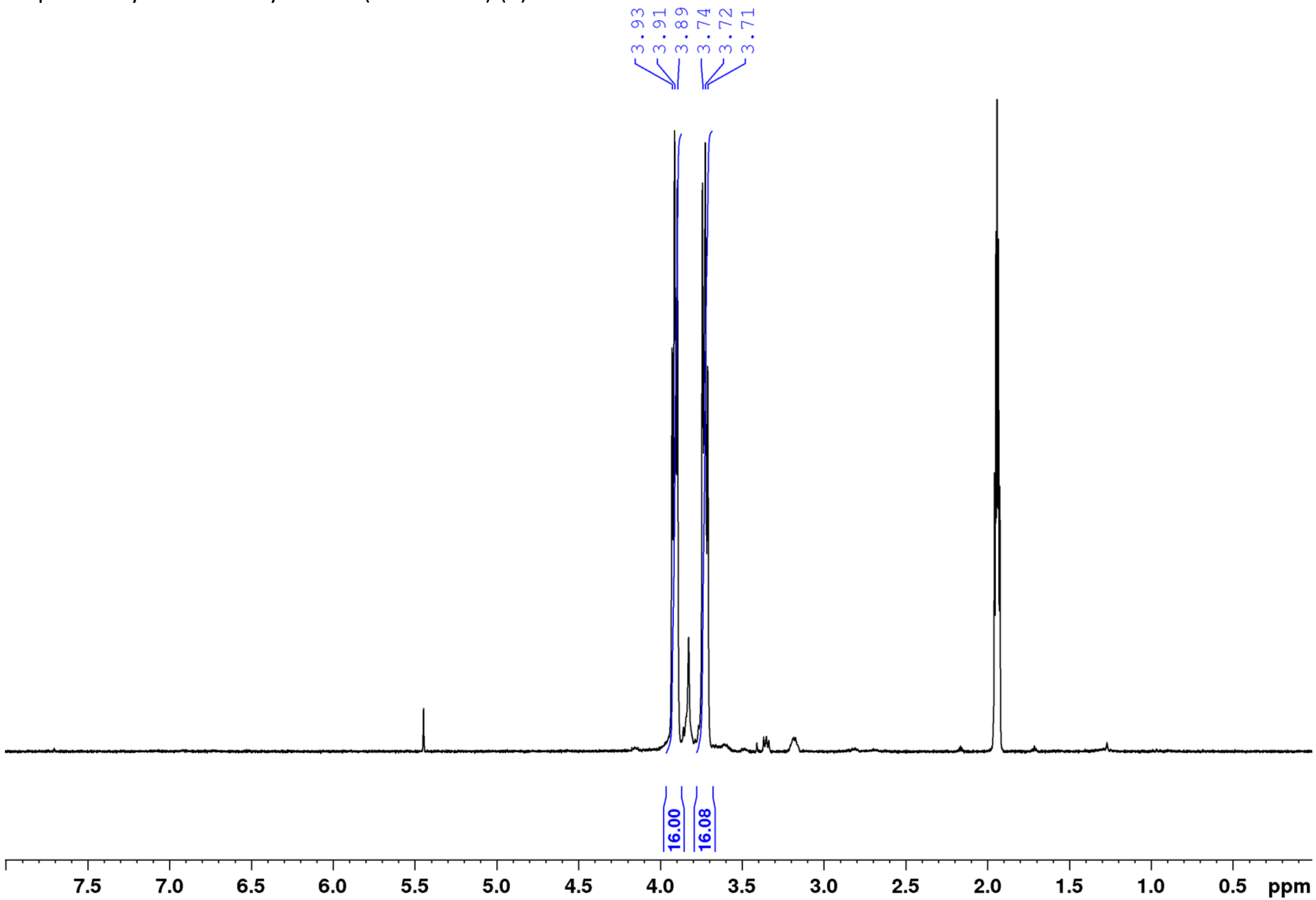

Figure S29: ^{1}H NMR spectrum (CD_3CN, 300 MHz, 298 K) of tetramorpholinocyclobutendiylium bis(tribromide) (**9**).

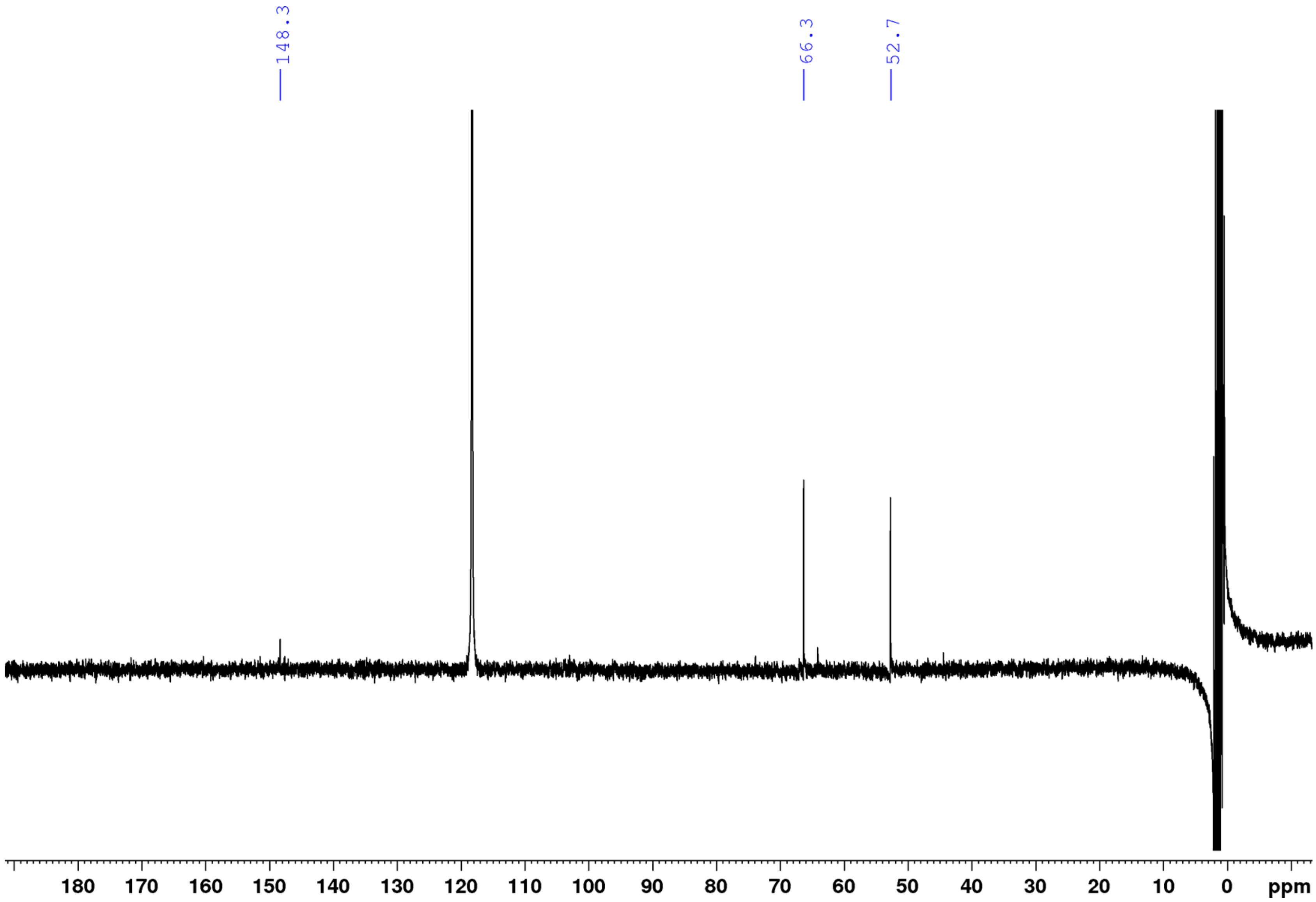

Figure S30: $^{13}C\{^{1}H\}$ NMR spectrum (CD_3CN, 76 MHz, 298 K) of tetramorpholinocyclobutendiylium bis(tribromide) (**9**).

S3 Additional computational charge analysis

The table below lists the NBO charges in $C_4R_4^{2+}$ (R = CH_3, NH_2 and NMe_2), and of **9a** and **9b**.

	$C_4Me_4^{2+}$	$C_4(NH_2)_4^{2+}$	$C_4(NMe_2)_4$	**9a**	**9b**
NBO *cyclo*-C	0.26	0.27	0.27	0.19 – 0.37	0.23 – 0.40
NBO substituent	0.25	0.23	0.23	0.13 – 0.31	0.10 – 0.27

S4 Coordinates of optimized structures

Cyclo-$C_4H_4^{2+}$ fully optimized.

Center Number	Atomic Number	Atomic Type	Coordinates (Angstroms) X	Y	Z
1	6	0	0.000000	0.969864	0.187027
2	6	0	-0.969864	0.000000	-0.187027
3	6	0	0.000000	-0.969864	0.187027
4	6	0	0.969864	0.000000	-0.187027
5	1	0	0.000000	1.973823	0.633237
6	1	0	-1.973823	0.000000	-0.633237
7	1	0	0.000000	-1.973823	0.633237
8	1	0	1.973823	0.000000	-0.633237

Cyclo-$C_4H_4^{2+}$ planar

Center Number	Atomic Number	Atomic Type	Coordinates (Angstroms) X	Y	Z
1	6	0	0.000000	0.932880	0.187220
2	6	0	-0.932880	0.000000	-0.187220
3	6	0	0.000000	-0.932880	0.187220
4	6	0	0.932880	0.000000	-0.187220
5	1	0	0.000000	1.887020	0.640770
6	1	0	-1.887020	0.000000	-0.640770
7	1	0	0.000000	-1.887020	0.640770
8	1	0	1.887020	0.000000	-0.640770

Cyclo-$C_4Me_4^{2+}$ fully optimized.

Center Number	Atomic Number	Atomic Type	Coordinates (Angstroms) X	Y	Z
1	6	0	0.000000	1.005557	-0.128662
2	6	0	-1.005557	0.000000	0.128662
3	6	0	0.000000	-1.005557	-0.128662
4	6	0	1.005557	0.000000	0.128662
5	6	0	0.000000	-2.410058	-0.497455
6	1	0	0.000000	-2.971844	0.460879
7	1	0	-0.905576	-2.705975	-1.031919
8	1	0	0.905576	-2.705975	-1.031919
9	6	0	-2.410058	0.000000	0.497455
10	1	0	-2.971844	0.000000	-0.460879
11	1	0	-2.705975	-0.905576	1.031919
12	1	0	-2.705975	0.905576	1.031919
13	6	0	0.000000	2.410058	-0.497455
14	1	0	-0.905576	2.705975	-1.031919
15	1	0	0.000000	2.971844	0.460879
16	1	0	0.905576	2.705975	-1.031919
17	6	0	2.410058	0.000000	0.497455
18	1	0	2.971844	0.000000	-0.460879
19	1	0	2.705975	0.905576	1.031919
20	1	0	2.705975	-0.905576	1.031919

Cyclo-$C_4Me_4^{2+}$ planar

Center Number	Atomic Number	Atomic Type	Coordinates (Angstroms) X	Y	Z
1	6	0	0.000001	0.726522	0.726542
2	6	0	-0.000001	0.726516	-0.726495
3	6	0	0.000001	-0.726516	-0.726495
4	6	0	-0.000001	-0.726522	0.726542
5	6	0	-0.026087	-1.749114	-1.749153
6	1	0	-1.094621	-1.965275	-1.965513
7	1	0	0.410163	-1.410076	-2.692855
8	1	0	0.409977	-2.692908	-1.410148
9	6	0	0.026087	1.749114	-1.749153
10	1	0	1.094621	1.965275	-1.965513
11	1	0	-0.410163	1.410076	-2.692855
12	1	0	-0.409977	2.692908	-1.410148
13	6	0	-0.026115	1.749179	1.749140
14	1	0	0.410168	2.692880	1.410136
15	1	0	-1.094664	1.965548	1.965213
16	1	0	0.409883	1.410180	2.692966
17	6	0	0.026115	-1.749179	1.749140
18	1	0	1.094664	-1.965548	1.965213
19	1	0	-0.409883	-1.410180	2.692966
20	1	0	-0.410168	-2.692880	1.410136

Cyclo-$C_4(NH_2)_4^{2+}$

Center Number	Atomic Number	Atomic Type	Coordinates (Angstroms) X	Y	Z
1	6	0	-0.196366	-1.004417	-0.000037
2	6	0	1.004420	-0.196367	0.000162
3	6	0	0.196366	1.004417	0.000037
4	6	0	-1.004420	0.196366	-0.000162
5	7	0	0.447722	2.289658	0.000046
6	7	0	2.289669	-0.447719	0.000197
7	7	0	-0.447721	-2.289658	-0.000045
8	7	0	-2.289669	0.447719	-0.000198
9	1	0	-0.289409	2.986808	-0.000083
10	1	0	1.393283	2.657400	-0.000236
11	1	0	2.986816	0.289413	-0.000740
12	1	0	2.657415	-1.393278	-0.000682
13	1	0	0.289409	-2.986808	0.000086
14	1	0	-1.393282	-2.657400	0.000234
15	1	0	-2.986816	-0.289413	0.000738
16	1	0	-2.657415	1.393278	0.000683

Cyclo-$C_4(Me_2)_4^{2+}$

Center Number	Atomic Number	Atomic Type	Coordinates (Angstroms) X	Y	Z
1	6	0	0.149069	-1.024744	0.000494
2	6	0	1.024745	0.149069	0.000494
3	6	0	-0.149069	1.024744	0.000494

4	6	0	-1.024744	-0.149069	0.000495
5	7	0	-0.338792	2.330108	-0.000321
6	7	0	2.330109	0.338791	-0.000317
7	7	0	0.338791	-2.330108	-0.000321
8	7	0	-2.330108	-0.338791	-0.000316
9	6	0	3.281837	-0.626557	0.584382
10	1	0	3.885234	-1.100935	-0.191234
11	1	0	3.945208	-0.070428	1.250321
12	1	0	2.756986	-1.371127	1.175718
13	6	0	1.535674	-2.966920	-0.584491
14	1	0	2.099143	-2.251979	-1.176959
15	1	0	2.163034	-3.408699	0.191371
16	1	0	1.192202	-3.762440	-1.249304
17	6	0	-0.626556	-3.281838	0.584377
18	1	0	-1.371126	-2.756988	1.175715
19	1	0	-1.100935	-3.885233	-0.191239
20	1	0	-0.070427	-3.945210	1.250313
21	6	0	-2.966921	-1.535675	-0.584486
22	1	0	-2.251982	-2.099144	-1.176955
23	1	0	-3.408698	-2.163035	0.191378
24	1	0	-3.762442	-1.192204	-1.249297
25	6	0	-3.281837	0.626555	0.584383
26	1	0	-2.756986	1.371126	1.175718
27	1	0	-3.885234	1.100933	-0.191233
28	1	0	-3.945207	0.070426	1.250321
29	6	0	-1.535676	2.966920	-0.584490
30	1	0	-2.099146	2.251979	-1.176959
31	1	0	-2.163036	3.408697	0.191373
32	1	0	-1.192206	3.762440	-1.249304
33	6	0	0.626554	3.281840	0.584375
34	1	0	0.070424	3.945212	1.250311
35	1	0	1.371125	2.756992	1.175713

36	1	0	1.100932	3.885234	-0.191243
37	6	0	2.966925	1.535674	-0.584483
38	1	0	3.762446	1.192203	-1.249294
39	1	0	2.251987	2.099146	-1.176953
40	1	0	3.408700	2.163033	0.191382

9a

Center Number	Atomic Number	Atomic Type	Coordinates (Angstroms) X	Y	Z
1	7	0	1.656984	1.675612	0.021457
2	7	0	1.656979	-1.675607	-0.021458
3	7	0	-1.656978	-1.675608	0.021397
4	7	0	-1.656983	1.675613	-0.021396
5	6	0	0.733235	0.733145	0.004676
6	6	0	0.733234	-0.733132	-0.004676
7	6	0	-0.733232	-0.733132	0.004649
8	6	0	-0.733233	0.733144	-0.004649
9	6	0	1.438131	3.017083	0.622011
10	1	0	0.516242	3.008922	1.199903
11	1	0	1.382463	3.764934	-0.172325
12	6	0	2.626957	3.340075	1.537743
13	1	0	2.519751	4.356131	1.919026
14	1	0	2.649850	2.644837	2.389587
15	6	0	4.106422	1.992454	0.323358
16	1	0	5.049414	2.040959	-0.221874
17	1	0	4.223770	1.288465	1.160230
18	6	0	2.989628	1.543192	-0.621868
19	1	0	2.972072	2.208508	-1.490984
20	1	0	3.124969	0.528776	-0.986002

21	6	0	2.989618	-1.543210	0.621884
22	1	0	2.972043	-2.208538	1.490990
23	1	0	3.124967	-0.528801	0.986033
24	6	0	4.106417	-1.992473	-0.323336
25	1	0	5.049402	-2.040999	0.221906
26	1	0	4.223784	-1.288473	-1.160197
27	6	0	2.626948	-3.340058	-1.537758
28	1	0	2.519735	-4.356105	-1.919060
29	1	0	2.649857	-2.644804	-2.389589
30	6	0	1.438117	-3.017069	-0.622031
31	1	0	0.516231	-3.008893	-1.199929
32	1	0	1.382438	-3.764930	0.172296
33	6	0	-1.438118	-3.017090	0.621928
34	1	0	-0.516231	-3.008934	1.199824
35	1	0	-1.382441	-3.764925	-0.172424
36	6	0	-2.626948	-3.340104	1.537646
37	1	0	-2.649853	-2.644877	2.389499
38	1	0	-2.519738	-4.356164	1.918916
39	6	0	-4.106416	-1.992478	0.323269
40	1	0	-5.049402	-2.040984	-0.221972
41	1	0	-4.223780	-1.288504	1.160153
42	6	0	-2.989619	-1.543187	-0.621938
43	1	0	-2.972047	-2.208488	-1.491066
44	1	0	-3.124965	-0.528766	-0.986054
45	6	0	-2.989628	1.543169	0.621923
46	1	0	-2.972075	2.208458	1.491059
47	1	0	-3.124968	0.528741	0.986023
48	6	0	-4.106421	1.992459	-0.323291
49	1	0	-4.223766	1.288496	-1.160187
50	1	0	-5.049414	2.040944	0.221940
51	6	0	-2.626957	3.340122	-1.537631
52	1	0	-2.649846	2.644910	-2.389497

53	1	0	-2.519753	4.356189	-1.918882
54	6	0	-1.438132	3.017104	-0.621907
55	1	0	-0.516242	3.008964	-1.199798
56	1	0	-1.382467	3.764929	0.172454
57	8	0	3.839442	-3.291041	-0.813929
58	8	0	-3.839460	3.291054	-0.813820
59	8	0	-3.839444	-3.291062	0.813821
60	8	0	3.839458	3.291033	0.813927

9b

Center Number	Atomic Number	Atomic Type	Coordinates (Angstroms) X	Y	Z
1	6	0	-1.094239	0.018198	0.486220
2	6	0	-0.003960	0.973179	0.237880
3	6	0	0.948414	-0.101635	0.474185
4	6	0	-0.149734	-1.075275	0.279466
5	7	0	2.225141	-0.095238	0.839235
6	7	0	0.077806	2.202452	-0.219862
7	7	0	-2.310748	0.101318	0.969702
8	7	0	-0.320241	-2.345211	-0.047605
9	6	0	1.372443	2.887410	-0.485802
10	1	0	1.400933	3.782908	0.140171
11	1	0	2.194232	2.230607	-0.224181
12	6	0	-1.088694	2.947782	-0.765849
13	1	0	-1.978555	2.326374	-0.714793
14	1	0	-1.227441	3.849194	-0.163567
15	6	0	-2.918142	1.350322	1.504214
16	1	0	-2.988876	1.222765	2.588133
17	1	0	-2.280754	2.205634	1.306023

18	6	0	-3.170119	-1.069119	1.293005
19	1	0	-2.729470	-1.985344	0.919767
20	1	0	-3.230995	-1.132179	2.382919
21	6	0	-1.419169	-2.779050	-0.954299
22	1	0	-1.995079	-3.563813	-0.457682
23	1	0	-2.065739	-1.933497	-1.188410
24	6	0	0.655109	-3.434775	0.191128
25	1	0	1.445162	-3.091487	0.850392
26	1	0	0.106192	-4.223485	0.713267
27	6	0	3.143399	-1.237360	0.628003
28	1	0	3.157867	-1.867291	1.523859
29	1	0	2.808336	-1.808714	-0.234925
30	6	0	2.729832	0.880944	1.852344
31	1	0	2.068792	1.745860	1.895907
32	1	0	2.689938	0.373999	2.822294
33	6	0	1.479938	3.280422	-1.963616
34	1	0	1.565491	2.379744	-2.589872
35	1	0	2.372614	3.890169	-2.106728
36	6	0	-0.829956	3.331322	-2.230001
37	1	0	-0.806241	2.425839	-2.855104
38	1	0	-1.640432	3.973496	-2.576759
39	6	0	-4.323877	1.511695	0.918481
40	1	0	-4.269805	1.669581	-0.169601
41	1	0	-4.816070	2.372694	1.371347
42	6	0	-4.565890	-0.822907	0.709654
43	1	0	-4.516266	-0.793085	-0.389231
44	1	0	-5.236200	-1.629550	1.007248
45	6	0	-0.793994	-3.336186	-2.241958
46	1	0	-0.275780	-2.530606	-2.784244
47	1	0	-1.579623	-3.739755	-2.882035
48	6	0	1.175375	-3.985702	-1.139124
49	1	0	1.778751	-3.229377	-1.664786

50	1	0	1.794935	-4.865261	-0.962735
51	6	0	4.554859	-0.699939	0.363746
52	1	0	4.576793	-0.153494	-0.590470
53	1	0	5.253319	-1.535036	0.303098
54	6	0	4.173292	1.279040	1.550766
55	1	0	4.232208	1.887575	0.636092
56	1	0	4.570620	1.865064	2.379901
57	8	0	0.370409	4.057900	-2.358511
58	8	0	-5.113329	0.377822	1.219677
59	8	0	0.091962	-4.396877	-1.952377
60	8	0	4.984473	0.127034	1.425430

S5 References

[1] *CrysAlisPRO, Oxford Diffraction/Agilent Technologies UK Ltd*, Yarnton, England.

[2] G. M. Sheldrick, *Acta Crystaogr., Sect. A: Found. Crystallogr.* **2008**, *64*, 112.

[3] A. Bouvy, Z. Janousek, H. G. Viehe, *Synthesis* **1983**, *1983*, 718.
